# Optimising surface *d* charge of AuPd nanoalloy catalysts for enhanced catalytic activity

**DOI:** 10.1038/s41467-019-09421-5

**Published:** 2019-03-29

**Authors:** Xiaojuan Zhu, Qishui Guo, Yafei Sun, Shangjun Chen, Jian-Qiang Wang, Mengmeng Wu, Wenzhao Fu, Yanqiang Tang, Xuezhi Duan, De Chen, Ying Wan

**Affiliations:** 1 Key Laboratory of Resource Chemistry of Ministry of Education, Shanghai Key Laboratory of Rare Earth Functional Materials, and Department of Chemistry, Shanghai Normal University, Shanghai, 200234 China; 2 Shanghai Synchrotron Radiation Facility, Shanghai Institute of Applied Physics, Chinese Academy of Sciences, Shanghai, 201204 China; 3 State Key Laboratory of Chemical Engineering, East China University of Science and Technology, Shanghai, 200237 China; 4 Department of Chemical Engineering, Norwegian University of Science and Technology, Trondheim, N-7491 Norway

## Abstract

Understanding the catalytic mechanism of bimetallic nanocatalysts remains challenging. Here, we adopt an adsorbate mediated thermal reduction approach to yield monodispersed AuPd catalysts with continuous change of the Pd-Au coordination numbers embedded in a mesoporous carbonaceous matrix. The structure of nanoalloys is well-defined, allowing for a direct determination of the structure-property relationship. The results show that the Pd single atom and dimer are the active sites for the base-free oxidation of primary alcohols. Remarkably, the *d*-orbital charge on the surface of Pd serves as a descriptor to the adsorbate states and hence the catalytic performance. The maximum *d*-charge gain occurred in a composition with 33–50 at% Pd corresponds to up to 9 times enhancement in the reaction rate compared to the neat Pd. The findings not only open an avenue towards the rational design of catalysts but also enable the identification of key steps involved in the catalytic reactions.

## Abstract (web summary)

Understanding the catalytic mechanism of bimetallic nanocatalysts remains challenging. Here the authors demonstrate that the d-orbital charge on the surface of Pd in a well-defined AuPd nanoalloy serves as a descriptor to the adsorbate states and hence the catalytic performance.

## Introduction

Bimetallic catalysts, for example gold-palladium, have been widely applied in aerobic oxidation of primary alcohols, direct synthesis of H_2_O_2_, etc^1–3^. Their catalytic properties are largely governed by the surface electronic structure of transition metals. So far, exploring the effects of the electronic properties on the catalytic performance for alloys is focused on the cutting of well-defined surfaces of bulk alloys or vapour deposition of a metal onto a single-crystal face of the second metal. The determined model alloy structure facilitates the investigation of the alloy effects on the enhancement for the activity and selectivity. Ensemble and ligand effects have been primarily concerned to mirror the properties of surface alloys; that is, the formation of a contiguous palladium domain when diluting a finite amount of Pd with Au, and the charge transfer between Au and Pd or orbital rehybridization of Au due to the heteroatom bonds^4–6^. Substantial efforts have been devoted to identifying a descriptor for the target reactions. In particular, the *d*-band centre relative to the Fermi level, in which the calculated average energy of the projected density of states for the model metal surface *d*-states can be linked to the interaction with adsorbate states and hence the catalytic performance^5,7^. However, the synergistic effects observed in the experiments using the bimetallic NPs are not always directly correlated to their surface alloy counterpart^8^. Obviously, the electronic properties and in turn the adsorption for substrates are closely dependent on the nature of the alloy and if it has a eutectic-like or core-shell structure^8^. Motivated by the success of the *d*-band centre theory as the activity descriptor for metal surfaces, as well as the transition from single-crystal studies to more industrially relevant catalysts, we are seeking a simple and experimentally measurable descriptor that quantitatively correlates the surface electronic structure to the catalytic activity for transition metal nanoalloy catalysts.

We take advantage of the flexibility of the physical and chemical properties of the gold-palladium alloy which have complete miscibility to experimentally examine alloys with continuous concentration of Pd to establish the catalytic properties for the industrially relevant reactions involving strong adsorbates. The thermodynamically stable configuration is an Au_shell_Pd_core_ configuration because of the high segregation energy of Au in Pd^8,9^. The encapsulation of active Pd surface with a relatively inactive Au shell would inhibit the activity in Pd-catalyzed reactions. Hutchings and co-workers first reported that a Au-rich-core/Pd-shell structure on TiO_2_ can be kinetically generated at low temperatures and show high activity in selective oxidation^1^. However, this arrangement is theoretically predicted to undergo a transition to the above thermodynamically stable core-shell reversal above 500 K^8^^,^^10^. Great efforts have been made to obtain compositionally uniform AuPd nanoalloys and the activity can benefit^11–13^. Unfortunately, the bimetallic structures generally suffer from a dynamical structure change during the liquid phase reactions in particular strong-adsorbate-involved processes due to the metal segregation, leaching and re-deposition^14^. Breaking through the thermodynamic favourable structures of bimetallic nanoparticles is a goal that has been long sought after^15,16^, to obtain both the determined structural resolution with the continuously tuning for the metal ligands of the surface, and the high-efficient and stable catalysts with targeted site requirements for sustainable chemical synthesis.

Herein, we provide a direct experimental evidence for the measured *d* charge at surface Pd sites in AuPd nanoparticles as a descriptor, which correlates well with the catalytic activities, with a typical example for the base-free oxidation of primary alcohols. As a consequence, the unambiguous identification of the site requirements for the formation of targeted oxidation products can be resolved. This is based on the stable monodispersed AuPd nanoalloys with determined structural resolution at an atomic level supported on a less-active carrier which can minimize metal oxide interactions, as well as particle size and compositional uniformity effects. The maximum in the *d*-charge gain has been found with 33–50 at% Pd and results in the highest reaction rate for the oxidation of alcohols. The finding represents a very important advance not only for the rational design of catalysts but also for the identification and tracking of key steps involved in the catalytic reactions.

## Results

### Microstructural properties of Au-Pd nanoalloy

**Fig. 1:**
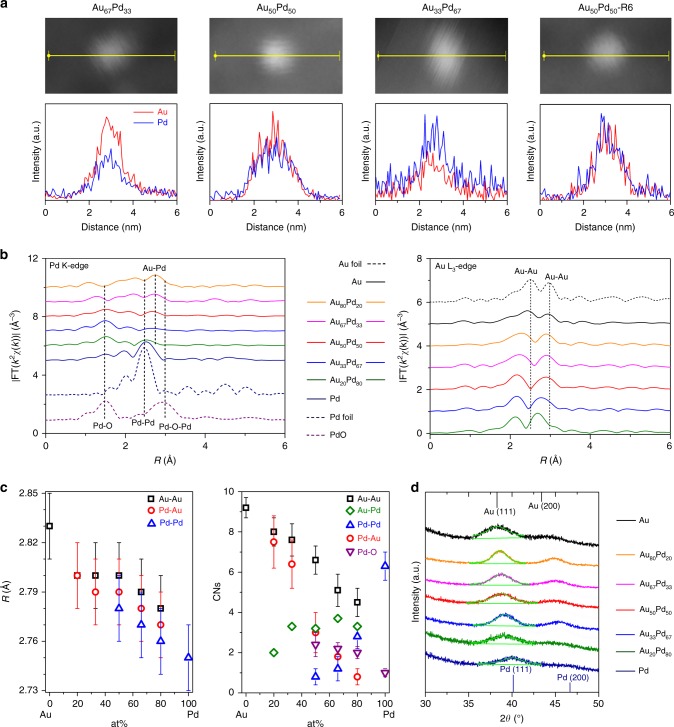
Microstructural characterization. **a** Representative line profiles of the EDX patterns of a single particle of the nanoalloys collected using a focused electron beam in the sub-nanometre range in the STEM mode. **b** *k*^3^-weighted and Fourier transformed magnitudes of the EXAFS spectra (|FT(*k*^2^χ(*k*))|) of the Pd K-edge and Au L_3_-edge of the nanoalloy and monometallic catalysts along with the reference metal foils and metal oxide. The Fourier transforms were not corrected for phase shifts. **c** Fourier transformed EXAFS fitting results of the bond distance (*R*) and the metal-metal coordination numbers (CNs) for the AuPd nanoalloys and monometallic nanoparticles. Error bars represent standard deviation. **d** WAXRD patterns with fine scans containing higher statistics collected near the diffraction peak at ~39°

**Fig. 2:**
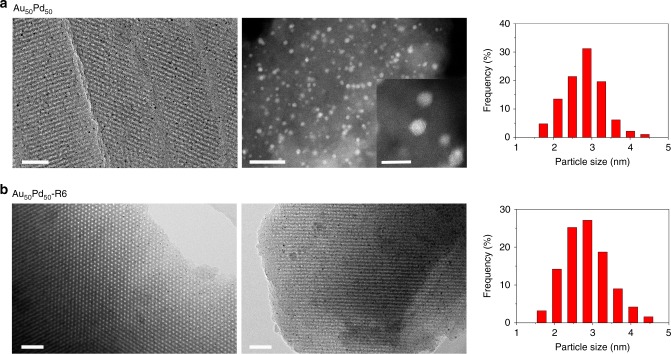
TEM micrographs. **a** Representative TEM image of the ultrathin sections (Left, the scale bar of 50 nm) and AC-STEM image (Middle, the scale bar of 20 nm) for the fresh Au_50_Pd_50_. Inset Middle figure is the AC-STEM image with a high magnification, and the scale bar is 5 nm. **b** TEM images of the recycled Au_50_Pd_50_-R6 after six catalytic runs viewed along the [001] and [110] directions. The scale bar is 50 nm. Particle size distributions (Right (**a**) and (**b**)) were determined with at least 200 nanoparticles

The representative Au and Pd line profiles of the energy-dispersive X-ray spectroscopy (EDX) spectra in the scanning transmission electron microscopy (STEM) mode for the Au_67_Pd_33_, Au_50_Pd_50_ and Au_33_Pd_67_ particles match well from the periphery to the centre. These results indicate that these bimetallic catalysts have uniform compositions throughout the particles, which quantitatively match with the synthesis ratios used within 10% (Fig. 1a, Table 1, Supplementary Figs. 1–4)^17^. Further analysis by EDX mapping in the TEM mode and X-ray photoelectron spectroscopy (XPS) measurements confirm that the Au:Pd ratios are close to the theoretical ones (Supplementary Figs. 5 and 6, Table 1). The TEM images of the bimetallic catalysts predominantly show well-distributed, isolated nanoparticles inside hexagonally arranged mesopores, and the nanoparticles have an average size of approximately 3.0 nm regardless of the metal compositions (Supplementary Fig. 7). The images of an ultrathin section of Au_50_Pd_50_ are quite similar to those of the above bulk catalyst, further confirming that the metal nanoparticles are intercalated into the mesopores instead of coating the outer surface of the carbon supports (Fig. 2). Aggregation of nanoparticles and their subsequent migration to the outer surface are hindered even at a high carbonization temperature of 600 °C. Interestingly, semi-exposure nanoparticles can be clearly observed. In addition, the spherical aberration corrected-STEM (AC-STEM) images for fresh Au_50_Pd_50_ show uniform nanoparticles with approximate size of 2.9 nm in large domains and negligible sub-nanometre metal clusters can be found (Fig. 2). Thus, the possibility for the activity contribution from the sub-nanometre clusters can be ruled out. Typical type-IV nitrogen sorption isotherms are observed for all studied catalysts, which are characteristic of open mesoporous solids with uniform pore sizes (Supplementary Fig. 8, Table 1). These phenomena indicate that metal nanoparticles do not show an obvious effect on the mesoporous structure.

**Table 1:** Structural and textural properties of the confined AuPd nanoalloy catalysts

Sample	Metal content^a^ (wt%)		Au:Pd (atomic ratio)				*D*_metal_^f^ (nm)	*a*_0_^g^ (nm)	*S*_BET_ (m^2^ g^−1^)	*V*_t_ (cm^3^ g^−1^)	*D*_p_ (nm)
	Au	Pd	Theo.^b^	XPS^c^	EDX-TEM^d^	EDX-STEM^e^					
Au	3.25	0	-	-	-	-	3.1	0.4062	359	0.24	3.6
Au_80_Pd_20_	2.90	0.39	4	4.18	3.88	n.p.^h^	3.2	0.4042	329	0.23	3.7
Au_67_Pd_33_	2.58	0.65	2	1.80	1.86	1.79	2.7	0.4022	334	0.23	3.6
Au_50_Pd_50_	2.12	1.17	1	0.90	0.92	1.10	2.9	0.4017	359	0.24	3.6
Au_33_Pd_67_	1.58	1.71	0.5	0.42	0.52	0.48	3.2	0.3991	336	0.23	3.6
Au_20_Pd_80_	1.03	2.25	0.25	0.21	0.22	n.p.	3.2	0.3980	315	0.22	3.6
Pd	0	3.31	-	-	-	-	2.8	0.3901	348	0.23	3.6
Au_50_Pd_50_-R6^i^	2.12	1.17	1	0.92	0.90	1.08	2.9	0.4017	292	0.18	3.4

For comparison, the properties for monometallic Au and Pd catalysts are also provided

^a^Theoretical Au and Pd contents

^b^Theoretical Au:Pd atomic ratio

^c^Au:Pd atomic ratio estimated from the XPS spectra

^d^Au:Pd atomic ratio estimated from the EDX pattern collected in TEM mode

^e^Au:Pd atomic ratio estimated from EDX with a focused electron beam in the sub-nanometre range in STEM mode

^f^Particle size estimated from the TEM images of the monometallic gold, monometallic palladium or nanoalloy Au_*x*_Pd_*y*_

^g^Lattice spacing of metal calculated from the wide-angle XRD pattern

^h^n.p.: not provided

^i^The Au_50_Pd_50_ catalyst after the sixth catalytic run

The *k*^3^-weighted and Fourier transformed extended X-ray absorption fine structure (EXAFS) spectra of the AuPd nanoparticles near the Pd K-edge and Au L_3_-edge are shown in Fig. 1b. Compared to the monometallic Pd catalyst, the AuPd bimetallic catalysts exhibit a dramatically reduced signal attributable to the Pd–Pd bond even with a low Au-to-Pd ratio. A new peak appears at a longer distance, which is primarily attributed to the Au-Pd bond^18^. The enhanced intensity of this peak with increasing Au content shows the gradual increase in the number of Au-Pd bonds in these nanocatalysts, which provides additional information regarding the formation of the alloy structure. Note that the Pd–O contribution is a major factor in Pd-rich catalysts and the lack of a major feature from Pd–O–Pd for Pd oxides essentially excludes the formation of oxides. When the Au-to-Pd ratio exceeds 2, the backscattering from Pd–Pd and Pd–O almost disappears. This phenomenon suggests that contiguous Pd clusters disappear with increased Au loading; isolated Pd domains form, and eventually each Pd atom is separated by an Au atom, forming isolated Pd single atoms at Pd-diluted nanoalloys^4,19,20^. Both core-shell and eutectic structures can thus be also excluded as the causes for this phenomenon^21^.

The typical spectral features due to the Au–Au first-shell backscattering contributions in the AuPd bimetallic catalysts shift even with a low Pd loading, and the peaks are broadened compared to how they appear in the spectra of monometallic gold, implying that the Au–Pd backscattering impacts these features^21^. The Au–Au bond distance decreases from 2.83 Å in pure gold to 2.80 Å in Au-rich alloys. A further lattice contraction is observed for Au-diluted nanoalloys, suggesting the presence of very small Au clusters^22^. The interatomic distances follow an order of Au–Au > Au–Pd(Pd–Au) > Pd–Pd, as shown in Fig. 1c. The Pd–Pd scattering distance in the AuPd nanoalloys increases with increasing gold content and is comparable to the interatomic distance for Au–Pd. These results suggest the presence of a well-defined Au–Pd phase^23^.

At the low percentage of Pd (<50%), the coordination number (CN) of Pd–Pd is zero and the CN of Pd–Au increases with reducing Pd percentage (Fig. 1c), suggesting an atomic dispersion of Pd in the Au matrix. When the percentage of Pd is 50%, the CN of Pd–Pd is about 1, where Pd exists dominantly as Pd dimer^24^. At the high loading of Pd (>50%), with increasing Pd percentage, the CN of Au–Au is not zero but decreases, while the CN of Pd–Pd increases exponentially; simultaneously, the CN of Pd–Au sharply decreases and the CN of Au–Pd is a relative constant. It suggests that the Au is not atomically distributed and prefers in the existence of small islands. The ratio of CNs for the Pd clusters (Pd–Pd and Pd–O coordination) to that of Pd–Au, is close to the Pd/Au ratio, which further implies that the alloy is random. The observation is in good agreement with the density functional theory (DFT) prediction by Nørskov and co-workers^25^. The segregation and mixing energy of Au–Pd (0.15 eV for Au-host and -0.14 eV for Pd-host alloys) suggest that Pd thermodynamically prefers a random-distribution, while Au prefers a segregation and exists as small islands in the alloy with the Au-host^10^.

The wide-angle X-ray diffraction (WAXRD) patterns show similar diffraction peaks for all catalysts regardless of their compositions (Supplementary Fig. 9). The symmetric diffraction peaks at approximately 39° imply the presence of a nanoalloy instead of a monometallic core-shell structure or macroscopic phase separation (Fig. 1d). This peak in the spectra of the bimetallic catalysts is clearly shifted compared to where it appears for monometallic Au and Pd. In fact, for the bimetallic catalysts, this peak is located between where it appears for either the monometallic Au or Pd species, which is the evidence for the formation of an alloy^26^.

### Electronic properties

**Fig. 3:**
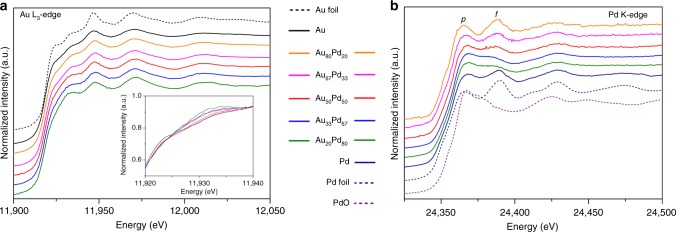
Electronic properties of the AuPd nanoalloys measured by XANES spectroscopy. **a** The XANES spectra of the Au L_3_-edge of monometallic Au, AuPd nanoalloys and reference Au foil. **b** The XANES spectra of the Pd K-edge of monometallic Pd, AuPd nanoalloys and reference Pd foil and PdO. The Pd XANES spectrum at the K-edge of the Pd foil exhibited a pronounced white line due to the unfilled Pd *d*-band, and the spectrum of PdO exhibited an even more obvious white line that shifted to a higher threshold energy because of the higher oxidation state of Pd in PdO. The hybridization-mediated 1 *s* → 4*d*, *dp* (*a*) absorption transition in the pre-edge region was unresolved. The near-edge spectrum showed two resonance peaks that are due to the 1 *s* → 5*p* (*p*) and 1 *s* → 4 *f* (*f*) transitions through hybridization. The shape of the second absorption edge reflected the extent of the 4*d*-5*p* hybridization. This feature was insignificant in the spectrum of the PdO reference

The X-ray absorption near-edge structure (XANES) spectrum of the Au L_3_-edge of the monometallic Au nanocatalyst shows a less intense white line than that of Au foil (Fig. 3)^27^. The further diminishment of white line intensity may result from either the presence of Pd in the enhanced filling of the Au *d*-band or the reduction in the Au–Au coordination number^22^. However, the white line intensity almost remains unchanged for all studied alloy catalysts. A substantial enhancement in the intensity of the post-edge feature is observed, when the Pd content increases, which is related to an increase in the number of available *d* states above the Fermi level^28^. This change can be attributed to the effect of the bimetallic distance, which causes the intra-atomic redistribution of charge or in other words, the decrease of the Au 5*d* electron states upon being alloyed with Pd^27,29^.

The hybridization-mediated 1 *s* → 4*d*, *dp* (*a*) absorption transition in the pre-edge region is not resolved in the spectra of monometallic Pd nanocatalyst and Pd foil. However, the first inflection point of the edges occurs on an expanded energy scale, implying the change in the oxidation state of the metal. The post-edge threshold is shifted to a slightly higher energy; this may be due to the presence of tiny Pd particles with a Pd metal *fcc* structure with very short range order and/or the coordination of Pd with a light O atom^30^. When the Pd nanoparticles are diluted with 20 and 33 at% Au, the white line energy position further shifts to higher energies in nearly identical fashion with the absorption edge as PdO, which is in agreement with the increase of Pd–O coordination. The 1 *s* → 5*p* (*p*) transition shifts to an even higher energy and the signal broadens. This shift can be interpreted as an electronic perturbation of the Pd atoms going from a Pd-like environment in the monometallic structure to the AuPd alloy phase. The Pd *d*-band becomes occupied^6^. A negative shift in the 1 *s* → 4 *f* (*f*) transition accompanied by a significant reduction in intensity is detected, which reflects the increase in the atomic distance and 4*d*-5*p* hybridization^31,32^. Similar phenomena have been observed for local distortions of the Pd lattice by interstitial C^31^ and AuPd^33^ bimetallic nanoparticles. The above shifts are poorly resolved in the spectra of Au_50_Pd_50_, implying that the 4*d* band is filled. With further dilution with Au atoms, both the white line and thresholds of the Au_*x*_Pd_*y*_ catalysts shift towards those observed for Pd foil, indicating a change in the orbital character of the screening charge^34^. The Au_80_Pd_20_ sample shows the most intense metallic Pd features in which the Pd–Pd and Pd–O bonds cannot be detected. Isolated Pd atoms in the Au aggregates can be demonstrated.

**Fig. 4:**
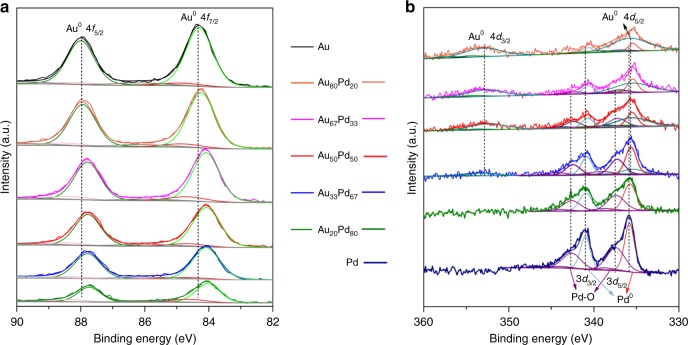
Surface compositions and electronic properties. **a** XPS spectra of the 4 *f* level of Au. **b** XPS spectra of the 3*d* level of Pd. The Au and Pd binding energies were fitted by peak fitting techniques. Metallic Au, metallic Pd and Pd–O, which were the dominant contributors to each spectrum, were discussed. A distinct overlap could be observed between the Pd 3*d*_5/2_ and the Au 4*d*_5/2_ components for the bimetallic catalysts. The Au 4*d*_5/2_ intensity was calculated from the well-resolved Au 4*d*_3/2_ intensity, and this value was subtracted from the above overlapped peak to determine the Pd 3*d*_5/2_ intensity. The resulting value was used to calculate the Au:Pd ratio

The typical doublets for the Au 4 *f* XPS spectra can be deconvoluted, and the major peaks for Au^0^ (85.7%) in all Au-containing catalysts can be fitted (Fig. 4). Compared to the monometallic Au catalyst, the Au 4 *f* core levels of the AuPd nanoalloys show a gradual negative shift towards a lower binding energy as the Pd loading increases. The doublets for the Pd 3*d* core bands of the monometallic Pd catalyst can be deconvoluted into three phases; the major peak fitted can be assigned to metallic Pd, a minor peak can be attributed to Pd–O coordination^35^, which comes from the carbonyl, carboxyl, hydroxyl groups in carbon carrier or oxygen in air, and an extremely weak peak can be attributed to O–Pd–O coordination (not discussed here). As the Pd loading decreases, a gradual negative shift is observed for AuPd nanoalloys due to the Au–Pd alloy interaction. A relatively large shift is observed for the Pd-diluted alloys. Interestingly, the Au_80_Pd_20_ nanoparticles show a less intense peak from the Pd–O bond. This result indicates that diluted Pd atoms in an Au host are much more stable in air. The negative shifts of both the Au 4 *f* and Pd 3*d* core level bands in bimetallic species compared to the corresponding monometallic species have been also reported for bulk AuPd alloys^36^, in contrast to Cu–Pd bulk alloys^37^, where the Pd shifts are positive and the Cu negative. Models in which the redistribution of occupied valence shells is caused by a difference in the hybridization of Pd and Au appear quite reasonable^38,39^. The *d* band is active in alloying; the non-*d*-charge flows onto an Au site is largely compensated by accompanying Au *d* charge depletion; the net charge flow is small. The detailed discussion is shown in the following part on combination with the XAFS results.

### Kinetics analysis and DFT calculations

**Fig. 5:**
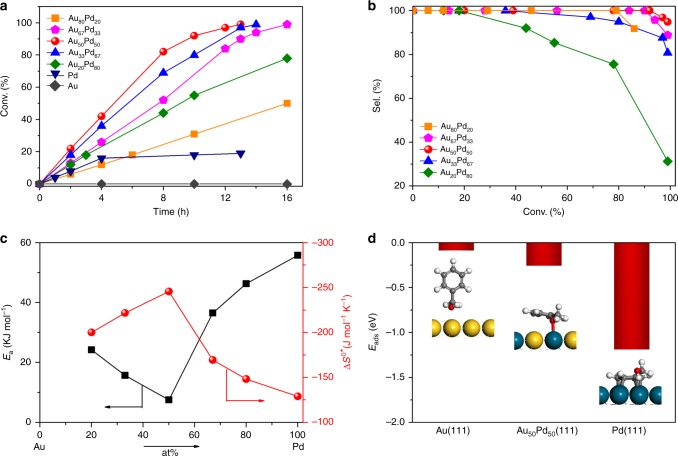
Kinetics analysis and DFT calculations. **a** Benzyl alcohol conversion (Conv.) as a function of time with the following reaction conditions over monometallic Au, Pd and AuPd alloy nanocatalysts. **b** Compile of the selectivity (Sel.) to benzyl aldehyde as a function of benzyl alcohol conversion over AuPd alloy nanocatalysts. **c** Relationship between the activation energy (*E*_a_) and entropy of activation (Δ*S*^0*^) for the oxidation of benzyl alcohol to benzaldehyde on the AuPd alloys as a function of concentration. **d** The most favourite configuration and the adsorption energy (*E*_ads_) of benzyl alcohol over Au(111), Pd(111) and Au_50_Pd_50_(111) by DFT calculations. The reaction conditions were: 32 mg of catalyst; 5.0 mmol of substrate; 10 mL of water; 90 °C; and in the presence of oxygen by an O_2_ balloon under atmospheric pressure

The aerobic oxidation of primary alcohols in an aqueous solution in the absence of base is used as a model reaction here because this is a strong-adsorbate-involved reaction and Pd-involving sites are more active than the Au case on a carbon support^40^. The neat Pd catalyst results in a low conversion and fast deactivation. The complete oxidation of benzyl alcohol (BA) to CO_2_ and the depletion of O_2_ can be excluded. Therefore, the switching-off for the neat Pd catalyst is possibly due to the strong adsorption of produced aldehyde and acid on the surface. Our results have also shown the content of products generated in the Au-catalysed oxidation of the selected primary alcohols is below the limit of detection, similar to the gold sols loaded on activated carbon catalyst^41^. It should be mentioned that the studied carbon support serves as a less-active support^42^, and the gold nanoparticles are not able to extract the hydride from the alcoholic function. Once the base is present, the alcoholate can be formed in solution, and the oxidation activity can be significantly improved^41,43–45^. While alloying a small amount of Pd (Au:Pd = 4) with the Au NPs yields oxidative products, e.g. the BA conversion approaches 12% at 4 h (Fig. 5).

The leaching of dissolved metal ions is firstly excluded. A solid containing an unprotected thiol was used to poison the possible soluble metallic species in BA oxidation^46^. Results show no significant differences in the turn-over frequency (TOF) values of any of the AuPd nanoalloys in the presence/absence of mercapto-functionalized ordered mesoporous silica (SH-SBA-15). It confirms the negligible Pd leaching into solutions (Supplementary Fig. 10). After six catalytic runs using Au_50_Pd_50_, both the BA conversions in the initial phase of the reaction and at the end of the reaction show no substantial changes, and only a slight increase is observed for the initial conversion (Supplementary Fig. 11). The collected solution after each run shows undetected metal ions. The TEM images of the recycled Au_50_Pd_50_ nanocatalyst are quite similar to those of the fresh catalyst, and show invisible nanoparticle aggregation (Fig. 2). The EDX mapping spectra give the Au and Pd concentration of 2.18 and 1.32 wt%, respectively, similar to that determined for the fresh catalyst (2.15 and 1.27 wt%, respectively), further excluding the leaching of metal into solutions. The estimated Au:Pd ratios based on the XPS analysis, XRD patterns or the representative EDX line profiles in the STEM mode (Fig. 1, Supplementary Table 1 and Fig. 12) are almost the same as that in the fresh catalyst, indicating the surface segregation of Pd or Au can be excluded. The pore volumes slightly decrease, possibly because organic residue is deposited inside the mesopores (Table 1 and Supplementary Fig. 8). These results indicate the mesostructure and heterogeneous AuPd alloy nanocatalysts are stable, and negligible leaching occurs even during repeated reactions involving strong adsorbates. The leaching-resistance feature during reactions even involving S-containing substances has also been found in the nanoparticles inside mesoporous carbon^43,47^. This can guarantee the investigation of surface catalysis and almost unchanged surface Pd concentrations after reaction.

Decreasing the Au/Pd ratios to 2 and 1 results in increments of the BA conversions to 26 and 42% at 4 h, respectively. However, the BA conversion does not improve on further increasing the Pd concentration. The reduction of BA conversion to approximate 21% is observed for Au_20_Pd_80_. For a rigorous comparison, the selectivity was compared restraining the similar BA conversions. Only benzaldehyde is detected in the initial period. With an increase in the conversion, the only by-product of benzoic acid is produced. Other byproducts such as toluene and benzyl benzoate are undetected, analogous to the results over supported AuPd nanocatalysts reported by Prati and co-workers^41^. The formation of the byproducts is very sensitive to the reaction conditions and catalyst used. For example, toluene is one of the main byproducts in the solvent-free BA oxidation at a relatively high temperature (>120 °C) possibly via the disproportionation of BA^1^, but is seldom detected in the water-mediated reaction^41^. The concentration variations of components with time conform to the regularity of the consecutive reaction. The transformation to the by-product is also highly related to the Pd concentration in the nanoalloy catalysts. We observed that the Au_50_Pd_50_ and Au_67_Pd_33_ catalysts also exhibit the highest selectivity under a high conversion (above 90%). The decrease in the Pd loading slightly decreases the selectivity. The over oxidation occurs at a conversion of ~80% for Au_80_Pd_20_. On contrary, the overloading of Pd in nanoalloys results in a dramatic reduction of selectivity. The by-product of benzoic acid can be detected with a BA conversion of 69 and 44% over Au_33_Pd_67_ and Au_20_Pd_80_, respectively.

Preliminary experiments indicated the absence of diffusion limitations and mass transfer effect under present reaction conditions (Methods, Supplementary Fig. 13). The approximate constants of reaction rates along with the given BA concentration ranges would suggest the reaction orders for BA is Pseudo zero order (Supplementary Fig. 14). In addition, the studied catalysts show almost Pseudo zero-order reaction kinetics during the initial period of the reaction. The reaction constant (*k*_Pd_) can be deduced. Arrhenius plots are presented in Supplementary Fig. 15, where the apparent activation energies (*E*_a_) and the pre-exponential factors (*A*) were estimated. The apparent entropies changes (Δ*S*^0*^) for the nanoalloy catalysts were also estimated based on transition state theory. The activation energies follow a reverse volcano-shaped curve while Δ*S*^0*^ follows a volcano-like curve as a function of the Pd percentage as shown in Fig. 5c, and the maximum Δ*S*^0*^ and minimum *E*_a_ are observed for the 50 at% Au catalyst. A linear relationship between *E*_a_ and Δ*S*^0*^ has been observed (Supplementary Fig. 16), which is called the compensation effect, due to the changed bonds between the surface atoms and the adsorbates^48^.

The estimated turn-over frequency on the basis of exposed Pd-involving sites (TOF_Pd_) was taken to eliminate the effect of Au dilution (Supplementary Fig. 16). Volcano-type behavior was observed for TOF_Pd_ when going from pure Au to pure Pd (Supplementary Fig. 17), and the maximum TOF was achieved with Au_67_Pd_33_ and Au_50_Pd_50_ showing ~9 times higher than the neat Pd catalyst, which is characteristic of structure-sensitive reactions^49^. The TOF_Pd_ values for the Pd-diluted catalysts are much higher than the case of Pd-rich catalysts. Similar results have been observed for selective oxidation of primary alcohols, including 4-methylbenzyl alcohol, 4-anisalcohol and phenethyl alcohol.

Periodic DFT calculations were performed on the adsorption of BA on Au_50_Pd_50_(111), Pd(111) and Au(111) surfaces at low coverage (Fig. 5d, Supplementary Table 2 and Figs. 18–20). The most favourite adsorption configuration for BA on Pd(111) is that the phenyl of BA parallels and strongly interacts with the Pd(111) surface, while hydroxyl group tilts away from the surface. Different studies agree with that BA oxidation proceeds through a benzyl alkoxide (PhCH_2_O-) intermediate to adsorbed benzaldehyde^44,50–52^. The bond scission sequence is O–H followed by C^*α*^-H. The surface adsorbed HO* or HOO* species promotes the elimination of C^*α*^-H, which is considered as the rate-determining step. Accordingly, the weak activation of the O–H bond and the coverage of the phenyl functions which could poison the Pd surface are the possible reasons for the inhibition of dehydrogenation and in turn activity. In addition, this process is different with the methane oxidation using H_2_O_2_ as the oxidant, in which the hydroxyl radicals (HO·) from the homolytic cleavage of the HO-OH bond can oxidize methanol to HCOOH and CO_2_^53,54^. On contrary, the chemical bonding between the Au(111) surface and the functional group of hydroxyl and phenyl group is negligible. Therefore, the Au surface is not able to extract the hydride from the alcoholic function when supported on less-active porous carbons and behaves inert in BA oxidation. The chemisorption of BA on the AuPd(111) surface through hydroxyl group shows an evidently enhanced adsorption energy compared to the parallel adsorption via aromatic ring. As a result, it directly activates the OH group in BA, and facilitates the further dehydrogenation.

## Discussion

An adsorbate mediated reduction is responsible for the formation of AuPd nanoalloys with tuneable hybridization and heterometallic bond distances. We monitored the preparation of the catalysts. The ultraviolet-visible (UV-Vis) diffuse reflectance spectroscopy shows that ligand exchange is converting Au–Cl and Pd–Cl coordination to Au–S and Pd–S species in the mother solutions (Supplementary Fig. 21) The temperature-programmed decomposition-mass spectrometry (TPDe-MS) measurements (Supplementary Fig. 22) present that the decomposition of resins can occur at elevated temperatures, and the sulfur is released in the form of SO_2_ which provides an opportunity for homogeneous mixing of Au and Pd species at atomic level to generate the AuPd nanoalloys. Simultaneously, CO gas is released to create the reducing atmosphere for the reduction of the metal precursors. The breakthrough of the Au–S and Pd–S bonds at a temperature above 220 °C is accompanied with the reduction of metals and the condensation of the silicate-carbonaceous framework. The particles are confined by the rigid silica-carbon hybrid framework, homogenously disperse in the matrix, and show high stability even under a reductive atmosphere at 600 °C. The almost complete elimination of sulfur is observed for all catalysts based on EDX and XPS measurements, which could guarantee the excluding of the additional ligand effects. On the other hand, the gases (CO and SO_2_) also facilitate the formation of surface enrichment of Pd rather than thermodynamically stable Au-segregated surface. This is mainly attributed to the preferential adsorption of CO on the Pd atoms compared to Au because of the much stronger binding of Pd with CO. Mariscal et al.^55^ reported that the pre-adsorption of CO can easily cause a structural transformation from a Pd core and an Au shell to an Au core and a Pd shell. All of these analyses indicate that our developed reduction method can breakthrough the thermodynamic limitation to generate the AuPd nanoalloys against thermodynamically favourable Au rich surfaces by means of the unique adsorbate induction.

The unambiguously determined alloy structure at atomic level can be used to determine the *d* charge at Pd site as a function of alloy composition^56^. The binding energy shift, Δ*E*_B_, is related to the changes in the one-electron energy, the change in the work function, and the final-state relaxation of a core hole between the metal and the alloy. Accordingly, non-*d*- and *d*-charge transfer onto the atom site can be estimated based on the XPS and XANES results (Supplementary Table 3)^57^. For the Pd-rich alloy, the amount of the charge transfer is in general very small upon alloying, almost less than 0.1 electrons in alloys, and the total charge transfer at Pd site is smaller than the change of *d* electron number. The Au atoms appear to gain 0.1–0.2 *sp* electrons, compensated by a loss of about the same number of *d* electrons^36^. The intra-atomic charge transfer is more important than the interatomic charge transfer between Pd and Au atoms. The maximum *d*-charge gain at approximate 50 at% Pd alloy composition with Pd dimer structure results in a pronounced change in the available electronic energy states. With a further reduction in the Pd concentration, where the isolated Pd single atoms are formed, the net charge between Au and Pd dramatically enhances. But the *d*-electron number reduces to some extent. The 5*sp* electron of the surface Pd atoms may lose some *d*-character due to the isolated Pd atoms and the coordination with Au, which have increasing Pd–Pd bond distances. The *d*-*d* separation would narrow the 4*d* band of the surface Pd sites effectively pushing the top of this band below the Fermi level. This trend in the *d*-band filling is consistent with that of the AuPd bulk alloy^58^. Theoretical calculations support this conclusion^34,59^. The 33 at% Pd alloy adapting a single atom structure exhibits a similar *d*-charge gain with the 50 at% Pd alloy. While the 20 at% Pd alloy shows a decreased *d* electron numbers. The *d* charge of single atom decreases with increasing its coordination number with Au. Therefore, the single atom and dimer of Pd distributed in the inert Au matrix can be identified as the active sites^24,60–63^. In addition, the catalytic activity and selectivity depends on the coordination of Pd to Au atom or more likely its electronic properties in nanoalloy.

**Fig. 6:**
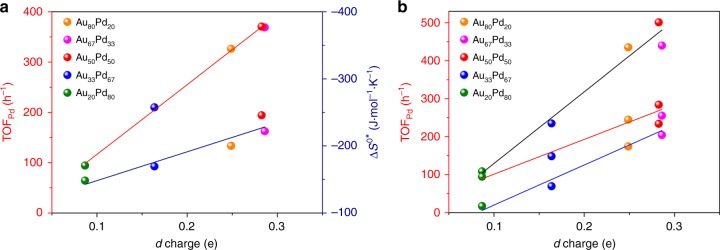
Descriptor. **a** Relationship between the *d-*charge gain at Pd site and TOF_Pd_ (red line) of the AuPd alloys, and the entropy of activation for the oxidation of benzyl alcohol to benzaldehyde (Δ*S*^0*^, navy line). **b** Comparison of the TOF_Pd_ values of the nanocatalysts along with the compositions of the AuPd nanoalloys in the oxidations of 4-methylbenzyl alcohol (black line), 4-phenethyl alcohol (red line) and anisalcohol (blue line)

The measured apparent entropy change is the difference between the entropy of the transition state and the reactant. The value reflects the freedom loss of the species due to the adsorption on the surface, more precisely the adsorption strength^64^. The activation entropy change would be correlated with the density of states close to the Fermi level of the transition metals, which show great effect on the bound state for the absorbed intermediate^65^. We therefore attempt to relate the *d*-charge gain to the activation entropy, Δ*S*^0*^ (Fig. 6). The entropy change indeed follows a linear relationship with the *d* charge at the Pd sites. This result indicates that the Pd charge of the Group VIII-IB nanoalloy is fundamental to the activation entropy associated with the adsorbate binding strength in the surface reaction. The increase in the reaction rate achieved with the nanoalloy (isolated or dimer of Pd in Au clusters) would be expected based on the increase in Δ*S*^0*^, which is related to the stronger adsorption strength^66^. The bound state for PhCH_2_O- over Au_50_Pd_50_ is therefore much favourable for catalytic dehydrogenation. Accordingly, the *d* charge at surface Pd site can serve as a descriptor, which can correlate well with the activities (Fig. 6). The linear relationship can also be followed for other primary alcohols oxidation including 4-methylbenzyl alcohol, 4-anisalcohol and phenethyl alcohol.

The maximum in the *d*-charge gain is found on Pd single atom and dimer with about 33–50 at% Pd. The slight increase of the TOF_Pd_ value in the repeated use may also reflect the change of Pd charge originated from the adsorbed oxygen-containing compounds over the used catalyst. Simultaneously, the adsorption configuration over Au_50_Pd_50_, Au_67_Pd_33_ and Au_80_Pd_20_ with Pd dimer and single atoms separated by Au atoms may hinder the disproportionation of two BA molecules to give benzaldehyde and toluene, and therefore enhances the selectivity at high conversions compared to literatures^1^. In addition, the improved adsorption strength of BA may inhibit the adsorption of benzaldehyde and the consecutive oxidation to benzoic acid. The selectivity to benzaldehyde is significantly enhanced at high conversions. It has been reported that the absolute thermoelectric power, *S*, of AuPd alloys is highly sensitive to the presence of unoccupied *d*-band states^58^. As expected, the trends are similar for the catalytic activity, the entropy change and *S* with alloy compositions (Supplementary Fig. 23).

In summary, we have identified the *d* charge at the Pd sites as a descriptor to predict entropy changes and activities in the oxidation of alcohols. The AuPd solid-solution nanoalloys confined by ordered mesoporous carbon with the detailed chemical, geometric and electronic structures at atomic level have been synthesized by the adsorbate mediated reduction and used to unambiguously determine the changing of the metal ligands of the surface palladium atoms. The maximum *d*-charge gain is obtained on the Pd single atom and dimer at approximate 33–50 at% Pd in Au–Pd nanoalloy, which results in a pronounced improvement in the adsorption strength of primary alcohols, and therefore apparently higher aerobic oxidation activity and selectivity of primary alcohols under base-free conditions compared to those of an inert carbon-supported monometallic gold or low-activity palladium catalyst. The experimentally measured *d* charge on the  transition metal sites, together with the DFT calculations on the nature of the adsorbing molecule and the demands which it makes, paves the way for the rational design and synthesis of bimetallic catalysts with tailored activities for wide catalytic applications, and the identification and tracking of key steps involved in the catalytic reactions.

## Methods

### Synthesis of AuPd alloys inside ordered mesoporous carbon

The synthesis involved the assembly of carbon precursors of low-molecular-weight phenolic resins, a silica source (tetraethylorthosilicate, TEOS, Acros Chemical Inc.), a gold source (chloroauric acid tetrahydrate, HAuCl_4_·4H_2_O, > 99.9 wt%, Sigma-Aldrich), a palladium source (PdCl_2_, palladium chloride solution, 5 wt% in 10 wt% HCl, Sigma-Aldrich Co.), a coordinating reagent (3-mercaptopropyltrimethoxysilane, MPTMS, 85 wt%, Acros Chemical Inc.), and a structure directing agent (poly(ethylene oxide)-b-poly(propylene oxide)-b-poly(ethylene oxide) triblock copolymer F127, EO_106_PO_70_EO_106_, *M*_W_ = 12,600 g mol^−1^, Acros Chemical Inc.) followed by an adsorbate mediated reduction. Typically, 4.16 g of TEOS and 3.94 g of MPTMS were pre-hydrolysed in the presence of 4.0 g of HCl (0.2 M) and 20.0 g of ethanol for 30 min to obtain solution A. To a solution containing 6.4 g of Pluronic F127, 16.4 mL of HAuCl_4_ solution (gold concentration in ethanol: 48.5 mmol L^−1^), 7.2 mL of palladium chloride solution (palladium concentration in ethanol: 56.4 mmol L^−1^), 4.0 g of preformed phenolic resins (Supplementary Methods) and 20.0 g of ethanol, solution A was added. The solution was stirred for 1 h at 40 °C, and then the mixture was transferred into evaporating dishes. The solvent evaporation was carried out in a hood at ambient temperature for 6 h, and the resins were polymerized in an oven at 100 °C for 24 h. The as-prepared materials were obtained by scraping the films from the dishes and refluxing the material in sulfuric acid with mechanical stirring (1.0 g of solid per 100 mL of 50 wt% sulfuric acid) at 90 °C for 24 h to remove the triblock copolymer and pre-polymerize the resins. The resulting material was removed by filtration, washed with distilled water, and dried at 80 °C under vacuum overnight. The as-prepared powders were transferred into a tubular furnace for reduction of the bimetallic material. The following heating programme was adopted. The temperature was increased from room temperature to 350 °C at a rate of 1 °C min^−1^, increased to 600 °C at a rate of 5 °C min^−1^, and then held for 3 h. After cooling to room temperature under protect by N_2_ atmosphere, the black powders were treated at 180 °C in an oven in air. Then, the obtained composite catalysts were named Au_*x*_Pd_*y*_, wherein *x* and *y* represent the Au/(Au + Pd) and Pd/(Au + Pd) values in molar percentages in the mother liquor, respectively. The loadings of Au and Pd in the nanocatalysts are estimated by the EDX mapping analysis in the TEM mode. It reveals that the total amount of metals is ~2.8–3.6 wt% for all catalysts and both the amounts and the Au:Pd ratios are close to the theoretical values (Table 1, Supplementary Fig. 5).

For comparison, monometallic Au and Pd and metal-free carrier (ordered mesoporous carbonaceous carrier (OMCC)) were synthesized according to the described processes with the indicated total metal molar contents or in the absence of metal. The specific surface area (*S*_BET_), pore size (*D*_p_), and pore volume (*V*_t_) is 417 m^2^ g^−1^, 6.6 nm and 0.49 cm^3^ g^−1^, respectively. 

### Characterization of the materials

The XRD measurements were taken using a Rigaku Dmax-3C diffractometer (Cu K_α_ radiation). Structural investigations of the catalysts were performed on a JEM 2100 microscope operated at 200 kV and an FEI Tecnai G2 F30 microscope equipped with a high-angle annular dark-field scanning transmission electron microscopy (HAADF-STEM) detector in TEM and STEM modes. The elemental analyses of the spots, lines and areas selected in the HAADF-STEM images were performed using a Philips EDX instrument. The EDX spectra were obtained in STEM mode with a focused electron beam in the sub-nanometre range. The EDX spectra were collected from ~20 nanoparticles in different areas. The AC-STEM images were performed using a JEM-ARM 200 F microscope operated at 200 kV. An ultrathin sectioning technique was applied to prepare ultrathin sections by embedding the sample in LR White acrylic resin (hard) and sectioning on an ultramicrotome. The thin sections (∼80 nm) were supported on 300-mesh copper grids. N_2_ physisorption isotherms were recorded at 77 K with a Micromeritics TriStar II 3020 analyser using the BET method to estimate the specific surface areas (*S*_BET_) and the Barrett-Joyner-Halenda (BJH) model to derive the pore volumes and pore size distributions. The real-time UV-Vis spectra were obtained using a UV-Vis spectrophotometer (Varian Cary 100). A Varian VISTA-MPX inductively coupled plasma-atomic emission spectrometry (ICP-AES) instrument was used to determine the gold and palladium loadings. The XPS measurements were recorded on a Perkin-Elmer PHI 5000CESCA system. The samples were evacuated in a Load lock chamber and then transferred to the analysis chamber (10^−9^ mbar). The C 1 *s* peak was adopted as an internal standard for the determination of the peak positions and was corrected to 284.6 eV. The Au and Pd binding energies were fitted by peak fitting techniques. Thermal analysis (TG-DTA) measurements were performed on a Mettler-Toledo TG/SDTA 851e apparatus. Samples were heated from 25 °C to 800 °C at a rate of 10 °C min^−1^ with nitrogen flowing at a rate of 50 mL min^−1^. Upon heat treatment of the as-prepared material, the TG curves revealed that approximately 48 wt% residue solid remained (Supplementary Fig. 24). The theoretical metal contents were thus roughly estimated by the adding amounts of metal assuming none of the mass lost during thermal reduction was from the metals (Table 1). TPDe measurements were made on a Micromeritics Auto Chem II 2920 instrument coupled to a well-calibrated Microvision 2 quadrupole mass spectrometer (MKS Instruments Cirrus II). Prior to the TPDe measurements, the sample (ca. 50 mg) was purged at 50 °C for 1 h with flowing dry He (40 mL min^−1^, > 99.999%). The purging temperature was then increased to 800 °C at a rate of 10 °C min^−1^, and dry He continued to flow at a rate of 40 mL min^−1^. Fragments with mass-to-charge (*m*/*z*) ratios of 16, 18, 28, 44, and 64 were observed^67^.

X-ray absorption spectra of the Au L_3_-edge and Pd K-edge were conducted at the BL14W1 of the Shanghai Synchrotron Radiation Facility (SSRF). The samples were pressed into pellets and measured in fluorescence mode. A double-crystal Si(111) monochromator was used for energy selection at both the Au L_3_-edge (11,919 eV) and Pd K-edge (24,350 keV). The Au and Pd EXAFS data of the same sample were processed simultaneously in **R**-space using a software package for fitting of EXAFS data by fast Fourier inverse transform (IFEFFIT). The CN of Pd–Au (CN_Pd–Au_) and Au–Pd (CN_Au–Pd_) were fixed according to the equation (1) in the literature^68^:

1 $${\mathrm{CN}}_{{\mathrm{Pd - Au}}} = \frac{{\chi _{{\mathrm{Au}}}}}{{\chi _{{\mathrm{Pd}}}}}{\mathrm{CN}}_{{\mathrm{Au - Pd}}}$$

Where *χ*_Au_/*χ*_Pd_ is the atomic ratio of Au to Pd. The heterometallic bond distances and corresponding Debye–Waller factors were also constrained to be the same.

The binding energy shift Δ*E*_B_ of the core level between pure metal and the alloy measured relative to the Fermi level in XPS experiments was related to changes of the Hartree–Fock one-electron energy of the core level, the change in the work function, and the final-state relaxation of a core hole between the metal and the alloy^57^. The calculation and results were shown in Supplementary Table 3.

All periodic spin-polarized DFT calculations were carried out using the Vienna Ab-initio Simulation Package (Supplementary Table 2).

### Catalytic tests

The oxidation reactions were carried out in three-neck, round-bottom flasks. In a typical reaction, to a mixture of 5.0 mmol of BA and 10 mL of deionized water, 32 mg of powdered Au_50_Pd_50_ catalyst was added. The suspension was maintained in the presence of oxygen by an O_2_ balloon under atmospheric pressure, the stirring speed was fixed at 800 rpm and the temperature was kept at 90 °C. Preliminary experiments performed by ranging stirring speeds from 400 to 1000 rpm showed that the selected speed of 800 rpm enabled the reaction to perform without diffusion limitations. The effect of mass transfer effect on the reaction rate was studied by varying the surface concentrations of metal but with similar dispersion, known as the Madon–Boudart (MB) test. Two additional samples with different total metal contents (1.28 and 1.84 wt%) were synthesized. These two samples showed similar XRD patterns, N_2_ sorption isotherm curves and TEM images with the studied Au_50_Pd_50_ catalyst (Supplementary Figs. 25–27). A linear relationship indicated the absence of mass transfer effect under present reaction conditions (Supplementary Fig. 13). The easy accessible of alloy nanoparticles intercalated into framework was related to the large-enough mesopores. Hot filtration was used at different time intervals to determine the BA conversion and metal leaching. Both the solid catalyst and aqueous mother liquor were extracted with ethyl acetate. The organic phases were combined and analysed to determine the conversion and yield by gas chromatography-mass spectrometry (GC-MS, 7890B-5977) or GC (Agilent 7890B) using a DB-Wax column. The solid catalyst was recovered for reuse by washing with water and drying at 80 °C overnight under vacuum. The gas phase was analysed to determine the concentrations of O_2_, CO and CO_2_ by GC (7890 A) using a porapak Q column. The analysis was repeated at least three times with ± 5% experimental errors (standard deviation). The carbon balance for all tests was above 95%. The oxidation was also carried out with 5.0 mmol of 4-methylbenzyl alcohol, 4-anisealcohol and phenylethyl alcohol.

The kinetic rate equation was deduced by considering that BA adsorbed on Pd and the mechanism for adsorbed alcohol dehydrogenation involved two consecutive dehydrogenation steps. A global kinetics constant *k* was employed in the kinetics rate equation due to the fact that the first dehydrogenation of the bond scission of O–H and the second dehydrogenation of the bond scission of C^*α*^-H could not be measured independently. The TOF, activation energy (*E*_a_), and entropy of activation (Δ*S*^0*^) were determined (Supplementary Fig. 16). The TOF value for each catalyst was calculated on the basis of the number of exposed palladium atoms, which was estimated on the similarities of the truncated octahedron shape of the nanoparticles (as evidenced in the AC-STEM image) and the homogeneous distribution in the alloy (Supplementary Fig. 16).

The recycling tests were carried out as following. Each time the catalyst was recycled, the oxidation reaction was performed under the same conditions. To ensure the same amount of catalyst was present during each cycle, multiple parallel tests were performed. The catalyst after the sixth run was referred to as Au_50_Pd_50_-R6. The aqueous solutions after each run were collected and combined to determine the metal leaching.

Solid mercapto-functionalized ordered mesoporous silica (SH-SBA-15, Supplementary Fig. 28) was used to trap the soluble metal species that had leached into solution. Typically, 49 mg of SH-SBA-15 was added to the reaction flask prior to the addition of the reaction solution to give a molar ratio of SH:Pd = 35:1.

## Supplementary information

Supplementary Information

Peer Review

## Data Availability

The data that support the findings of this study are available from the corresponding author upon reasonable request.
