# Supplementary material for: Optimising surface d charge of AuPd nanoalloy catalysts for enhanced catalytic activity
Source: Nat Commun. 2019 Mar 29;10:1428. doi: 10.1038/s41467-019-09421-5 (PMC6441046; doi:10.1038/s41467-019-09421-5)
Supplement: Supplementary file 1 — Supplementary Information [file 41467_2019_9421_MOESM1_ESM.pdf]

Supplementary Information

Optimising surface *d* charge of AuPd nanoalloy catalysts for enhanced catalytic activity

Zhu et al.

Optimising surface *d* charge of AuPd nanoalloy catalysts for enhanced catalytic activity

Xiaojuan Zhu[1†], Qishui Guo[1†], Yafei Sun[1†], Shangjun Chen[1], Jianqiang Wang[2], Mengmeng Wu[1], Wenzhao Fu[3], Yanqiang Tang[3], Xuezhi Duan[3], De Chen[4] and Ying Wan[1*]

[1]Key Laboratory of Resource Chemistry of Ministry of Education, Shanghai Key Laboratory of Rare Earth Functional Materials, and Department of Chemistry, Shanghai Normal University, Shanghai, P. R. China.

[2]Shanghai Synchrotron Radiation Facility, Shanghai Institute of Applied Physics, Chinese Academy of Sciences, Shanghai, P. R. China.

[3]State Key Laboratory of Chemical Engineering, East China University of Science and Technology, Shanghai, P. R. China.

[4]Department of Chemical Engineering, Norwegian University of Science and Technology, Trondheim, Norway.

[†]These authors contributed equally to this work.

[*]Correspondence should be addressed to Y.W. (email: ywan@shnu.edu.cn)

Contents

Supplementary Figures……………………………………………………………………………….................4
Supplementary Tables……………………………………………………………………………………..34
Supplementary Methods…………………………………………………………………………………...38
Supplementary References………………………………………………………………………………....39

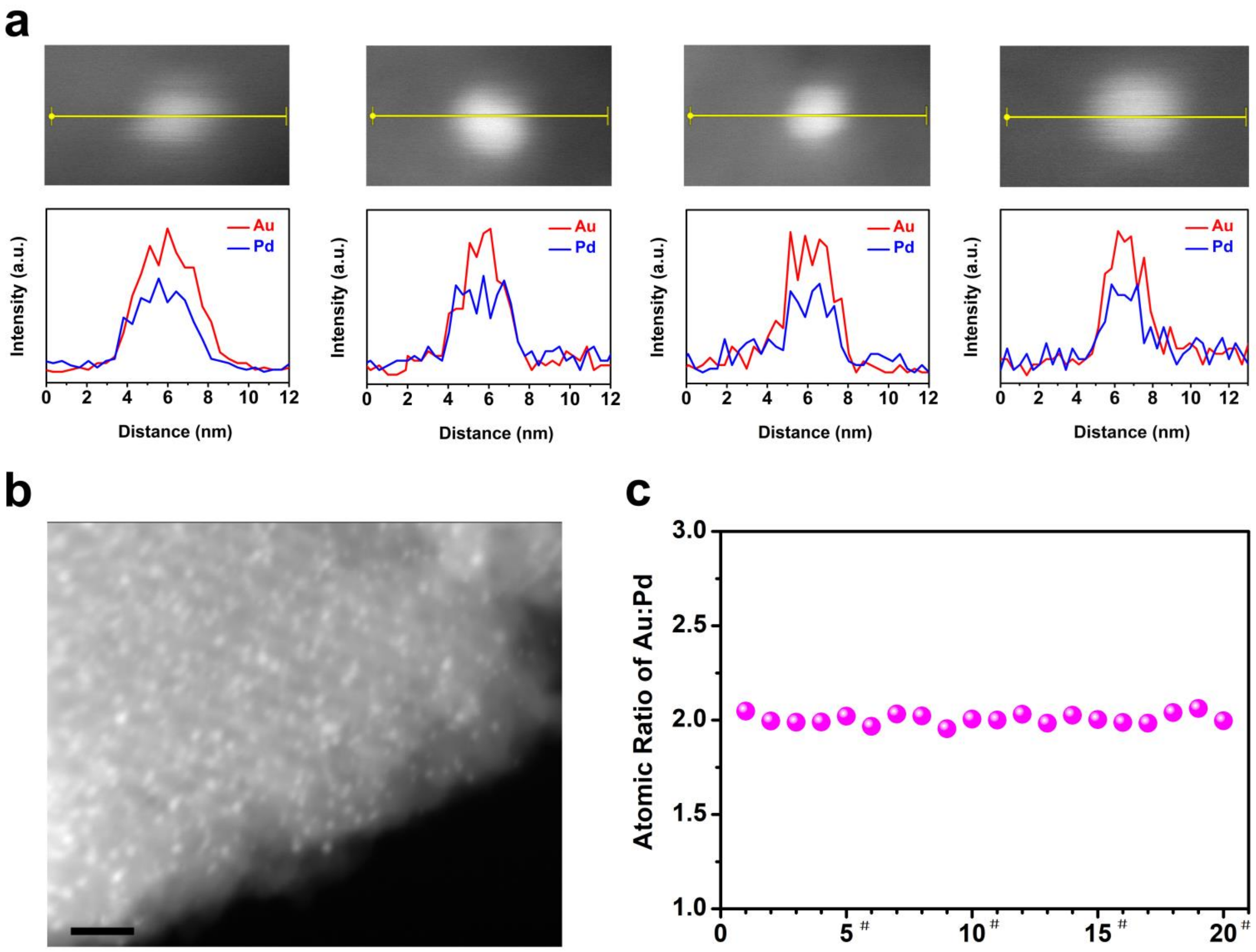

Supplementary Fig. 1. Composition distribution for $Au_{67}Pd_{33}$. **a**, Representative line profiles of the EDX patterns of four single particles collected using a focused electron beam in the sub-nanometre range in the scanning transmission electron microscopy (STEM) mode. **b**, STEM image. The scale bar is 20 nm. **c**, Statics of the individual composition of a single particle collected in different areas. Each dot represents a nanoparticle.

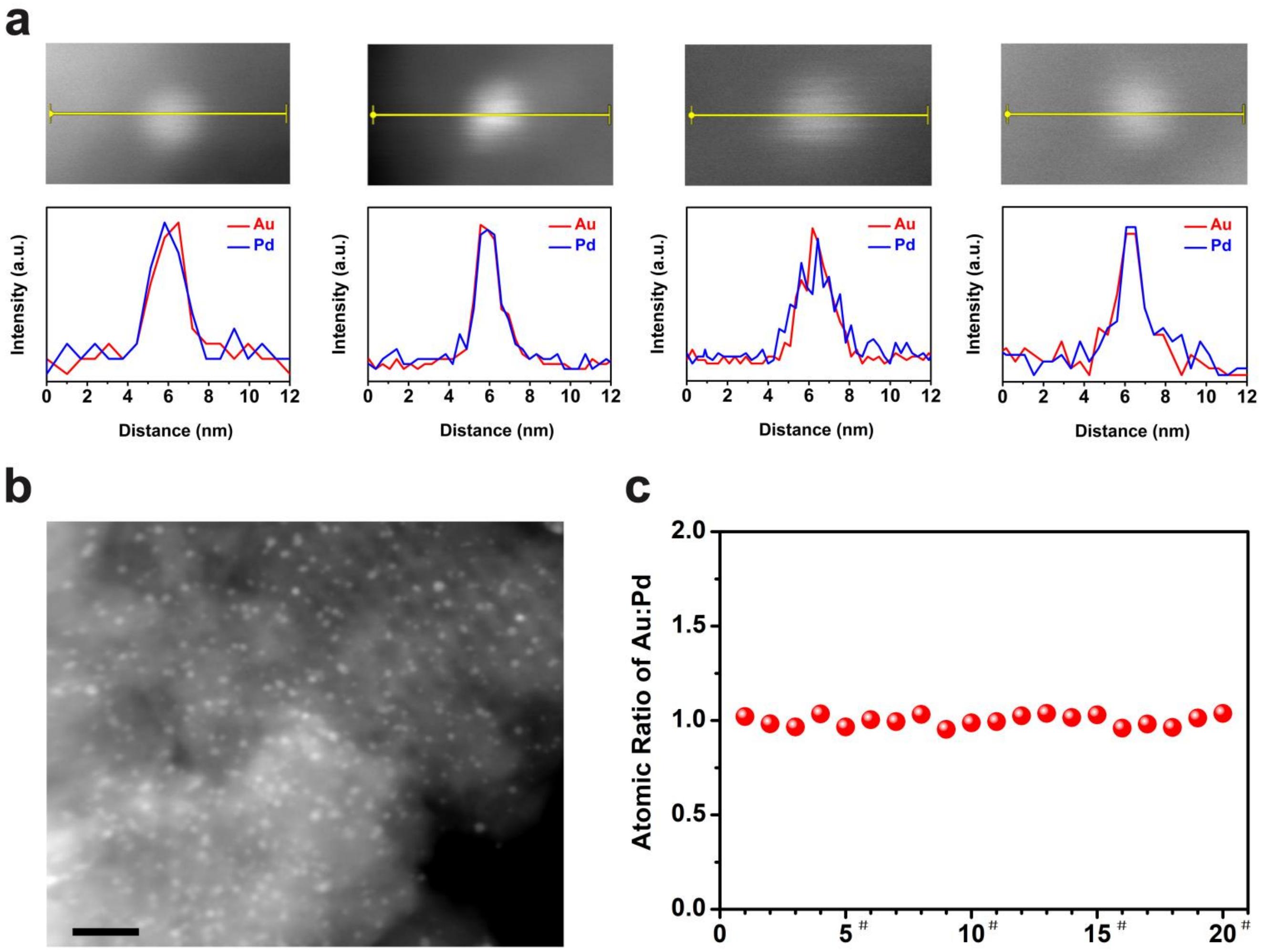

Supplementary Fig. 2. Composition distribution for $Au_{50}Pd_{50}$. **a**, Representative line profiles of the EDX patterns of four single particles collected using a focused electron beam in the sub-nanometre range in the STEM mode. **b**, STEM image. The scale bar is 20 nm. **c**, Statics of the individual composition of a single particle collected in different areas. Each dot represents a nanoparticle.

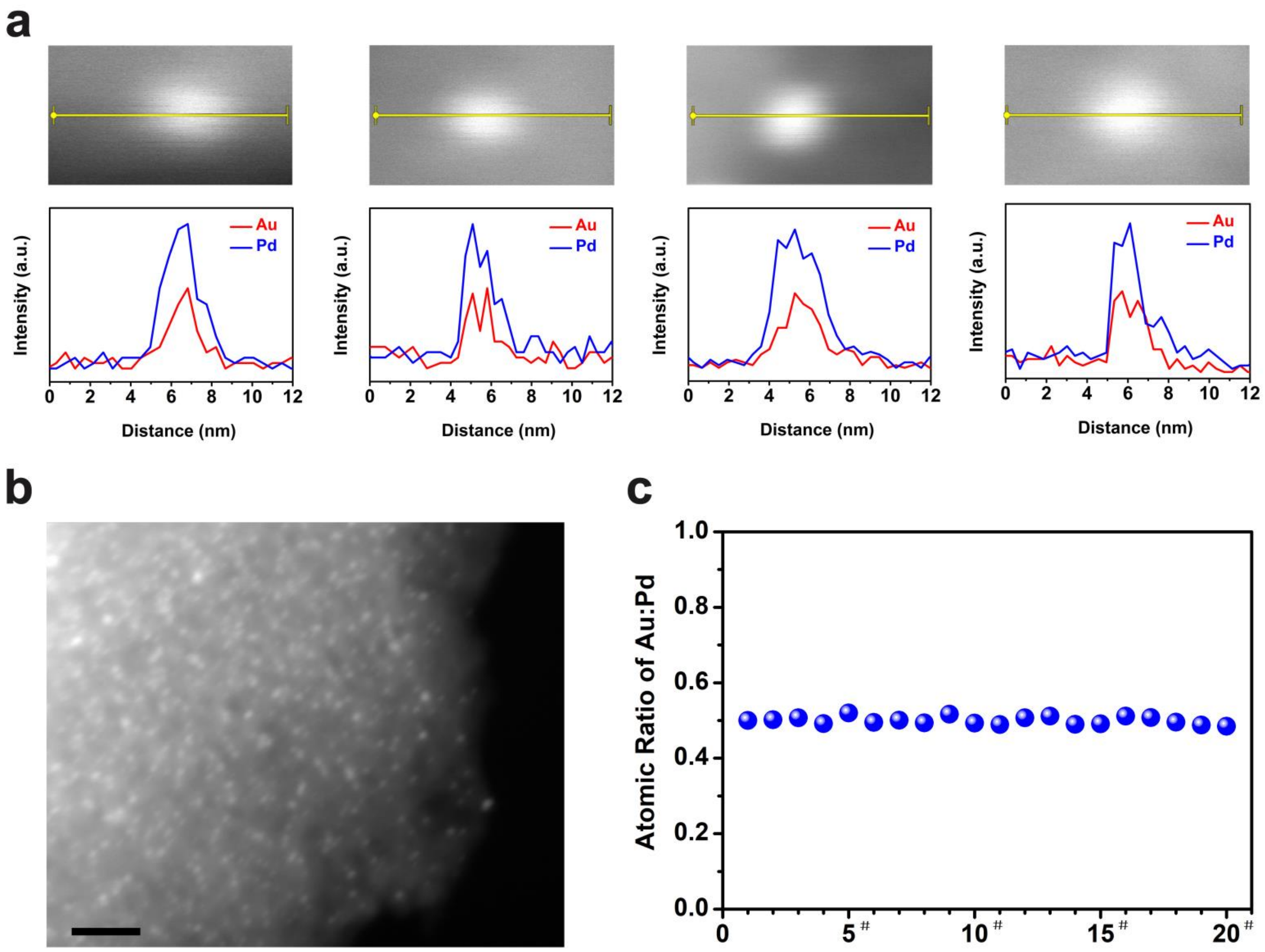

Supplementary Fig. 3. Composition distribution for $Au_{33}Pd_{67}$. **a**, Representative line profiles of the EDX patterns of four single particles collected using a focused electron beam in the sub-nanometre range in the STEM mode. **b**, STEM image. The scale bar is 20 nm. **c**, Statics of the individual composition of a single particle collected in different areas. Each dot represents a nanoparticle.

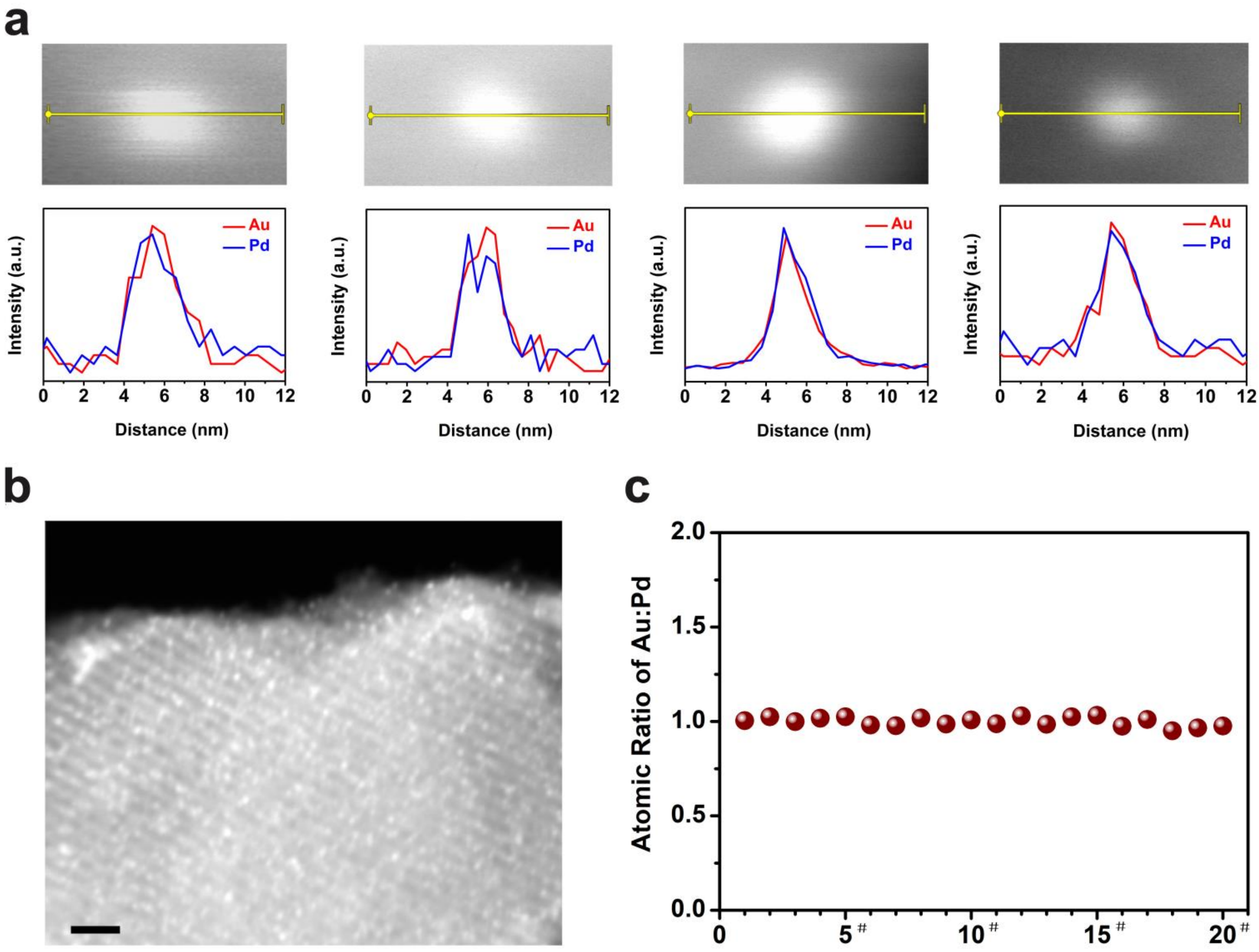

Supplementary Fig. 4. Composition distribution for the reused $Au_{50}Pd_{50}$-R6 catalyst. **a**, Representative line profiles of the EDX patterns of four single particles collected using a focused electron beam in the sub-nanometre range in the STEM mode. **b**, STEM image. The scale bar is 20 nm. **c**, Statics of the individual composition of a single particle collected in different areas. Each dot represents a nanoparticle.

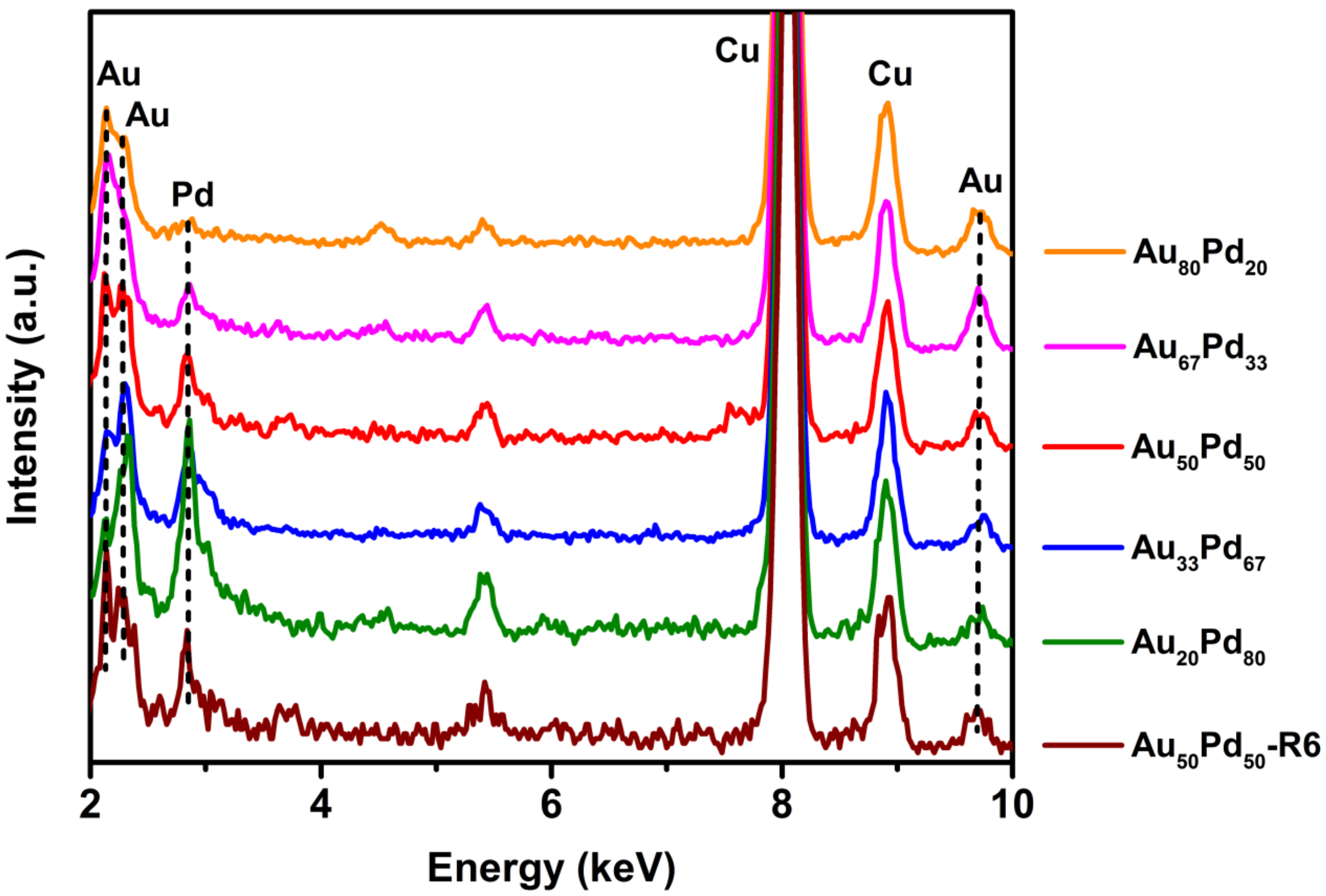

Supplementary Fig. 5. EDX spectra for bimetallic catalysts with different Pd contents. For comparison, the $Au_{50}Pd_{50}$-R6 catalyst after six catalytic runs is also provided.

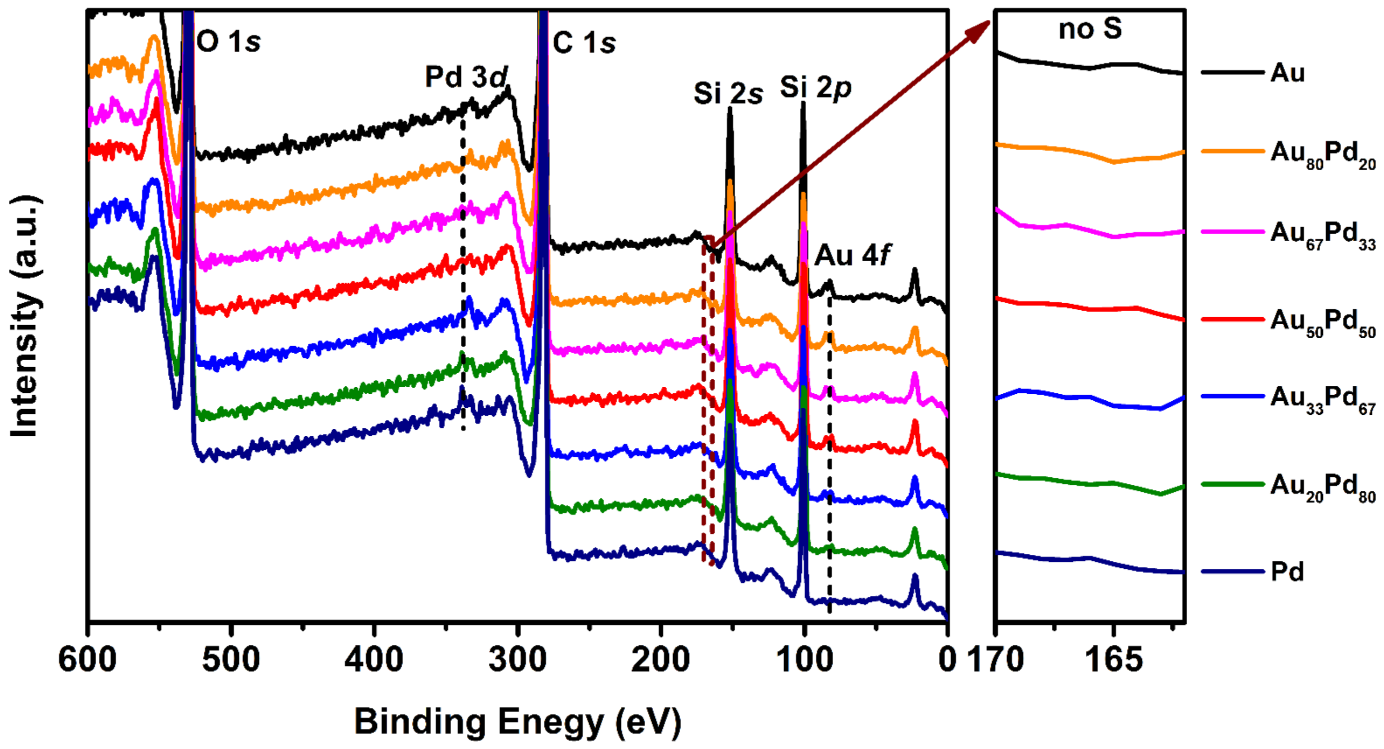

Supplementary Fig. 6. XPS spectra for monometallic and bimetallic catalysts.

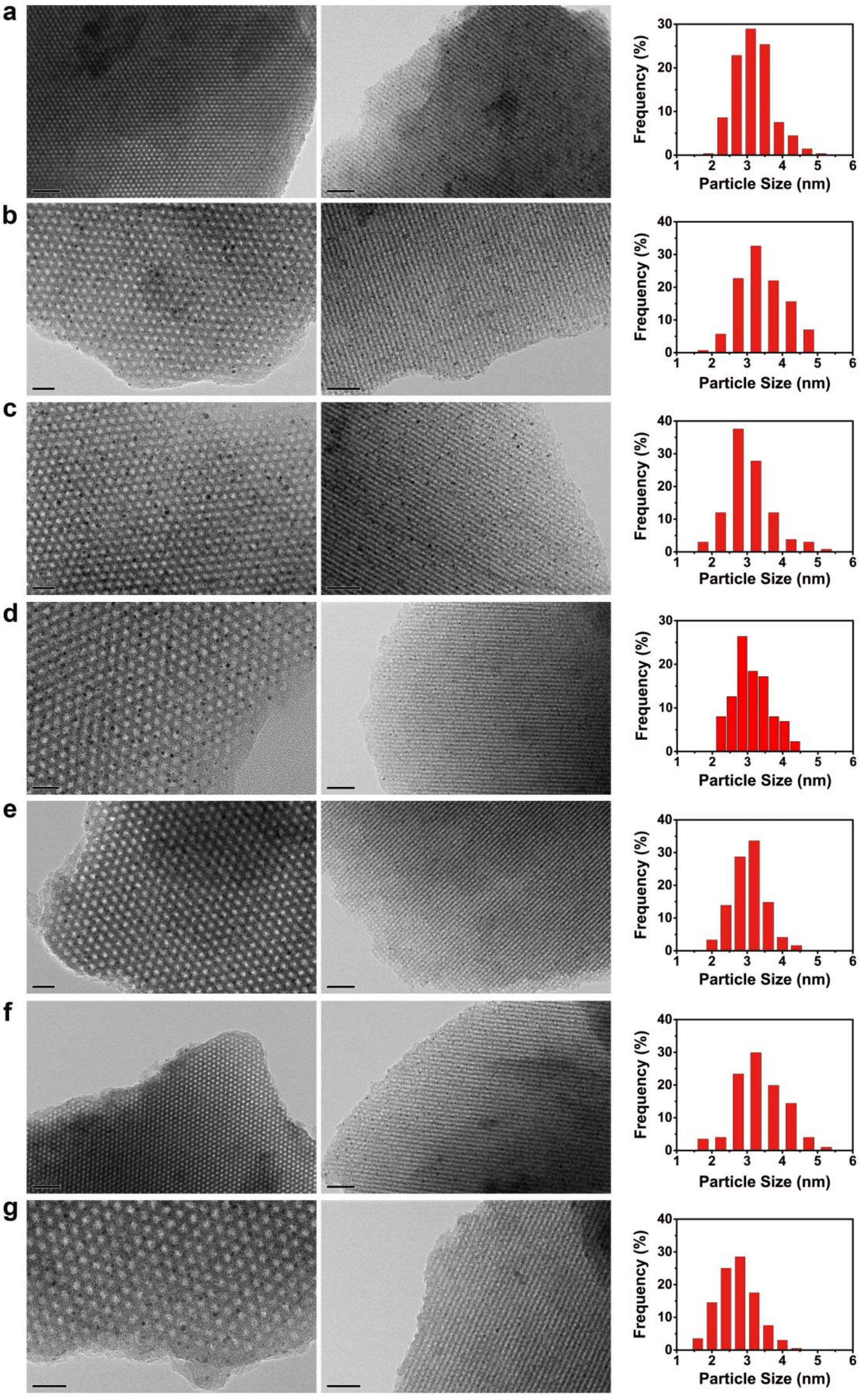

Supplementary Fig. 7. TEM images viewed along the [110] and [001] directions and particle size distribution counted by at least 200 nanoparticles for monometallic and bimetallic catalysts. **a**, Au. **b**, $Au_{80}Pd_{20}$. **c**, $Au_{67}Pd_{33}$. **d**, $Au_{50}Pd_{50}$. **e**, $Au_{33}Pd_{67}$. **f**, $Au_{20}Pd_{80}$. **g**, Pd. The scale bar is 50 nm, except for Left b,c,d,e and g, in which the scale bar is 20 nm.

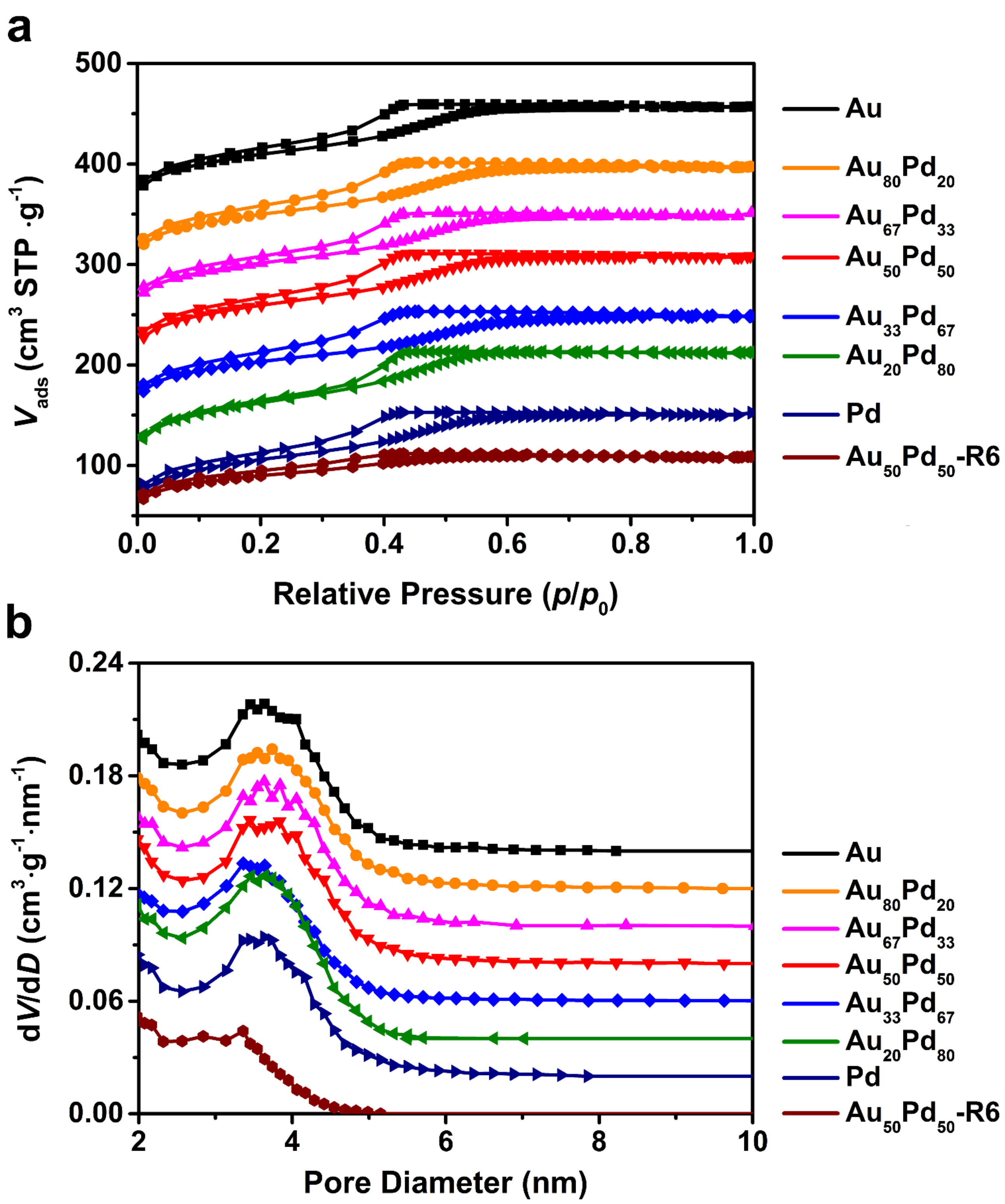

Supplementary Fig. 8. Textural properties. **a**, N_2 sorption isotherms and **b**, Pore size distribution curves for fresh Au, Pd and AuPd nanoalloys with different compositions. The used $Au_{50}Pd_{50}$-R6 catalyst is also measured and calculated. The sorption isotherms are vertically offset by 50, 100, 150, 200, 250, and 300 cm^3 g^{-1} from Pd to Au. The pore size distribution curves are vertically offset by 0.02, 0.04, 0.06, 0.08, 0.10, and 0.12 cm^3 g^{-1} from Pd to Au.

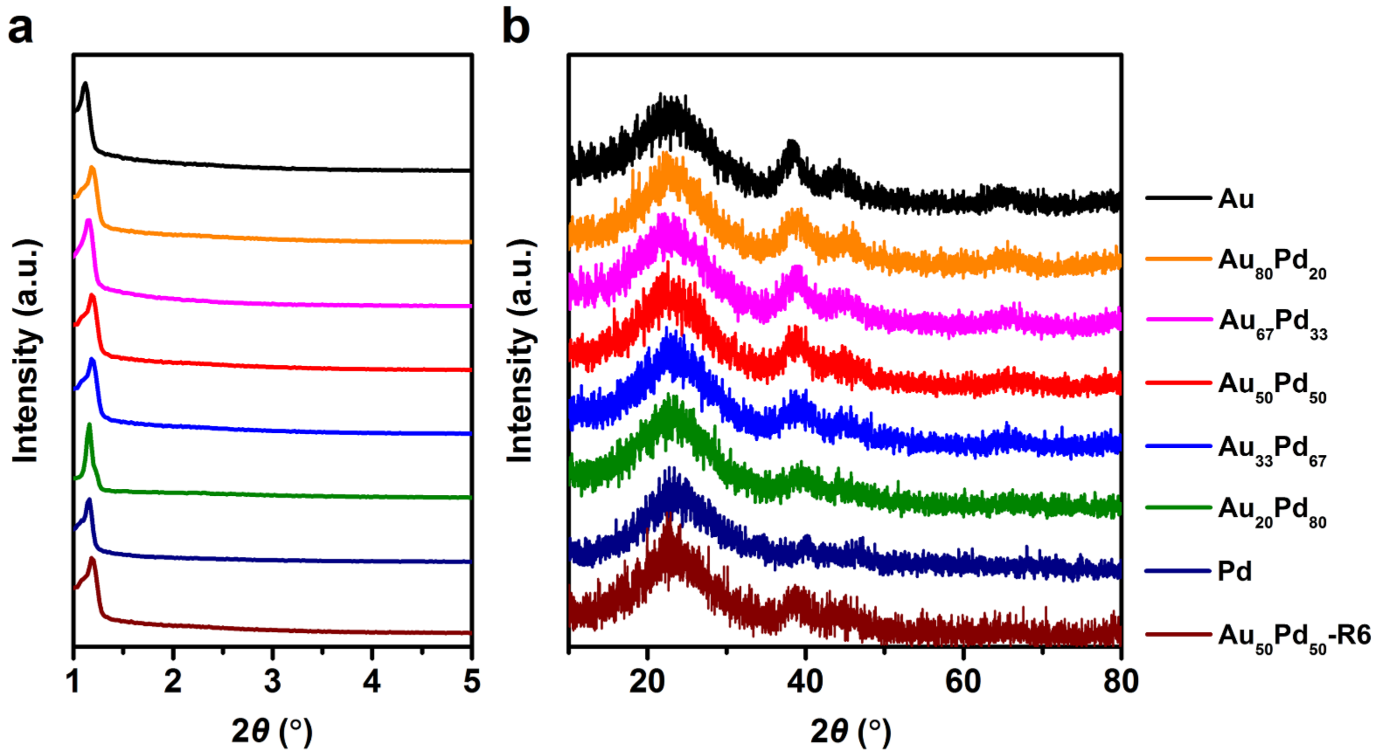

Supplementary Fig. 9. XRD patterns. **a**, Small-angle and **b**, Wide-angle XRD patterns for monometallic and bimetallic catalysts. For comparison, the $Au_{50}Pd_{50}$-R6 catalyst after six catalytic runs is also provided.

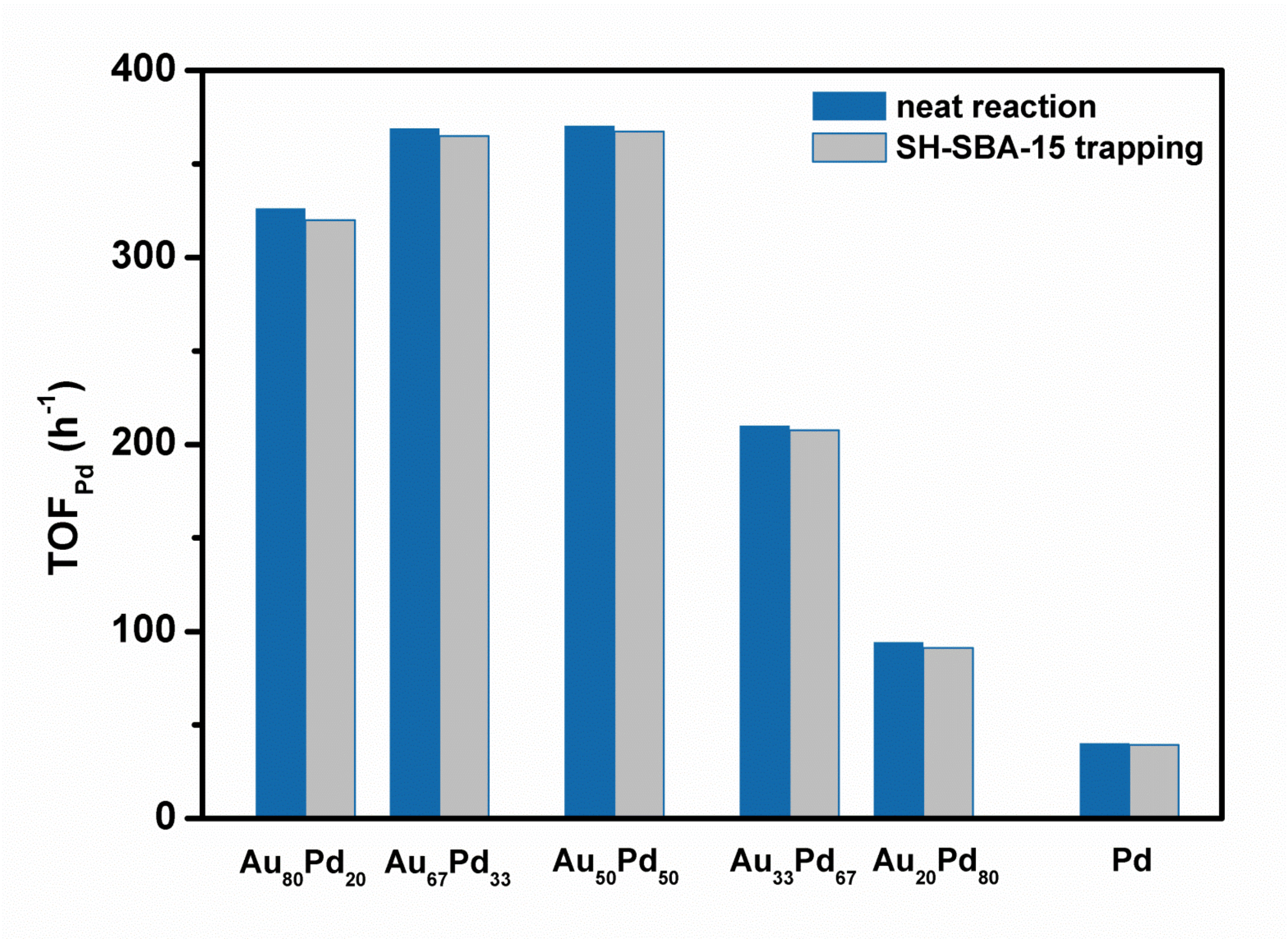

Supplementary Fig. 10. Trapping test. Comparison of the conversion of benzyl alcohol at 90 ℃ in the absence (solid) and presence of SH-SBA-15 (shadow) over monometallic Pd and AuPd alloy nanocatalysts with different compositions. The reaction conditions were: 32 mg of catalyst; 5.0 mmol of substrate; 10 mL of water; 90 ℃; and in the presence of oxygen by an O_2 balloon under atmospheric pressure.

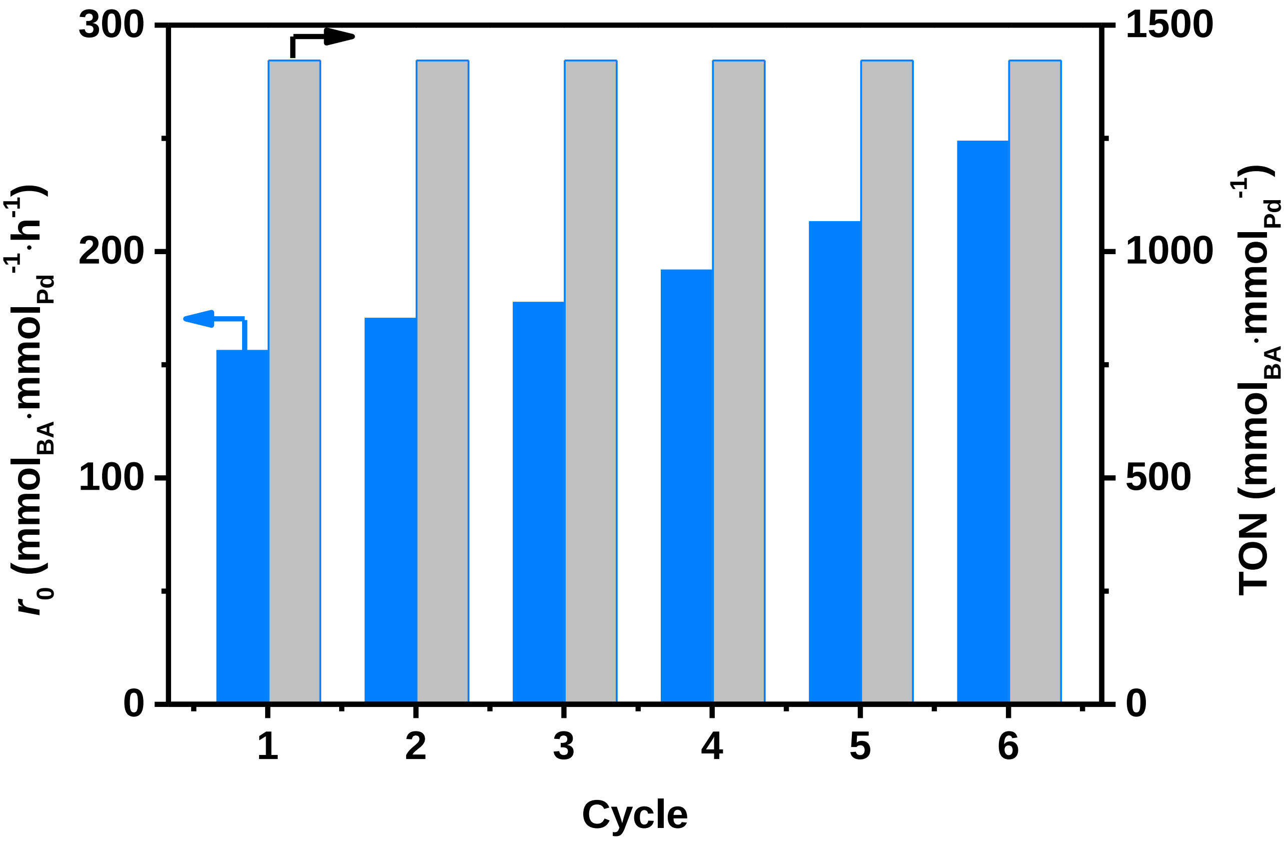

Supplementary Fig. 11. Reusability. The comparison of the initial reaction rate (r_0) and the turn over number (TON) for the $Au_{50}Pd_{50}$ catalyst in the successive cycles for the selective oxidation of benzyl alcohol in the presence of oxygen. The reaction conditions were: 32 mg of catalyst; 5.0 mmol of substrate; 10 mL of water; 90 ℃; and in the presence of oxygen by an O_2 balloon under atmospheric pressure.

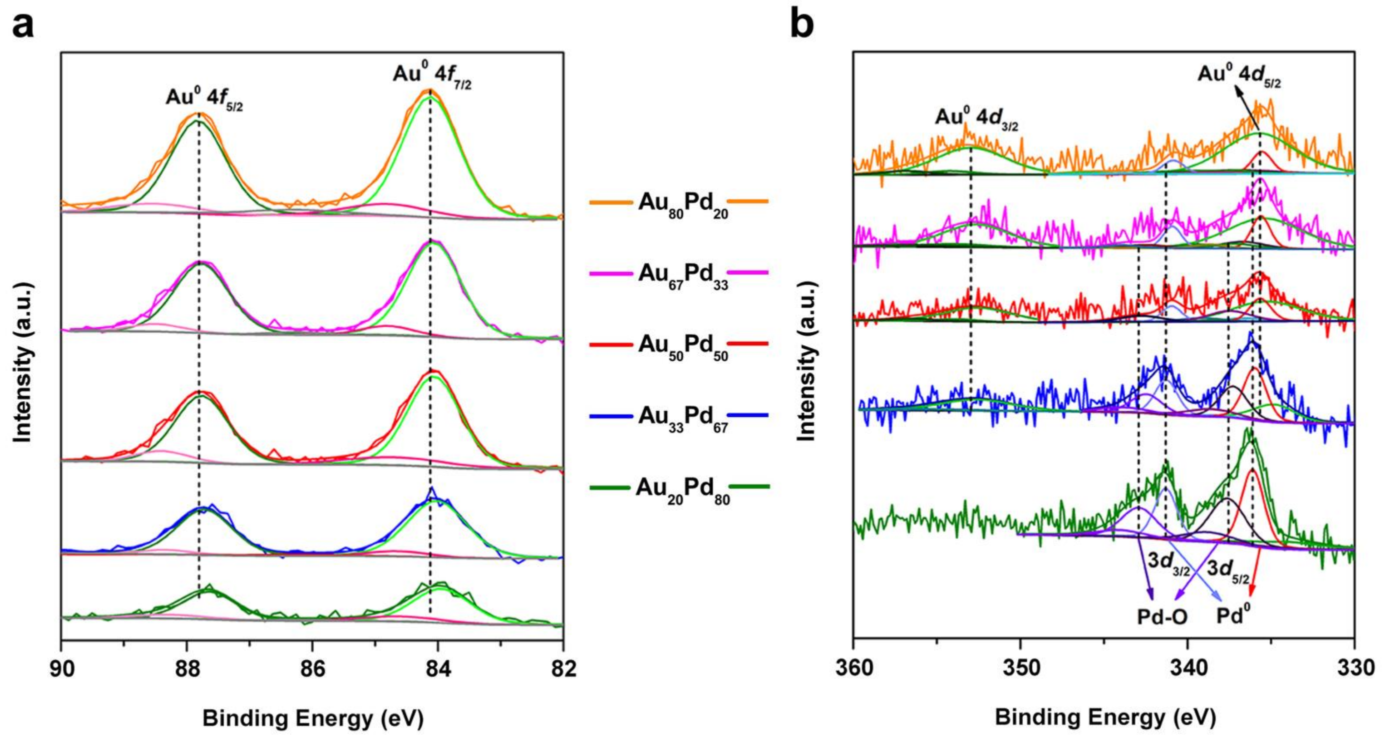

Supplementary Fig. 12. XPS spectra for the AuPd nanocatalysts after reaction. **a**, XPS spectra of the 4*f* level of Au. **b**, XPS spectra of the 3*d* level of Pd. The Au and Pd binding energies were fitted by peak fitting techniques. Metallic Au, metallic Pd and Pd-O, which were the dominant contributors to each spectrum, were discussed. A distinct overlap could be observed between the Pd 3$d_{5/2}$ and the Au 4$d_{5/2}$ components for the bimetallic catalysts. The Au 4$d_{5/2}$ intensity was calculated from the well-resolved Au 4$f_{7/2}$ intensity, and this value was subtracted from the above overlapped peak to determine the Pd 3$d_{5/2}$ intensity. The resulting value was used to calculate the Au:Pd ratio.

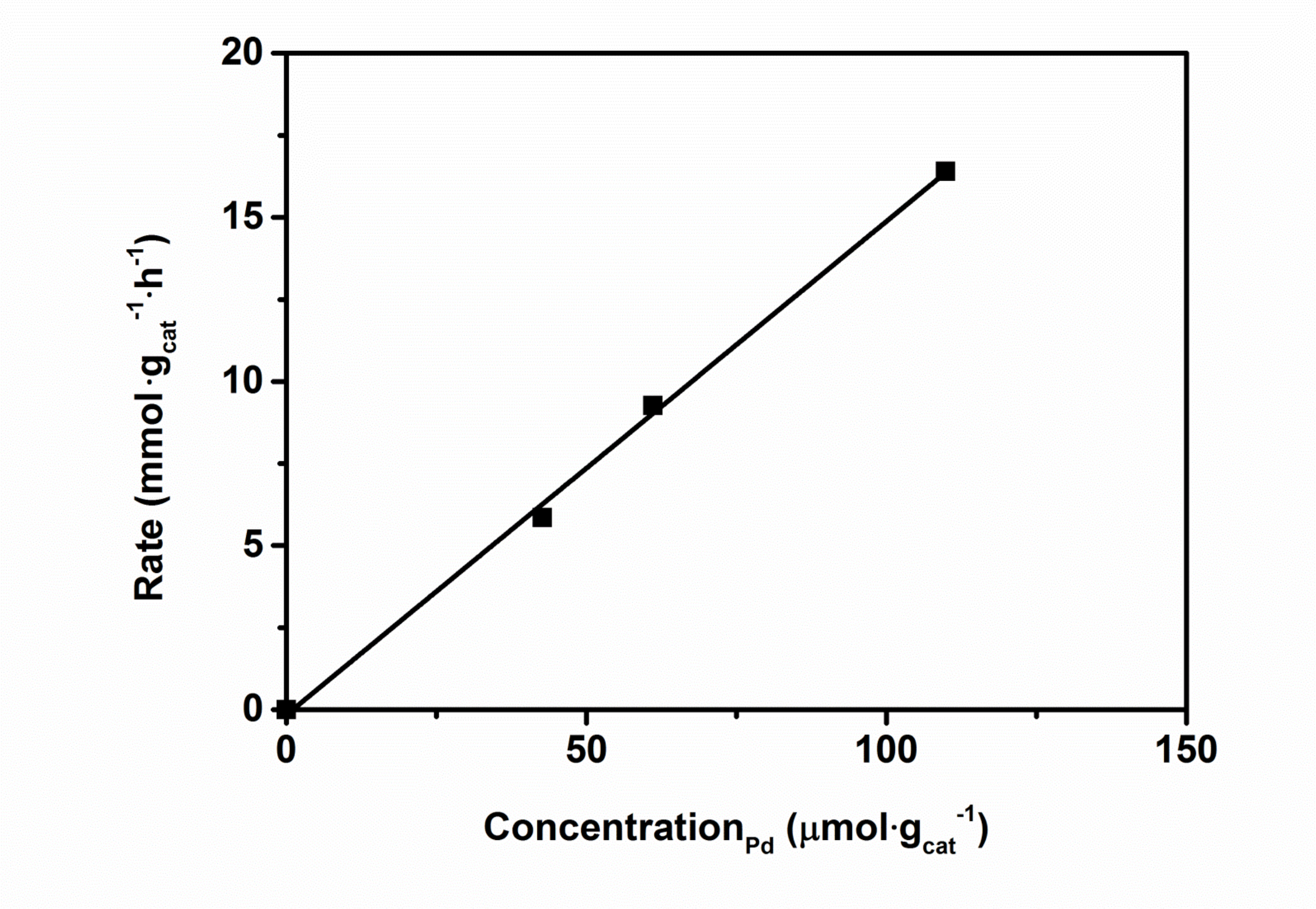

Supplementary Fig. 13. Effect of the Pd loading (the Madon-Boudart test)[1,2] on the reaction rate of benzyl alcohol oxidation using the $Au_{50}Pd_{50}$ catalyst (Au:Pd molar ratio of 1). The reaction conditions were: 5.0 mmol benzyl alcohol; 10 mL H_2O; 32 mg of catalyst; 90 ℃; 800 rpm; and in the presence of oxygen by an O_2 balloon under atmospheric pressure.

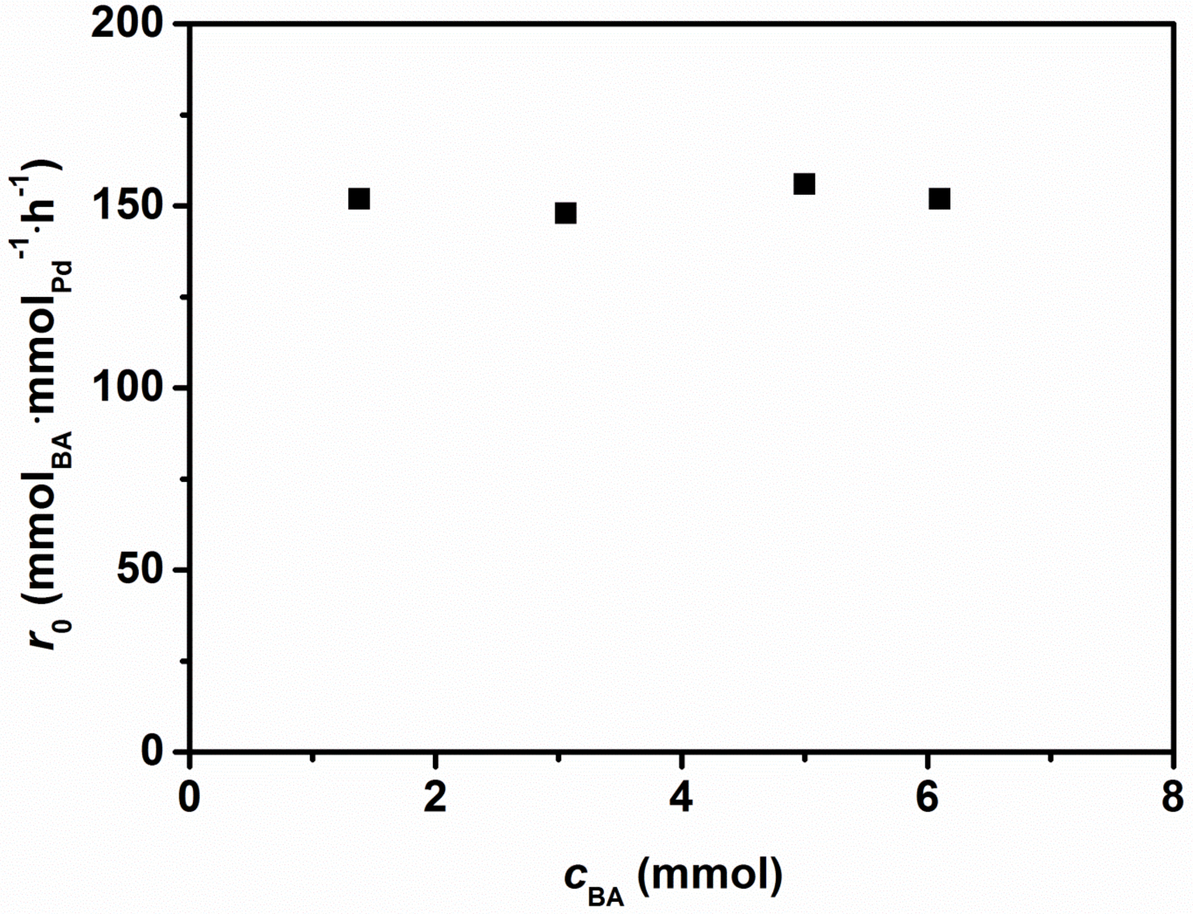

Supplementary Fig. 14. Effect of benzyl alcohol concentration on the overall rate of oxidation over the $Au_{50}Pd_{50}$ catalyst.

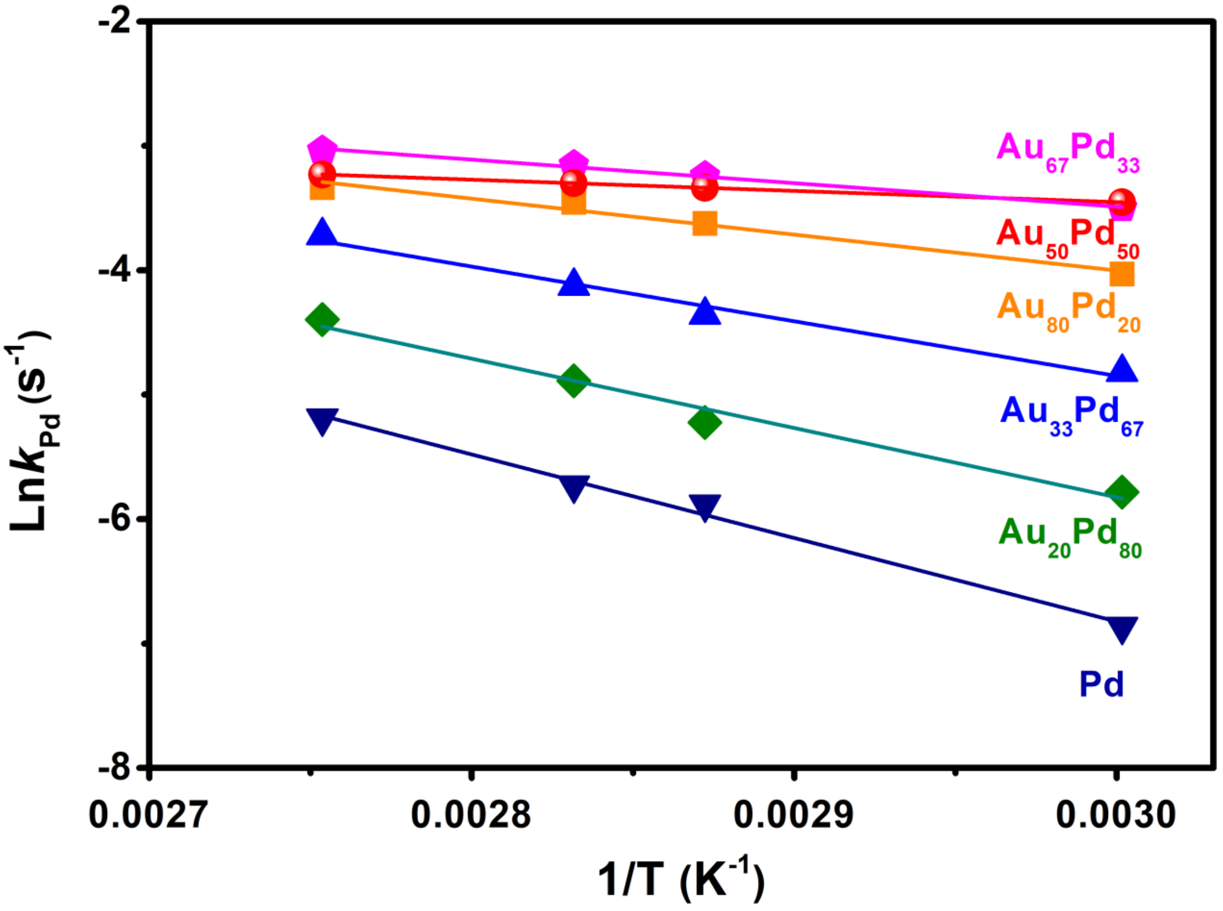

Supplementary Fig. 15. Arrhenius plots for the benzyl alcohol oxidation over monometallic Pd and AuPd alloy nanocatalysts with different compositions. Activation energies E_a, were calculated according to the Arrhenius equation:

$$\ln k = \ln A - \frac{E_a}{RT} \tag{1}$$

where A is the pre-exponential factor, R is the universal gas constant and T is the reaction temperature.

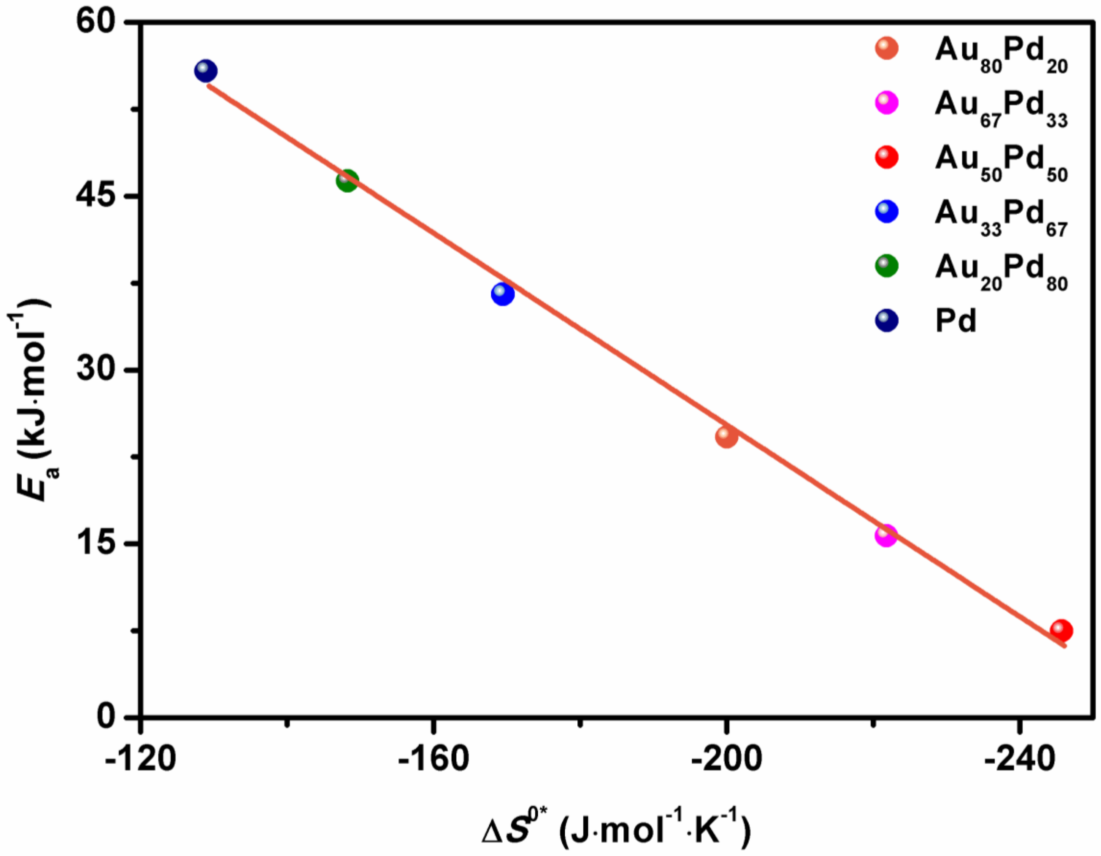

Supplementary Fig. 16. Plot of activation energy (E_a) against entropy change (ΔS^{0*}) for benzyl alcohol oxidation over monometallic Pd and AuPd alloy nanocatalysts with different compositions.

The turn over frequency (TOF) for each catalyst was calculated on the basis of the estimated number of exposed palladium atoms. This value was calculated at less than 25% conversion,

$$\mathrm{TOF_{Pd}} = \frac{n_{\mathrm{BA}}X}{n_{\mathrm{Pd}}t\tau} \tag{2}$$

where X is the conversion and t is the reaction time. Although microstructural deviations occurred in the AuPd nanoalloys, we assumed that the value for the exposed surface atom dispersion (τ) calculated from equation (3) - (6), which was based on the similarities of the truncated octahedron shape of the nanoparticles (as evidenced in the aberration corrected STEM image) and the homogeneous distribution in the alloy, was accurate[3-6].

$$d_{\mathrm{particle}} = 1.105 \times N_{\mathrm{T}}^{1/3} \times d_{\mathrm{AuPd}} \tag{3}$$

$$N_{\mathrm{T}} = 16m^3\text{-}33m^2\text{+}24m\text{-}6 \tag{4}$$

$$N_{\mathrm{S}} = 30m^2\text{-}60m\text{+}32 \tag{5}$$

$$\tau = N_{\mathrm{S}}/N_{\mathrm{T}} \tag{6}$$

Wherein, d_{particle} is the diameter of particle, N_{T} is the total atom number of each particle, N_{S} is the surface atom number, and m is the number of atoms lying on an equivalent edge (corner atoms included). Note that the diameter of Au (d_{Au})

and Pd (d_{Pd}) atom is 0.2884 and 0.2751 nm, respectively, an average atom diameter of 0.2818 nm for d_{AuPd} is adopted. The enthalpy of activation is determined as following:

The TOF value can be expressed in the Eyring form[7,8]:

$$\mathrm{TOF_{Pd}} = \frac{k_B T}{h} \exp\left(\frac{\Delta S^{0*}}{R}\right) \exp\left(-\frac{\Delta H^{0*}}{RT}\right) \quad (7)$$

where k_B, h, ΔS^{0*}, and ΔH^{0*} are the Boltzmann constant, Planck constant, entropy of activation, and enthalpy of activation, respectively.

The activation energy E_a could be related to ΔH^{0*} by the Temkin equation:

$$E_a = \Delta H^{0*} + \sum n_i \Delta H_i \quad (8)$$

where ΔH_i and n_i are the adsorption enthalpies and the reaction order of reactant i, respectively. Taking into account the approximate zero-order reaction kinetics for benzyl alcohol, E_a is simplified to be close to ΔH^{0*}.

The entropy change in the activation step of the chemical reaction is closely related to the thermodynamics of the rate constant, which results in the following equation:

$$\Delta S^{0*} = R \ln\left(\frac{Ah}{k_B T \tau}\right) \quad (9)$$

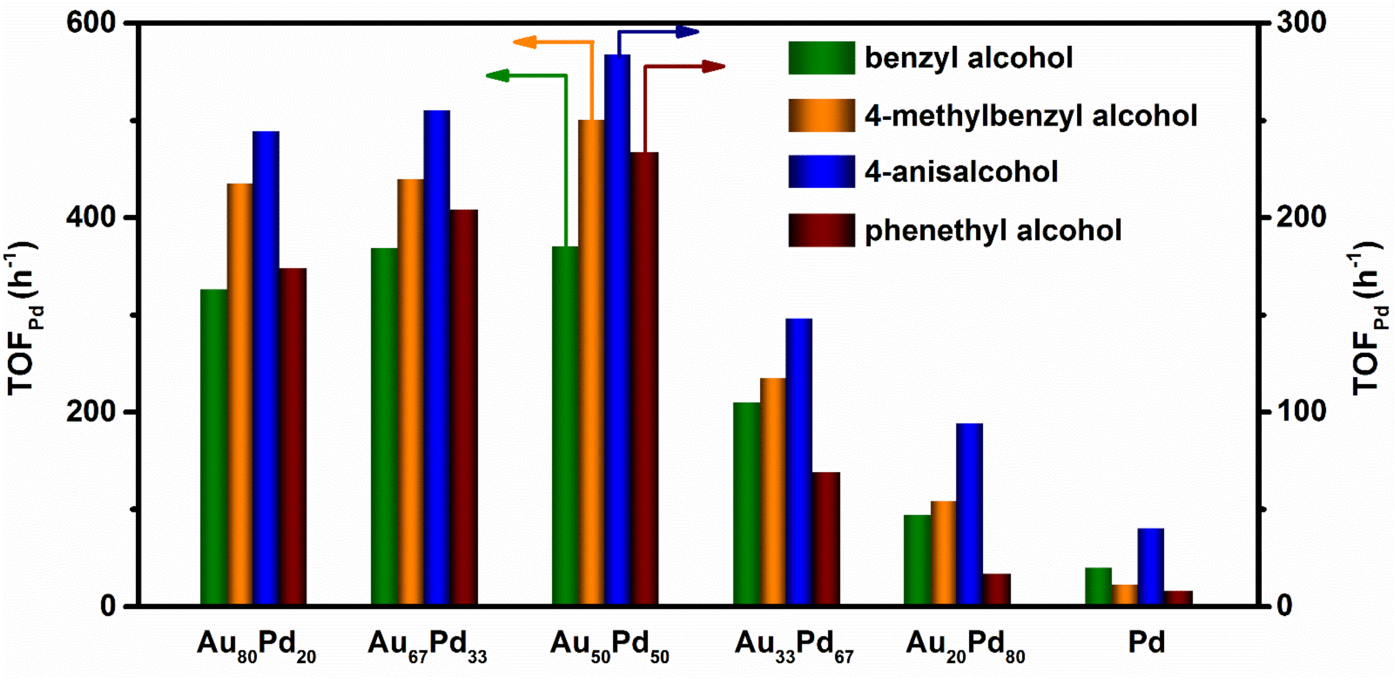

Supplementary Fig. 17. Comparison of the TOF_{Pd} values of the nanocatalysts along with monometallic Pd and AuPd alloy nanocatalysts with different compositions in the oxidations of benzyl alcohol, 4-methylbenzyl alcohol, 4-anisalcohol and phenethyl alcohol. The reaction conditions were: 32 mg of catalyst; 5.0 mmol of substrate; 10 mL of water; 90 ℃; and in the presence of oxygen by an O_2 balloon under atmospheric pressure.

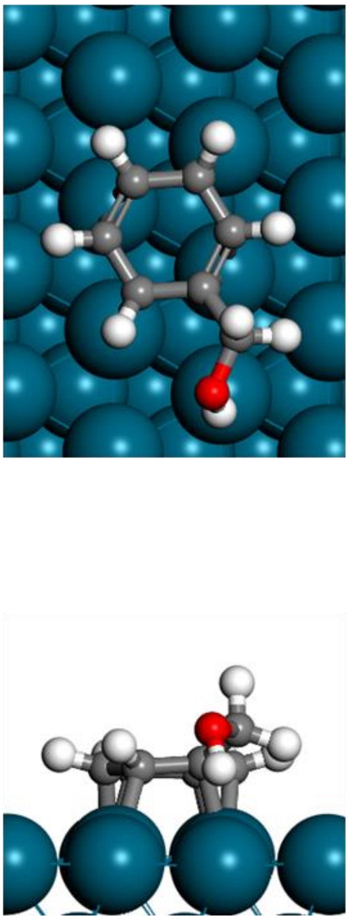

Supplementary Fig. 18. The most favourite adsorption configuration of benzyl alcohol on Pd(111) from the top and side views: paralleled adsorption mode via aromatic ring.

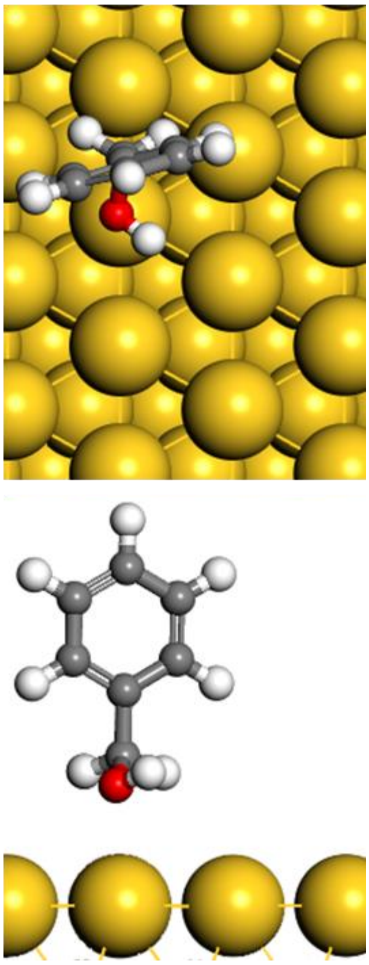

Supplementary Fig. 19. The most favourite adsorption configuration of benzyl alcohol on Au(111) from the top and side views: physical vertical adsorption mode via hydroxyl group.

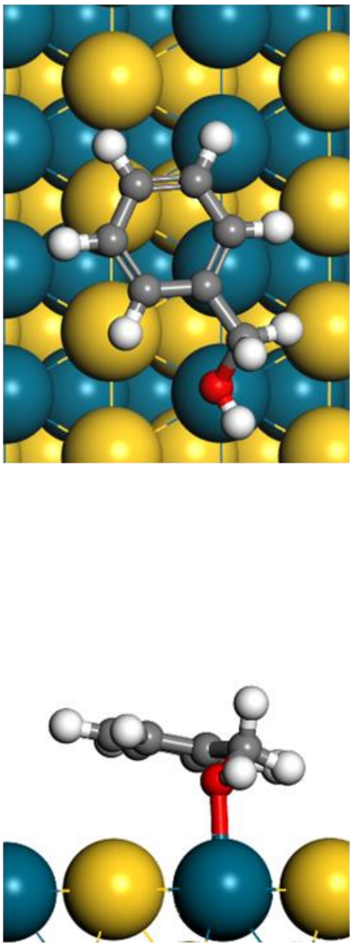

Supplementary Fig. 20. The most favourite adsorption configuration of benzyl alcohol on $Au_{50}Pd_{50}(111)$ from the top and side views: tilted adsorption mode via hydroxyl group.

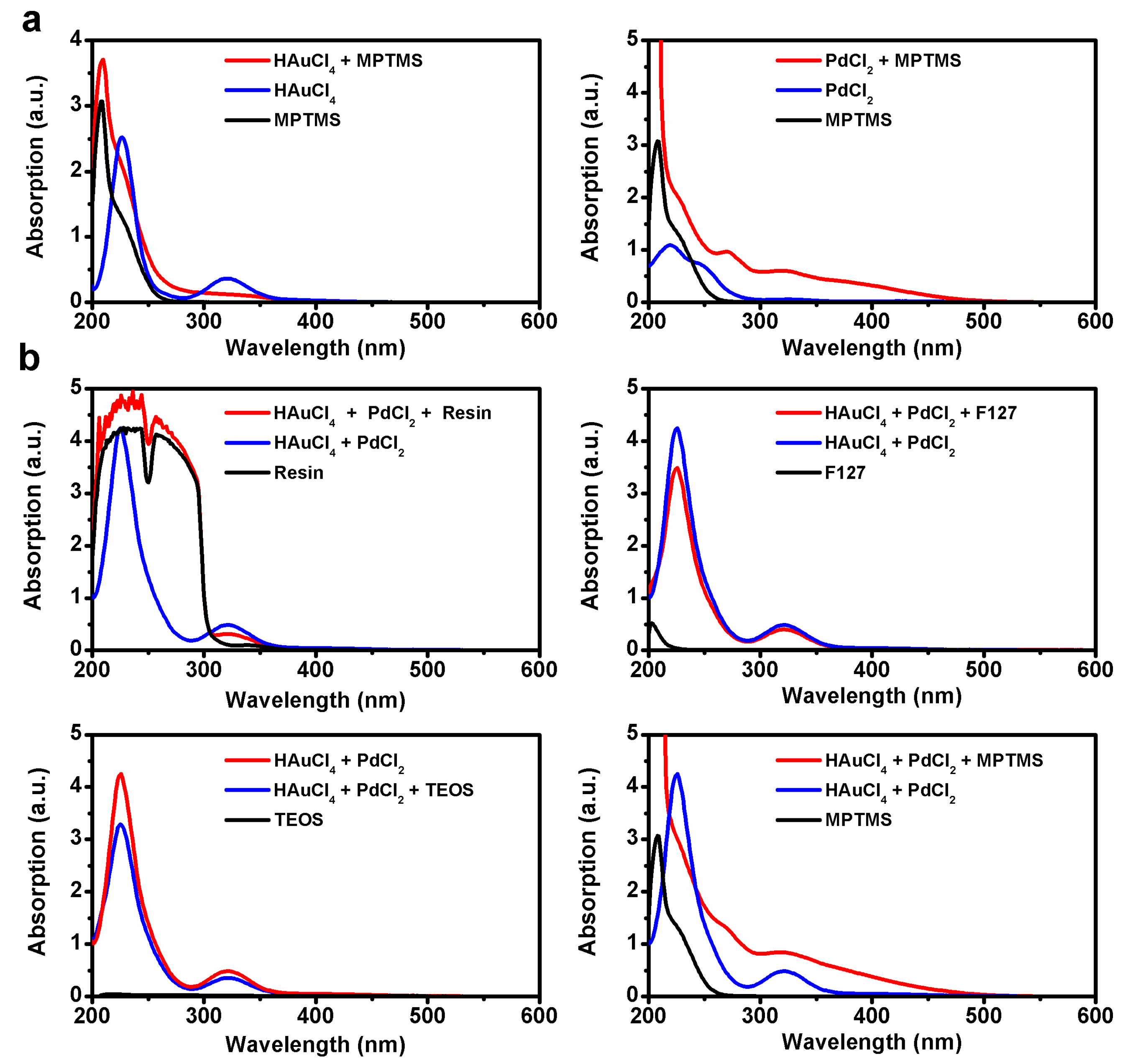

Supplementary Fig. 21. UV-Vis diffuse reflectance spectra. **a**, Individual ethanolic $HAuCl_4$ or $PdCl_2$ solution and MPTMS, and the mixture of the two precursors. **b**, Individual phenolic resin, F127, TEOS, or MPTMS, the mixture of ethanolic $HAuCl_4$ and $PdCl_2$ solution, and the mixture of them. Individual interaction between ethanolic $HAuCl_4$ solution with MPTMS showed a remarkable reduction on the absorbance intensity of Au-Cl coordination originated by the $\pi \rightarrow \sigma^*$ orbital transition, suggesting the ligand exchange by Au-S[9]. The presence of MPTMS in the ethanolic $PdCl_2$ solution led to several bands around 270, 320 and 404 nm. The last one was possibly dominated by the d-π^* transition accompanied by a π-π^* transition in [S-Pd(II)-S][10]. These results suggested the ligand exchange by Au-S and Pd-S. Each reactant was separately added into the mixture of $HAuCl_4$ and $PdCl_2$ solution. Significant change only occurred in the UV-Vis spectrum with addition of MPTMS, further suggesting the complexation of -SH with metal ions.

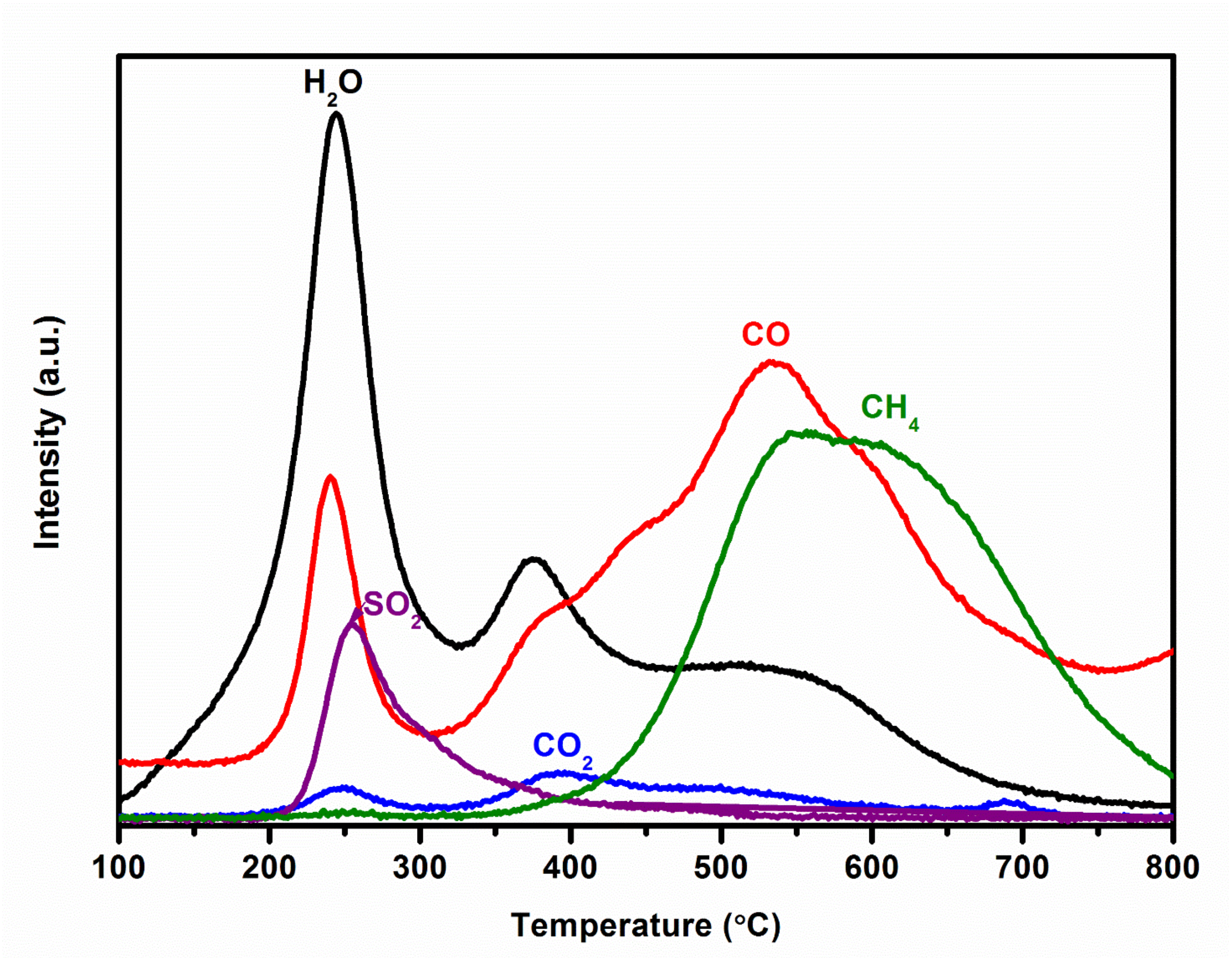

Supplementary Fig. 22. TPD-MS spectra for as-prepared $Au_{50}Pd_{50}$. In the TPD measurements, the sample (ca. 50 mg) was purged at 50 ℃ for 1 h in flowing dry He (40 mL min^{-1}, >99.999%). Then, the sample was heated to 800 ℃ at a rate of 10 ℃ min^{-1} in flowing dry He (40 mL min^{-1}, >99.999%) and maintained at 800 ℃ for 20 minutes. The signals of mass-to-charge (m/z) ratios of 16, 18, 28, 44, and 64 were recorded.

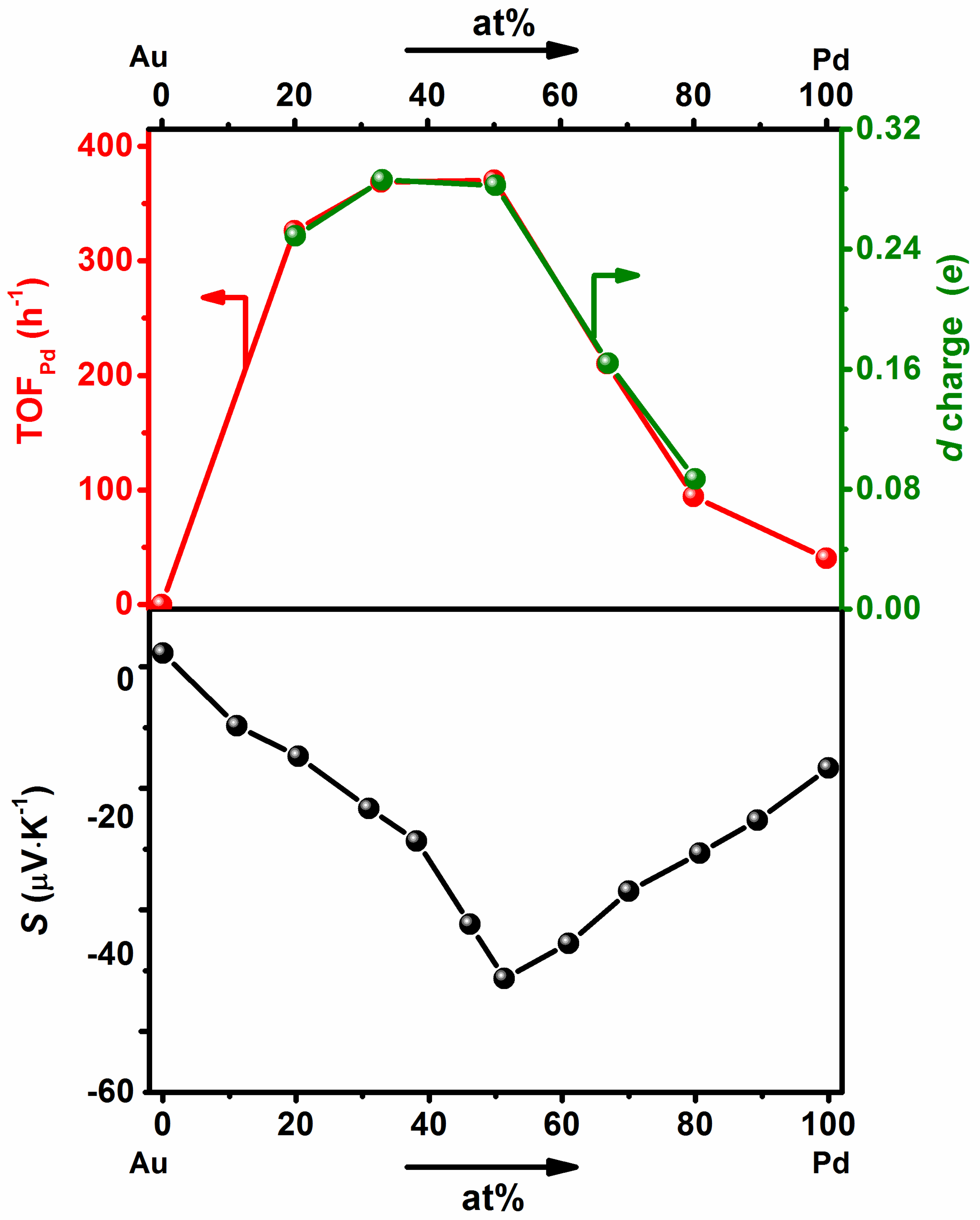

Supplementary Fig. 23. Relationship between the thermoelectric power (*S*), *d* charge at Pd site and TOF_{Pd} of the AuPd alloys as a function of Pd concentration. The values for thermoelectric power for the AuPd alloys as a function of Pd concentration were obtained from the Ref. 11.

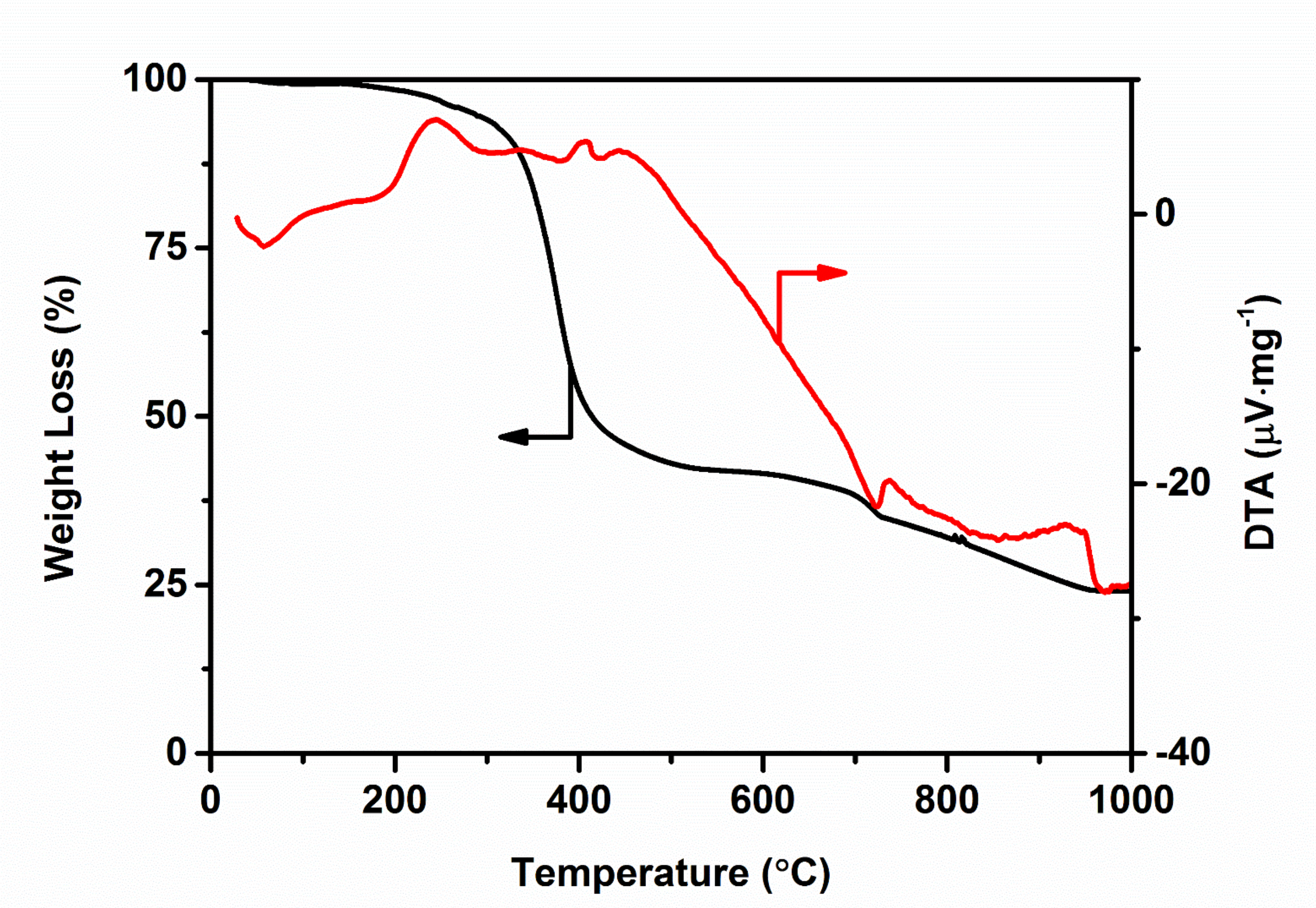

Supplementary Fig. 24. TG/DTA curves carried out in nitrogen atmosphere for as-made $Au_{50}Pd_{50}$.

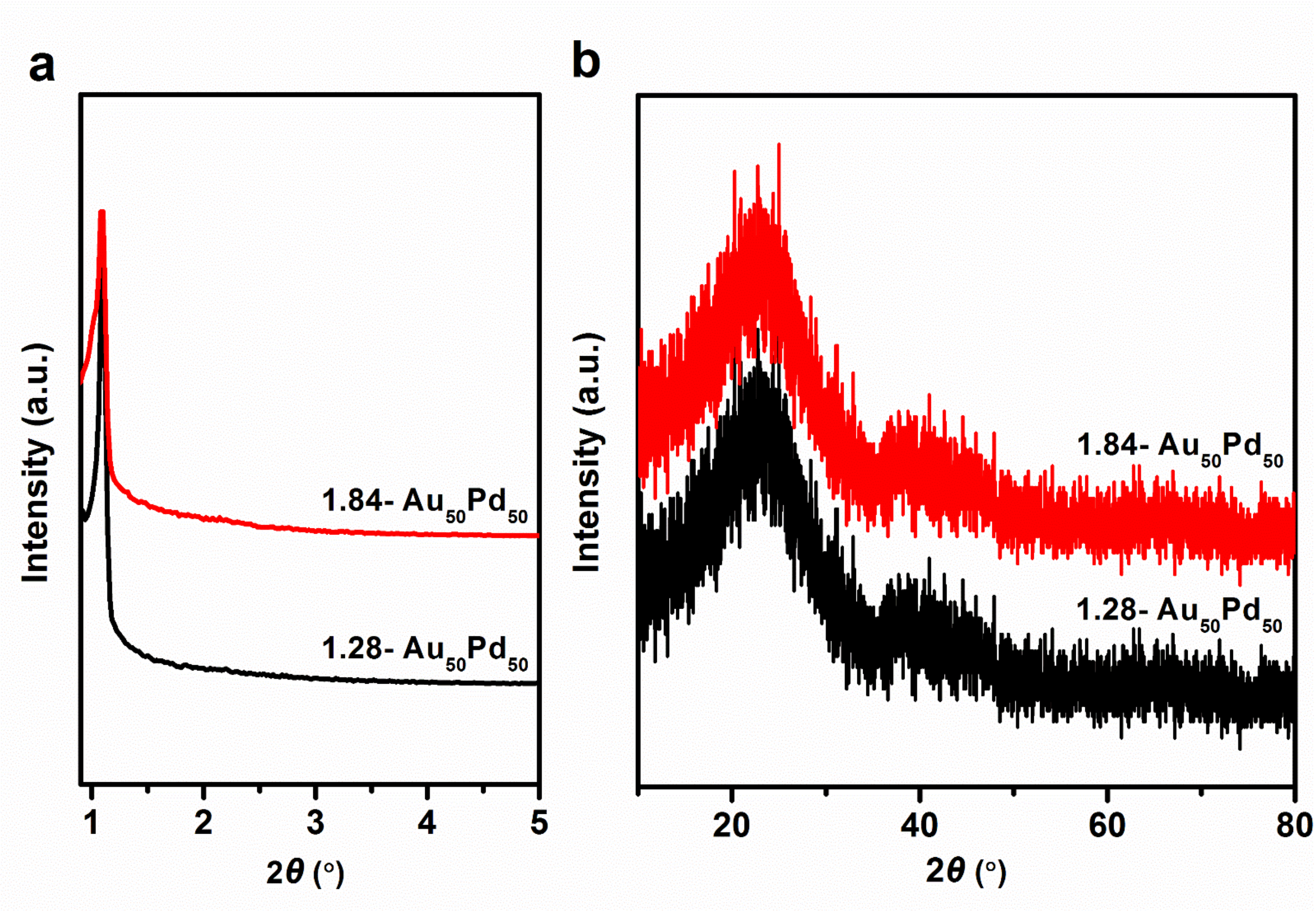

Supplementary Fig. 25. Structural characterization of $Au_{50}Pd_{50}$ catalysts with total metal contents of 1.28 and 1.84 wt%. **a**, Small-angle XRD patterns. **b**, Wide-angle XRD patterns.

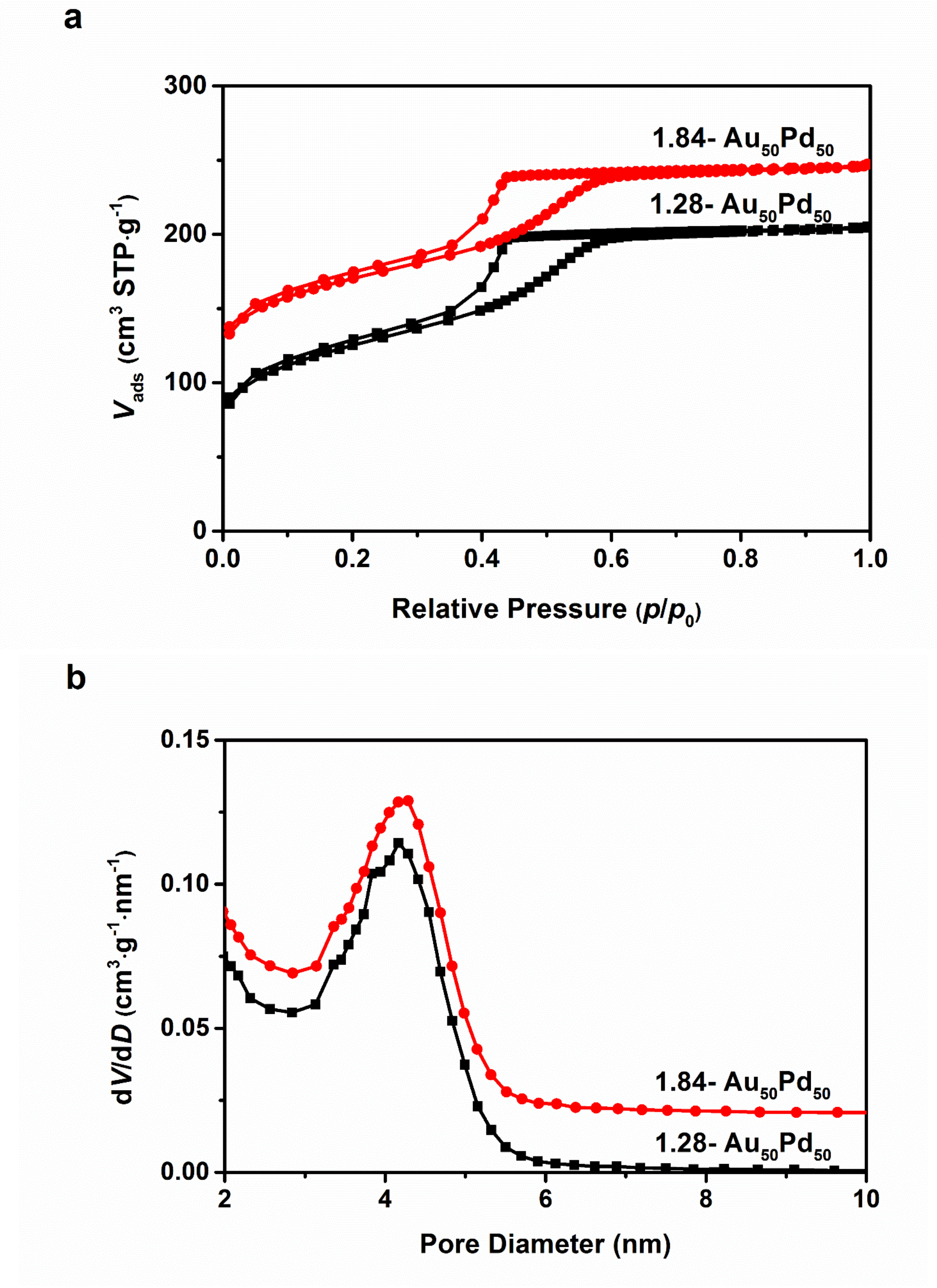

Supplementary Fig. 26. Physisorption character of $Au_{50}Pd_{50}$ catalysts with total metal contents of 1.28 and 1.84 wt%. **a**, N_2 sorption isotherms. **b**, Pore-size distribution curves.

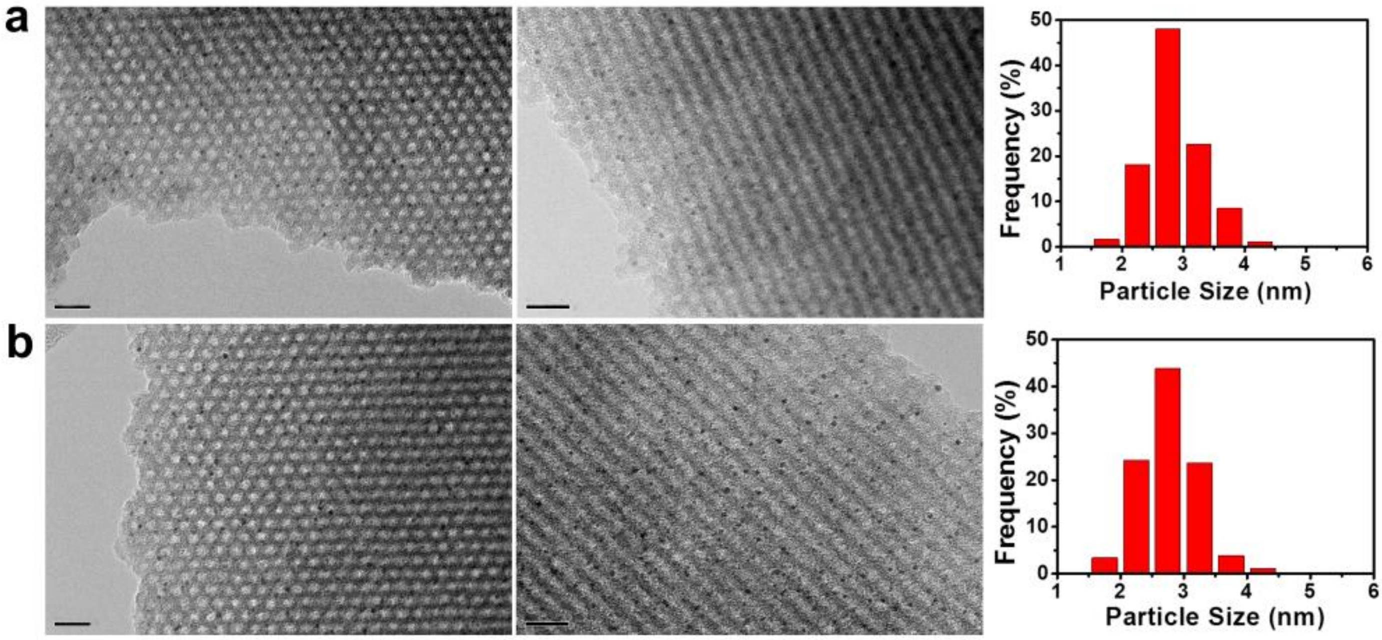

Supplementary Fig. 27. TEM images viewed along the [110] and [001] directions and particle size distribution counted by at least 200 nanoparticles for $Au_{50}Pd_{50}$ catalysts with total metal contents of **a**, 1.28 wt% and **b**, 1.84 wt%. The scale bar is 20 nm.

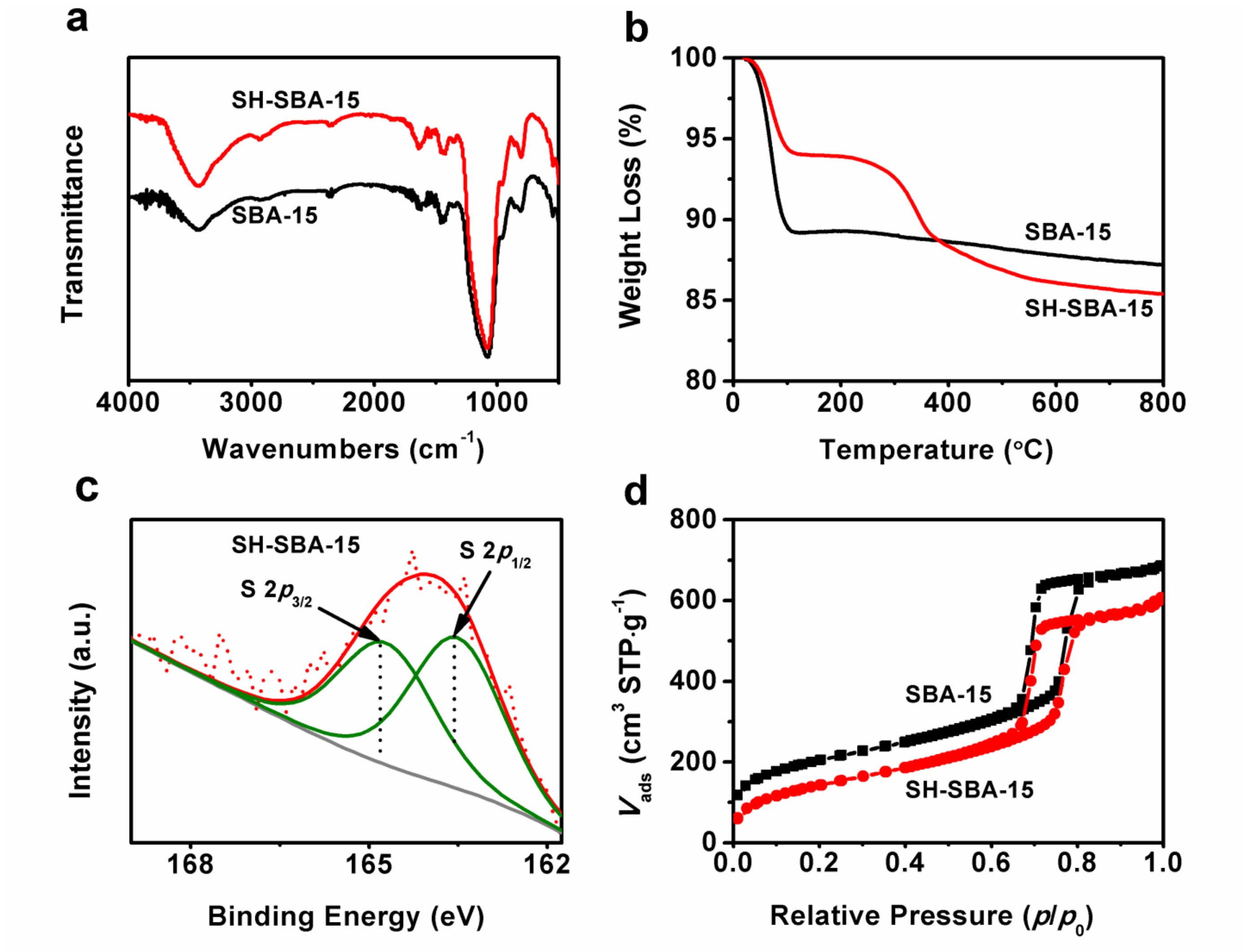

Supplementary Fig. 28. Synthesis and characterization of mercapto-functionalized ordered mesoporous silica SH-SBA-15. **a**, FT-IR spectra for pristine and mercapto-functionalized SH-SBA-15. In comparison to that of pristine SBA-15, the FTIR spectrum of SH-SBA-15 contained several absorbances in the 3000 - 2800 cm^{-1} range, which were assigned to the C-H vibrations in the mercaptopropyl groups. This phenomenon provides further evidence of the modification of the organic functional groups on mesoporous silica SBA-15. However, the mercapto group is IR invisible under the current conditions, which may be due to the weak dipoles of the S-H groups that hinder detection of their modes by vibrational spectroscopy[12]. **b**, TG curves for pristine and mercapto-functionalized SH-SBA-15. The TG curve for pristine SBA-15 revealed a weight loss below 100 ℃, corresponding to the physisorption of water. In comparison, the mercapto-functionalized SBA-15 exhibited the second distinct weight loss between 250 and 500 ℃, which may be due to the loss of grafted mercaptopropyl groups on the mesoporous silica. The S content was estimated to be approximately 2.5 mmol S g^{-1} solid, which is consistent with the value obtained by elemental analysis. **c**, XPS spectrum for SH-SBA-15 in the S 2*p* region, showing one strong peak corresponding to the –SH group. **d**, N_2 sorption isotherms for pristine and mercapto-functionalized SH-SBA-15. Typical type IV curves can be observed for both samples, suggesting uniform mesopores. After modification of the mercaptopropyl groups, the BET surface area and pore volume decreased from 706 to 510 m^2 g^{-1}, and from 1.04 to 0.78 cm g^{-1}, respectively, and the pore size remained at 8.9 nm. The results are similar to those of mesoporous silica modified with organic moieties and reflects grafting of the moieties inside the pores[13].

Mercapto-group functionalized mesoporous silica SBA-15 (SH-SBA-15) was synthesized by grafting mercaptopropyl groups on pre-prepared mesoporous silica SBA-15[13,14]. Pure mesoporous silica SBA-15 was synthesized by the hydrothermal method. The initial solutions contained 2.08 g of TEOS, 1.0 g of P123, 30 g of HCl (2 M), and 7.5 g of H_2O[15]. The hydrothermal temperature and time were 100 ℃ and 24 h. After surfactant removal at 550 ℃ under air, the mesoporous silica SBA-15 carrier was obtained. A toluene (120 mL) suspension of SBA-15 (4.1 g) was then mixed with 10.0 g of 3-thiolpropyltrimethoxysilane at reflux temperature for 48 h; 1.8 mL of water was added to promote cross-linking, and the mixture was heated at reflux for an additional 24 h. The solids were then filtered and washed with copious amounts of toluene, hexanes, and methanol to remove unreacted silanes. The recovered solids were Soxhlet extracted with dichloromethane at reflux temperature for 24 h. The resulting white solids were collected, dried at room temperature overnight and subsequently at 150 ℃ for 3 h under vacuum, and stored in a vacuum dryer.

Supplementary Table 1. Metal concentration ratios calculated from XRD patterns using the Vegard's law and XPS spectra for confined Au-Pd nanoalloy catalysts.

Sample	Au:Pd (atomic ratio)		
	Theo.[a]	XRD[b]	XPS[c]
$Au_{80}Pd_{20}$	4	4.20	4.18
$Au_{67}Pd_{33}$	2	2.36	1.80
$Au_{50}Pd_{50}$	1	2.08	0.90
$Au_{33}Pd_{67}$	0.5	1.17	0.42
$Au_{20}Pd_{80}$	0.25	0.93	0.21
$Au_{80}Pd_{20}$-R1	4	3.93	3.88
$Au_{67}Pd_{33}$-R1	2	2.61	1.82
$Au_{50}Pd_{50}$-R1	1	2.19	0.98
$Au_{33}Pd_{67}$-R1	0.5	1.31	0.44
$Au_{20}Pd_{80}$-R1	0.25	0.96	0.18

[a] theoretical Au:Pd atomic ratio;

[b] Au:Pd atomic ratio estimated by the lattice spacing calculated from the XRD patterns using the Vegard's law[16];

[c] Au:Pd atomic ratio estimated from the XPS spectra. A distinct overlap could be observed between the Pd 3$d_{5/2}$ and the Au 4$d_{5/2}$ components for the bimetallic catalysts. The Au 4$d_{5/2}$ intensity was calculated from the well-resolved Au 4$f_{7/2}$ intensity, and this value was subtracted from the above overlapped peak to determine the Pd 3$d_{5/2}$ intensity. The resulting value was used to calculate the Au:Pd ratio.

Supplementary Table 2. The parameters of Au(111), Pd(111) and $Au_{50}Pd_{50}(111)$ slab models.[a]

Surface[b]	Unit cell	Number of layer		k-point
		Relaxed	Fixed	
Au(111)	p (4×4)	2	2	3×3×1
Pd(111)	p (4×4)	2	2	3×3×1
$Au_{50}Pd_{50}(111)$	p (4×4)	2	2	3×3×1

[a] vacuum spacing of 15 Å was set to reduce the interaction between repeating slabs.

[b] computational details: All periodic spin-polarized density functional theory (DFT) calculations were carried out using the Vienna Ab-initio Simulation Package (VASP)[17-20]. The interaction between ion cores and valence electrons was described by the projector-augmented wave (PAW) method[21], and the exchange-correlation functional was GGA-PBE[22,23]. The solution of the Kohn-Sham equations was expanded in a plane wave basis set with a cutoff energy of 450 eV. The Brillouin zone sampling was performed using a Monkhorst-Pack grid[24], and the electronic occupancies were determined in light of a Methfessel-Paxton scheme with an energy smearing of 0.2 eV[25]. In all the calculations, a force-based conjugated gradient method was used to optimize the geometries[26]. Saddle points and minima were considered to be converged when the maximum force in each degree of freedom was less than 0.03 eV $Å^{-1}$. The optimized lattice parameter of Pd and Au is 3.956 and 4.173 Å, respectively, which is similar to the values from experiments and other DFT calculations[27,28].

To investigate the adsorption configurations over monometallic and alloy catalysts, typical terraced (111) surfaces for Au(111), Pd(111), and $Au_{50}Pd_{50}(111)$ were used as the slab models.

The adsorption energy of the adsorbate species, E_{ads}, was calculated with the formula:

$$E_{ads} = E_{adsorbate + slab} - (E_{adsorbate} + E_{slab}) \quad (10)$$

where $E_{adsorbate + slab}$ is the total energy of the relaxed adsorbate-surface system, while E_{slab} and $E_{adsorbate}$ are the total energy of the relaxed bare surface and gas phase adsorbate, respectively[29]. Hence, the adsorption energy was defined as negative if the total energy decreased when the adsorbate was brought from infinity and placed onto the surface.

Supplementary Table 3. The values (in units of eV) of the measured core-level binding energy shift $\Delta E(i)$, the work function change $\Delta\Phi$, the final-state relaxation energy shift ΔE_r, the change of the one-electron energy of the core-level i $\Delta\epsilon(i)$ for the (a) Au $4f_{7/2}$ and (b) Pd $3d_{5/2}$ core levels and charge transfer at the (a) Au and (b) Pd sites in AuPd alloys.[a] The binding energy shifts of Au $4f_{7/2}$ and Pd $3d_{5/2}$ core-level values are relative to monometallic gold and palladium catalysts.

(a) Au $4f_{7/2}$ level

Alloy composition	$\Delta E(i)$ (eV)	$\Delta\Phi$ (eV)	ΔE_r (eV)	Δn_d (e)	δn^{Au} (e)	Δn_s (e)
$Au_{80}Pd_{20}$	-0.058	0.060	0.10	0.045	-0.0928	-0.1378
$Au_{67}Pd_{33}$	-0.208	0.098	0.19	0.028	-0.0639	-0.0919
$Au_{50}Pd_{50}$	-0.218	0.150	0.24	-0.115	0.0769	0.1919
$Au_{33}Pd_{67}$	-0.218	0.201	0.31	-0.124	0.0430	0.1670
$Au_{20}Pd_{80}$	-0.258	0.245	0.33	-0.162	0.0806	0.2426

(b) Pd $3d_{5/2}$ level

Alloy composition	$\Delta E(i)$ (eV)	$\Delta\Phi$ (eV)	ΔE_r (eV)	Δn_d (e)	δn^{Pd} (e)	Δn_s (e)
$Au_{80}Pd_{20}$	-0.39	-0.239	-1.63	0.2488	0.3711	0.1223
$Au_{67}Pd_{33}$	-0.38	-0.2	-1.31	0.2861	0.1277	-0.1602
$Au_{50}Pd_{50}$	-0.30	-0.15	-1.84	0.2826	-0.0769	-0.3595
$Au_{33}Pd_{67}$	-0.17	-0.099	-0.58	0.1638	-0.0215	-0.1853
$Au_{20}Pd_{80}$	-0.04	-0.059	-0.33	0.0868	-0.0201	-0.1069

[a] Calculation: Experimental binding energy shift $\Delta E(i)$ of the core level i between pure metal and the alloy measured relative to the Fermi level in XPS experiments can be written as follows[30,31]:

$$\Delta E(i) = E_B(i, \text{alloy}) - E_B(i, \text{metal}) = -\Delta\epsilon(i) + \Delta E_F - \Delta E_r \quad (11)$$

where $\Delta\epsilon(i)$ is the change of the Hartree-Fock one-electron energy of the core level i, ΔE_F is the change of the Fermi energy, and ΔE_r is the change of relaxation energy in the presence of the core hole between pure metal and alloy.

The change of the Hartree-Fock one-electron energy of the core level $\Delta\epsilon(i)$, which is directly related to the charge transfer in the initial ground state, can be written as

$$\Delta\epsilon(i) = \sum_j^{\text{val}} \left[\Delta n_j F^0(i, j)\right] - \left[\sum_j^{\text{val}} \Delta n_j\right] F^0_{\text{latt}} \quad (12)$$

$$\delta n = \sum_j^{\text{val}} \Delta n_j \quad (13)$$

where $F^0(i, j)$ is the screened Coulomb integrals between the core electron i and the valence electron j, and Δn_j is the change in the number of the valence electron j. The following values for the Coulomb integrals in the solids are used: $F^0(3d, 4d)$ = 18.4 eV and $F^0(3d, 4s)$ = 12.9 eV for solid Pd[32], and $F^0(4f, 5d)$ = 16.1 eV and $F^0(4f, 6s)$ = 12.8 eV for solid Au[33]. F^0_{latt} is a Madelung-like potential energy associated with the total charge transfer in the surrounding host atoms. Using the metallic radii of Pd and Au (r_{Pd} = 0.138 nm and r_{Au} = 0.144 nm)[28], $F^0_{\text{latt}}(e^2/r_{Pd})$ = 10.5 eV for the Pd atom and $F^0_{\text{latt}}(e^2/r_{Au})$ = 10.1 eV for the Au atom is obtained.

The first term in the right-hand side of Equation (12) arises from the redistribution of valence electrons, and the second term is associated with the charge changes on other lattice sites due to the total charge transfer in or out of the parent atomic site. In the case of Au (or Pd) atom, the Equation (12) can be rewritten as:

$$\Delta\epsilon(i) = \Delta n_d F^0(i, d) + \Delta n_s F^0(i, s) - \delta n F^0_{\mathrm{latt}} \tag{14}$$

$$\delta n = \Delta n_d + \Delta n_s \tag{15}$$

We first assumed that $\Delta E_F = -\Delta\Phi$, the difference of the experimental work functions of the metal and the alloy. This is equivalent to assuming that the surface dipole barrier potential contribution to the work function does not change between pure metal and the alloy. The term ΔE_r is the difference of the relaxation energy in the presence of the core hole between alloy and metal, so it is related to the final-state screening effect. A the oretical calculation[34] on alloy systems based on the pseudopotential linear response method in the presence of the core hole revealed that ΔE_r could be as large as 2 eV, which was the same order of magnitude as the initial-state effect $\Delta\epsilon(i)$ and therefore could not be neglected. Here the result of this calculation in Ref. 32 was used. The following relation between the experimental binding energy shift $\Delta E(i)$ and the amount of the charge transfer could be obtained:

$$\begin{aligned} \Delta E(i) + \Delta\Phi + \Delta E_r &= -\Delta\epsilon(i) \\ &= -\Delta n_d \left[F^0(i, d) - F^0(i, s)\right] + \delta n\left[F^0_{\mathrm{latt}} - F^0(i, s)\right] \end{aligned} \tag{16}$$

Furthermore, the total charge neutrality condition for the Au_xPd_{1-x} alloy implies

$$(1 - x)\delta n^{\mathrm{Pd}} + x\delta n^{\mathrm{Au}} = 0 \tag{17}$$

δn^{Au} (δn^{Pd}) is the amount of the total interatomic charge transfer into the Au (Pd) atom.

Supplementary Methods

Preparation of phenolic resins. The carbon precursors (low molecular weight, soluble phenolic resins) were prepared from phenol and formaldehyde in a base-catalyzed process. In a typical procedure, 8.0 g of phenol were melted at 42 - 45 ℃ in a flask and mixed with 0.34 g of a 20 wt% aqueous sodium hydroxide (NaOH) solution under stirring. After 10 min, 5.24 g of formalin (37 wt% formaldehyde) were added. Then, the mixture was heated to 70 ℃. After additional stirring for 1 h at this temperature, the mixture was cooled to room temperature. The pH value was adjusted to ~ 7.0 with a 2 M HCl solution. Then, water was removed by vacuum evaporation below 45 ℃. The water- and ethanol-soluble phenolic resins were dissolved in ethanol (20 wt%) for further use.

Supplementary References

1. Sun, K.-Q., Hong, Y.-C., Zhang, G.-R. & Xu, B.-Q. Synergy between Pt and Au in Pt-on-Au nanostructures for chemoselective hydrogenation catalysis. *ACS Catal.* **1**, 1336-1346 (2011).
2. Madon, R. J. & Boudart, M. Experimental criterion for the absence of artifacts in the measurement of rates of heterogeneous catalytic reactions. *Ind. Eng. Chem. Fund.* **21**, 438-447 (1982).
3. Abad, A., Corma, A. & García, H. Catalyst parameters determining activity and selectivity of supported gold nanoparticles for the aerobic oxidation of alcohols: the molecular reaction mechanism. *Chem. Eur. J.* **14**, 212-222 (2008).
4. Fang, W. et al. Hydrotalcite-supported gold catalyst for the oxidant-free dehydrogenation of benzyl alcohol: studies on support and gold size effects. *Chem. Eur. J.* **17**, 1247-1256 (2011).
5. Chen, W. Y. et al. Mechanistic insight into size-dependent activity and durability in Pt/CNT catalyzed hydrolytic dehydrogenation of ammonia borane. *J. Am. Chem. Soc.* **136**, 16736-16739 (2014).
6. Della Pina, C., Falletta, E., Rossi, M. & Sacco, A. Selective deactivation of gold catalyst. *J. Catal.* **263**, 92-97 (2009).
7. Teschner, D. et al. In situ surface coverage analysis of RuO_2-catalysed HCl oxidation reveals the entropic origin of compensation in heterogeneous catalysis. *Nat. Chem.* **4**, 739-745 (2012).
8. Chen, W. et al. Mechanistic and kinetic insights into the Pt-Ru synergy during hydrogen generation from ammonia borane over PtRu/CNT nanocatalysts. *J. Catal.* **356**, 186-196 (2017).
9. Woehrle, G. H., Warner, M. G. & Hutchison, J. E. Ligand exchange reactions yield subnanometer, thiol-stabilized gold particles with defined optical transitions. *J. Phys. Chem. B* **106**, 9979-9981 (2002).
10. Matsui, T. et al. Theoretical studies on sulfur and metal cation (Cu(II), Ni(II), Pd(II), and Pt(II))-containing artificial DNA. *J. Phys. Chem. B* **113**, 12790-12795 (2009).
11. Rowland, T., Cusack, N. E. & Ross, R. G. The resistivity and thermoelectric power of the palladium-gold alloy system. *J. Phys. F: Met. Phys.* **4**, 2189-2201 (1974).
12. Wilson, K., Lee, A. F., Macquarrie, D. J. & Clark, J. H. Structure and reactivity of sol–gel sulphonic acid silicas. *Appl. Catal., A* **228**, 127-133 (2002).
13. Sakamoto, Y. et al. Synthesis of platinum nanowires in organic-inorganic mesoporous silica templates by photoreduction: formation mechanism and isolation. *J. Phys. Chem. B* **108**, 853-858 (2004).
14. Richardson, J. & Jones, C. Strong evidence of solution-phase catalysis associated with palladium leaching from immobilized thiols during heck and suzuki coupling of aryl iodides, bromides, and chlorides. *J. Catal.* **251**, 80-93 (2007).

15. Zhao, D. et al. Triblock copolymer syntheses of mesoporous silica with periodic 50 to 300 angstrom pores. *Science* **279**, 548-552 (1998).
16. Okamoto, H. & Massalski, T. B. The Au-Pd (gold-palladium) system. *Bull. Alloy Phase Diagrams* **6**, 229-235 (1985).
17. Kresse, G. & Hafner, J. Ab initio molecular dynamics for liquid metals. *Phys. Rev. B* **47**, 558-561 (1993).
18. Kresse, G. & Hafner, J. Ab initio molecular-dynamics simulation of the liquid-metal-amorphous-semiconductor transition in germanium. *Phys. Rev. B* **49**, 14251-14269 (1994).
19. Kresse, G. & Furthmüller, J. Efficiency of ab-initio total energy calculations for metals and semiconductors using a plane-wave basis set. *Comp. Mater. Sci.* **6**, 15-50 (1996).
20. Kresse, G. & Furthmüller, J. Efficient iterative schemes for ab initio total-energy calculations using a plane-wave basis set. *Phys. Rev. B* **54**, 11169-11186 (1996).
21. Blöchl, P. E. Projector augmented-wave method. *Phys. Rev. B* **50**, 17953-17979 (1994).
22. Kresse, G. & Joubert, D. From ultrasoft pseudopotentials to the projector augmented-wave method. *Phys. Rev. B* **59**, 1758-1775 (1999).
23. Perdew, J. P., Burke, K. & Ernzerhof, M. Generalized gradient approximation made simple. *Phys. Rev. Lett.* **77**, 3865-3868 (1996).
24. Monkhorst, H. J. & Pack, J. D. Special points for brillouin-zone integrations. *Phys. Rev. B* **13**, 5188-5192 (1976).
25. Methfessel, M. & Paxton, A. T. High-precision sampling for brillouin-zone integration in metals. *Phys. Rev. B* **40**, 3616-3621 (1989).
26. Sheppard, D., Terrell, R. & Henkelman, G. Optimization methods for finding minimum energy paths. *J. Chem. Phys.* **128**, 134106 (2008).
27. Rankin, R. B. & Greeley, J. Trends in selective hydrogen peroxide production on transition metal surfaces from first principles. *ACS Catal.* **2**, 2664-2672 (2012).
28. Kittel, C. *Introduction to solid state physics*, 6th edn. Wiley (1986).
29. Alfonso, D. R., Cugini, A. V. & Sorescu, D. C. Adsorption and decomposition of H_2S on Pd(111) surface: a first-principles study. *Catal. Today* **99**, 315-322 (2005).
30. Choi, E., Oh, S. J. & Choi, M. Charge transfer in Ni_xPt_{1-X} alloys studied by X-ray photoelectron spectroscopy. *Phys. Rev. B* **43**, 6360-6368 (1991).
31. Watson, R. E. & Perlman, M. L. X-ray photoelectron spectroscopy application to metals and alloys. Springer Berlin Heidelberg (1975).
32. Cho, E.-J. et al. Unoccupied states and charge transfer in Cu-Pd alloys studied by bremsstrahlung isochromat

spectroscopy, X-ray photoelectron spectroscopy, and L_{III} absorption spectroscopy. *Phys. Rev. B* **52**, 16443-16450 (1995).

33. Gelatt, C. D. & Ehrenreich, H. Charge transfer in alloys: AgAu. *Phys. Rev. B* **10,** 398-415 (1974).
34. Castellani, N. J. & Leroy, D. B. Final state core level binding energy shifts in binary alloys. *Z. Phys. B - Condensed Matter* **71**, 315-319 (1988).
