# Supplementary material for: Optimising surface d charge of AuPd nanoalloy catalysts for enhanced catalytic activity
Source: Nat Commun. 2019 Mar 29;10:1428. doi: 10.1038/s41467-019-09421-5 (PMC6441046; doi:10.1038/s41467-019-09421-5)
Supplement: Supplementary file 2 — Peer Review [file 41467_2019_9421_MOESM2_ESM.pdf]

Reviewers' comments:

Reviewer #1 (Remarks to the Author):

While the discussion on the alloy structure is interesting I have concerns on the catalysis data.
First it is surprising that no toluene is observed or the ester.

Second the Pd only catalyst is probably making CO2 and this is not being looked for. The reactivity switches off for the Pd catalyst which may be due to O2 depletion and CO2 formation.

Why is work carried out in water when benzyl alcohol oxidation is best done solvent free.
Why are there no data for Au only. It should have some actvity.

Hence the characterisation looks very good but the catalysis aspect is not at the required level.

Reviewer #2 (Remarks to the Author):

In this study, the authors synthesized monodispersed AuPd catalysts with continuous change of the Pd-Au coordination numbers embedded in a relatively inert mesoporous carbonaceous matrix. Such a careful way of creating their catalysts allows some unambiguous conclusions to be made between d band properties of the Pd component of their alloy catalysts and the catalytic performance. Overall this is a high quality study. I have only one question for the authors. In this study, turnover frequencies were normalized to surface Pd concentrations measured ex situ. In order to make their normalized reaction rates better justified, it is suggested that they also perform a surface Pd concentration analysis of the used catalysts. If Pd surface concentrations before and after catalysis are similar, then the current TOFs are regarded reasonable. However if surface Pd concentrations before and after reaction differs substantially, the authors may need to recalculate their TOFs.

Reviewer #3 (Remarks to the Author):

This manuscript describes an interesting, though sophisticated method, for the synthesis of 2-3 nm sized Pd-Au bimetallic nanoparticles with tunable compositions. It is surprising that Au-containing nanoparticles are so stable even survive after reduction at 600C. Maybe the confinement effect of silica-carbon hybrid structure plays some role in stabilizing these nanoparticles. I suggest the authors to discuss the origin of the high stability.

TEM is not great in characterizing subnanometer metal clusters. Since they are capable of doing STEM, some efforts should be devoted on STEM-HAADF observation and the images should be provided. They mentioned that their EDX data were collected under STEM mode on at least 20 particles. I suggest them to list the individual composition of each particle so that the readers will have some idea about their composition distribution.

It seems that both Pd and Au in the Pd-Au bimetallic catalysts shift to lower binding energies compared to pure Pd and Au. What causes such shift? If there is an electron flow from one metal to another, their shifts in binding energy should be in opposite directions.

It is not clear how they calculated TOF. How do they quantify surface Au and Pd? Counting surface sites is crucial for TOF calculation in order to draw any quantitative conclusions.

Gold has been shown as very active catalyst for alcohol oxidations. Maybe the presence of base is crucial. At least they should present their catalytic data on pure Au catalyst synthesized in their work.

Responses to Reviewers' comments

Reviewer #1

Comment. While the discussion on the alloy structure is interesting I have concerns on the catalysis data. The characterisation looks very good but the catalysis aspect is not at the required level.

Response. Thank you very much for your positive comment. This manuscript majorly focuses on the direct determination of the structure-property relationship for a heterogeneous catalyst. Therefore, following your suggestion, we have supplied more detailed data to strengthen the catalysis aspect. All these data strongly support the finding that the experimentally measurable *d* charge on the surface of Pd correlates well with the catalytic activities and serves as a descriptor to the adsorbate states and hence the catalytic oxidation performance. The detailed responses are listed below.

Comment 1. First it is surprising that no toluene is observed or the ester.

Response 1. Thank you very much for this important comment, which helps us to give a much clearer explanation of the catalysis process. We have re-checked the products and confirmed that undetectable toluene and benzyl benzoate are formed in our process.

As you kindly pointed out, in the low temperature liquid-phase oxidation of benzyl alcohol, many products including toluene, benzoic acid, benzyl benzoate, and dibenzyl ether have been reported to be formed besides the main product benzaldehyde. Benzaldehyde is formed by the sequential oxidative dehydrogenation of benzyl alcohol, while further oxidation produces benzoic acid. Benzyl ether is produced by the dehydration of benzyl alcohol[1,2], and benzyl benzoate is formed by either of two pathways: the esterification of benzoic acid by benzyl alcohol, or *via* hemi-acetal from benzaldehyde[2,3]. Toluene is expected to be formed by the hydrogenolysis of benzyl alcohol, or disproportionation of benzyl alcohol[4]. The formation of the byproducts is very sensitive to the reaction conditions and catalyst used. For example, toluene is one of the main byproducts in the solvent-free oxidation of benzyl alcohol at a relatively high temperature (>120 °C)[4,5], but is seldom detected in the water-mediated reaction[3]. Benzyl benzoate has been found as a byproduct in the presence of base which favors the formation of hemi-acetal from benzaldehyde over activated carbon supported gold catalysts[6].

We have carefully re-checked the products by GC-MS and can confirm that undetectable toluene and benzyl benzoate are formed. The undetectable toluene and benzyl benzoate for the base-free oxidation of benzyl alcohol in water has also been reported by Prati and co-workers[4]. The inhibition of toluene and benzyl benzoate is

possibly related to the base-free oxidation in water under atmosphere pressure over carbon supported AuPd nanoparticles.

References

1. Sankar, M. et al. *Chem. Eur. J.* **17,** 6524-6532 (2011).
2. Evangelisti, C. et al. *J. Catal.* **286,** 224-236 (2012).
3. Villa, A. et al. *Appl. Catal. A* **364,** 221-228 (2009).
4. Cao, E. et al. *Catal. Today* **203,** 146-152 (2013).
5. Enache, D. I. et al. *Science* **311,** 362-365 (2006).
6. Wang, S. et al. *ACS Catal.* **5,** 797-802 (2015).

Accordingly, we have revised our manuscript.

Revised manuscript, Page 13, several sentences have been added:

"Other byproducts such as toluene and benzyl benzoate are undetected, analogous to the results over supported AuPd nanocatalysts reported by Prati and co-workers[41]. The formation of the byproducts is very sensitive to the reaction conditions and catalyst used. For example, toluene is one of the main byproducts in the solvent-free oxidation of BA at a relatively high temperature (>120 °C) possibly *via* the disproportionation of BA[1], but is seldom detected in the water-mediated reaction[41]. Therefore, the inhibition of these byproducts is possibly related to the base-free oxidation in water under atmosphere pressure over carbon supported AuPd nanoparticles."

Comment 2. Second the Pd only catalyst is probably making CO_2 and this is not being looked for. The reactivity switches off for the Pd catalyst which may be due to O_2 depletion and CO_2 formation.

Response 2. This comment is highly appreciated. With your kind suggestion, we have been trying to confirm if complete oxidation of benzyl alcohol to CO_2 occurs and O_2 depletes over the Pd-only catalyst. After reaction, the gas phase was analysed to determine the concentrations of O_2, CO and CO_2 by GC (7890A) using a porapak Q column. The CO_2 concentration is under the detection line, and only oxygen can be detected. In addition, when oxygen flow was carefully injected into the reaction liquid, the conversion of benzyl alcohol over the Pd only catalyst almost remains unchanged. Therefore, the switching-off for the reactivity of the Pd only catalyst is not due to the O_2 depletion and CO_2 formation, but due to the strong adsorption of produced aldehyde and acid on the surface[1].

References

1. Keresszegi, C., Ferri, D., Mallat, T. & Baiker, A. *J. Phys. Chem. B* **109,** 958-967 (2005).

Accordingly, we have revised our manuscript.

Revised manuscript, Page 10:

"The neat Pd catalyst results in low conversion and fast deactivation possibly due to the strong adsorption of produced aldehyde and acid on the surface." has been changed to "The neat Pd catalyst results in a low conversion and fast deactivation. The complete oxidation of benzyl alcohol to CO_2 and the depletion of O_2 can be excluded. Therefore, the switching-off for the neat Pd catalyst is possibly due to the strong adsorption of produced aldehyde and acid on the surface"

Revised manuscript, Page 21, a sentence has been added:

"The gas phase was analysed to determine the concentrations of O_2, CO and CO_2 by GC (7890A) using a porapak Q column."

Comment 3. Why is work carried out in water when benzyl alcohol oxidation is best done solvent free?

Response 3. Thanks so much for your valuable comment. As kindly pointed out by you, the TOF values for the Au, Pd and AuPd catalysts can reach very high for the benzyl alcohol oxidation under solvent-free conditions[1,2]. By comparison, the TOF values are relatively low using water or toluene as the solvent[3-5]. This manuscript majorly focuses on the direct determination of the structure-property relationship for a heterogeneous catalyst. Therefore, the model oxidation reactions were carried out under atmosphere pressure in water. In this case, both the adsorbate states of reactant and the catalytic oxidation performance are highly related to the *d*-orbital charge on the surface of Pd.

References

1. Enache, D. I. et al. *Science* **311,** 362-365 (2006).
2. Dimitratos, N. et al. *Catal. Today* **122,** 317-324 (2007).
3. Dimitratos, N. et al. *J. Catal.* **244,** 113-121 (2006).
4. Prati, L. et al. *Faraday Discuss.* **152,** 353-365 (2011).
5. Wang, H., et al. *Appl. Catal. A* **536,** 27-34 (2017).

Accordingly, we have revised our manuscript.

Revised manuscript, Page 10:

"The aerobic oxidation of primary alcohols in an aqueous solution in the absence of base is used as a model reaction here because this is a strong adsorbate-involved reaction and Pd-involving sites are more active than the Au case on a carbon support[40]."

Comment 4. Why are there no data for Au only? It should have some activity.

Response 4. Many thanks for the insightful comment. The activity for the studied reaction of the Au only catalyst has been re-checked. Undetectable reactant conversion has been observed.

Different studies agree with that the oxidation of benzyl alcohol proceeds through a benzyl alkoxide ($PhCH_2O$-) intermediate to adsorbed benzaldehyde. The bond scission sequence is O-H followed by C^{α}-H. The DFT result shows that the chemical bonding between the Au(111) surface and the functional group of hydroxyl and phenyl group is negligible. Therefore, the Au surface is not able to extract the hydride from the alcoholic function.

It should be mentioned that both the reaction conditions and carbon support can influence the overall activity of gold catalysts. The activities differ in literatures[1-5]. Prati and co-workers used activated carbon as the support to load gold nanoparticles[1]. The catalyst behaves inert in the water-mediated benzyl alcohol oxidation under the oxygen pressure at 0.2 MPa due to the fact that it is not able to extract the hydride from the alcoholic function. Hutchings and co-workers reported that the Au/C catalyst shows an extremely low conversion at 100 °C under 0.2 MPa in the solvent-free oxidation of benzyl alcohol (2% of 3 h)[2]. Later on, they synthesized an Au/C catalyst over which the conversion of benzyl alcohol can reach 48% after 6 h at an elevated temperature of 160 °C and pressure of 1.0 MPa [3].

The surface chemistry and crystal structure of carbon supports also show distinct influence on the activity for gold nanocatalysts, including the surface basic groups, surface oxygenated acid groups, and the crystallinity of carbon supports[6-11]. For example, Davis and co-workers found that the delocalized electrons in basic carbon supports lead to high electronic mobility and enhance the formation/bonding of hydroxide, which is an important intermediate on Au surface during the liquid phase alcohol oxidation[7]. Rodrigues *et al.* also found that activated carbons with basic oxygen-free groups characterized by a high density of free π-electrons enhance the activity in glycerol oxidation, while a high content of acid groups leads to poor performance[8]. In addition, Gil *et al.* proposed that high crystalline materials, such as graphite and ribbon type CNF with a low number of structural defects lead to small, thin and faceted Au particles strongly anchored to the orderly exposed graphite edges which promote Au–C interaction and facilitate the proton abstraction from glycerol[9]. Huang and co-workers highlighted the positive role for both surface chemistry of carbon and graphitic character to improve the activity in oxidation of benzyl alcohol over carbon supported gold catalysts[10]. These findings demonstrate that the carbon supports with high crystallinity and groups characterized by a high density of free π-electrons may show interaction with gold

nanoparticles and modify the electronic properties, which facilitate the proton abstraction from alcohols. As a result, they would show activity in oxidation of benzyl alcohol.

By comparison, the present carbon support is derived from phenolic resins which possess abundant surface oxygenated acid groups and extremely low graphitic degree[12]. In this case, the interaction between the carbon support and gold nanoparticles is weak and shows an indistinct influence on the electronic structure of the gold nanoparticles. In addition, the oxidation in the present study is proceeded under atmosphere pressure. As a result, the gold nanoparticles supported on less-active porous carbons, as shown in this study, behave inert in benzyl alcohol oxidation.

References

1. Dimitratos, N. et al. *J. Catal.* **244,** 113-121 (2006).
2. Hutchings, G. J. et al. *Top. Catal.* **38,** 223-230 (2006).
3. Dimitratos, N. et al. *Catal. Today* **122,** 317-324 (2007).
4. Prati, L. et al. *Faraday Discuss.* **152,** 353-365 (2011).
5. Prati, L., Villa, A., Lupini, A. R. & Veith, G. M. *Phys. Chem. Chem. Phys.* **14,** 2969-2978 (2012).
6. Bianchi, C. L., Biella, S., Gervasini, A., Prati, L. & Rossi, M. *Catal. Lett.* **85,** 91-96 (2003).
7. Zope, B. N. Hibbitts, D. D., Neurock, M. & Davis. R. J. *Science* **330,** 74-78 (2010).

8 Rodrigues, E. G., Pereira, M. F. R., Chen, X., Delgado, J. J. & Órfão, J. J. M. *J. Catal.* **281,** 119-127 (2011)

9. Gil, S. Muñoz, L., Sánchez-Silva, L., Romero, A. & Valverde, J. L. *Chem. Eng. J.* **172,** 418-429 (2011).
10. Yu, X. et al. *Appl. Surf. Sci.* **280,** 450-455 (2013).
11. Donoeva, B., Masoud, N. & de Jongh, P. E. *ACS Catal* **7,** 4581-4591 (2017).
12. Fulvio, P. F. et al. *Adv. Funct. Mater.* **21,** 2208-2215 (2011).

Accordingly, we have revised our manuscript.

Revised manuscript, Page 10, several sentences have been added:

"Our results have also shown the content of products generated in the Au-catalysed oxidation of the selected primary alcohols is below the limit of detection, similar to the gold sols loaded on activated carbon catalyst[41]. It should be mentioned that the studied phenolic-resin-derived carbon support has a low graphitic degree[42], and serves as a less-active support, and the gold nanoparticles are not able to extract the hydride from the alcoholic function. Once the base is present, the alcoholate can be formed in solution, and the oxidation activity can be significantly improved[41,43]. In contrast, porous carbon supports with high graphitic degree and basic oxygen-free groups characterized by a high density of free π-electrons, may modify the electronic structure of gold nanoparticles, facilitate the proton abstraction from alcohols, and display conversions of alcohols[44,45]."

Reviewer #2

Comment. In this study, the authors synthesized monodispersed AuPd catalysts with continuous change of the Pd-Au coordination numbers embedded in a relatively inert mesoporous carbonaceous matrix. Such a careful way of creating their catalysts allows some unambiguous conclusions to be made between d band properties of the Pd component of their alloy catalysts and the catalytic performance. Overall this is a high quality study. I have only one question for the authors.

Response. We appreciate very much the positive comment, and are delighted that the reviewer kindly highlights the most important point of our work and gives a very nice comment as "unambiguous conclusions to be made between *d* band properties of the Pd component of their alloy catalysts and the catalytic performance".

Comment 1. In this study, turnover frequencies were normalized to surface Pd concentrations measured ex situ. In order to make their normalized reaction rates better justified, it is suggested that they also perform a surface Pd concentration analysis of the used catalysts. If Pd surface concentrations before and after catalysis are similar, then the current TOFs are regarded reasonable. However if surface Pd concentrations before and after reaction differs substantially, the authors may need to recalculate their TOFs.

Response 1. Thank you very much for your insightful comment. The stable surface Pd species upon reaction is indeed quite important for the heterogenous catalyst. In general, Pd leaching and segregation would lead to surface concentration changes. We have done several experiments to confirm the negligible metal leaching and segregation after reaction and to exclude the change in surface Pd concentration after reaction. The details are shown as follows:

(1) We have supplied the XPS spectra for all AuPd nanoalloy catalysts after reaction. The surface Au:Pd ratio remains almost unchanged after reaction, demonstrating the negligible surface segregation.
(2) The line profiles of the EDX spectra in the STEM mode for the reused catalyst ($Au_{50}Pd_{50}$-R6) is almost the same as that in the fresh catalyst, demonstrating the negligible surface segregation.
(3) The analysis by EDX mapping in the TEM mode displays the metal content remains almost the same after reaction (Au and Pd content of 2.15 and 1.27 *wt*% for the fresh $Au_{50}Pd_{50}$ catalyst, respectively, *v.s.* 2.18 and 1.32 *wt*% for the reused $Au_{50}Pd_{50}$-R6 catalyst), demonstrating the negligible leaching.
(4) A solid containing an unprotected thiol was used to poison the possible soluble metallic species in the oxidation of benzyl alcohol. Results show no significant differences in the TOF values of any of the AuPd nanoalloys in the presence/absence of mercapto-functionalized ordered mesoporous silica (SH-SBA-15). It confirms the negligible Pd leaching into solutions.

Therefore, the surface Pd is stable. Pd surface concentrations before and after catalysis are similar. The TOF values normalized to surface Pd concentrations are therefore convincible.

Accordingly, we have revised our manuscript.

Revised manuscript, Page 12:

"The estimated Au:Pd ratio based on the XPS analysis or the representative EDX line profiles in the STEM mode (Fig. 1) is almost the same as that in the fresh catalyst." has been changed to "The EDX mapping spectra give the Au and Pd concentration of 2.18 and 1.32 *wt*%, respectively, similar to that determined for the fresh catalyst (2.15 and 1.27 *wt*%, respectively), further excluding the leaching of metal into solutions. The estimated Au:Pd ratios based on the XPS analysis, XPD patterns or the representative EDX line profiles in the STEM mode (Fig. 1, Supplementary Table S1 and Fig. S12) are almost the same as that in the fresh catalyst, indicating the surface segregation of Pd or Au can be excluded."

Revised manuscript, Page 13:

"Therefore, this can guarantee the investigation of surface catalysis." has been changed to "Therefore, this can guarantee the investigation of surface catalysis and almost unchanged surface Pd concentrations after reaction."

Revised supplementary information, Page 13, Page 16, XPS spectra and the calculated metal concentration ratios for the catalysts after reaction have been added:

Supplementary Table S1. Metal concentration ratios calculated from XRD patterns using the Vegard's law and XPS spectra for confined Au-Pd nanoalloy catalysts.

Sample	Au:Pd (atomic ratio)		
	Theo[a]	XRD[b]	XPS[c]
$Au_{80}Pd_{20}$	4	4.20	4.18
$Au_{67}Pd_{33}$	2	2.36	1.80
$Au_{50}Pd_{50}$	1	2.08	0.90
$Au_{33}Pd_{67}$	0.5	1.17	0.42
$Au_{20}Pd_{80}$	0.25	0.93	0.21
$Au_{80}Pd_{20}$-R1	4	3.93	3.88
$Au_{67}Pd_{33}$-R1	2	2.61	1.82
$Au_{50}Pd_{50}$-R1	1	2.19	0.98
$Au_{33}Pd_{67}$-R1	0.5	1.31	0.44
$Au_{20}Pd_{80}$-R1	0.25	0.96	0.18

[a] theoretical Au:Pd atomic ratio;

[b] Au:Pd atomic ratio estimated by the lattice spacing calculated from the XRD patterns using the Vegard's law[1];

[c] Au:Pd atomic ratio estimated from the XPS spectra. A distinct overlap could be observed between the Pd $3d_{5/2}$ and the Au $4d_{5/2}$ components for the bimetallic catalysts. The Au $4d_{5/2}$ intensity was calculated from the well-resolved Au $4f_{7/2}$ or Au $4d_{3/2}$ intensity, and this value was subtracted from the above overlapped peak to determine the Pd $3d_{5/2}$ intensity. The resulting value was used to calculate the Au:Pd ratio.

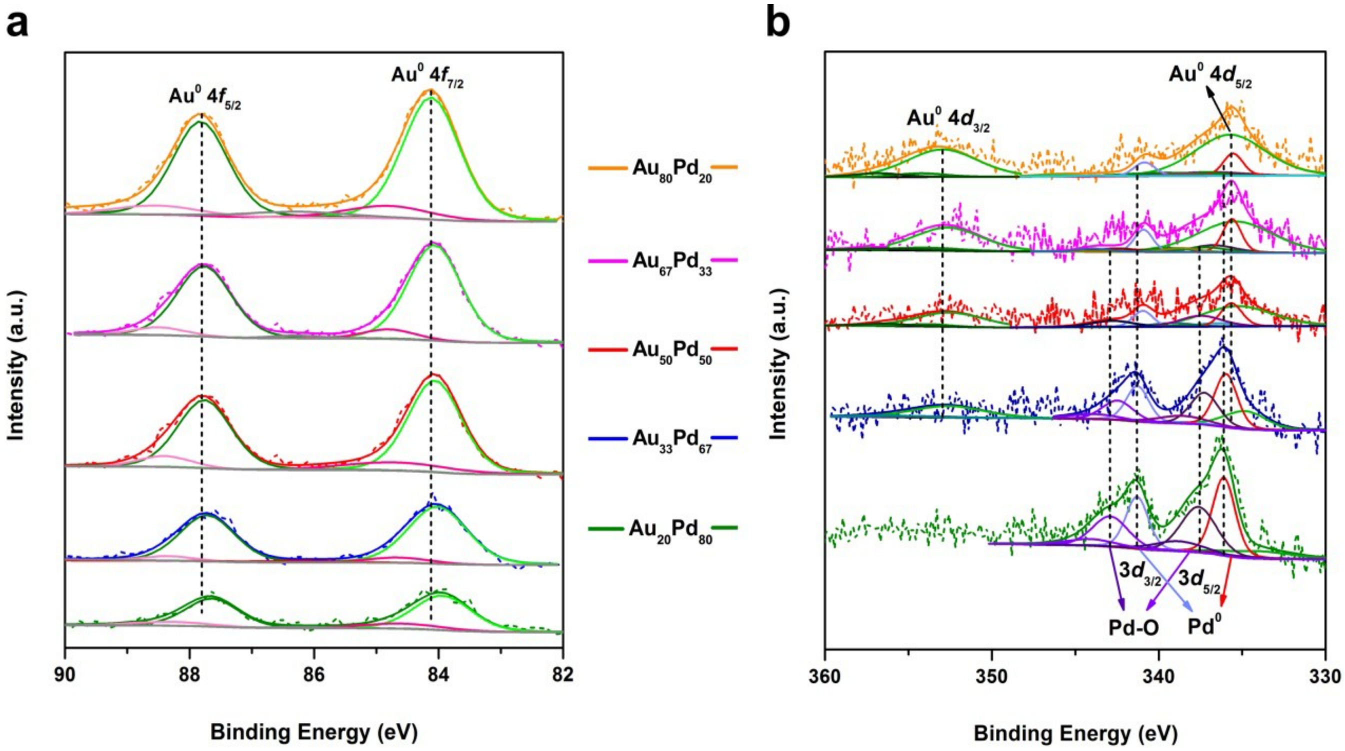

Supplementary Fig. S12. XPS spectra for the AuPd nanocatalysts after reaction. **a**, XPS spectra of the 4*f* level of Au. **b**, XPS spectra of the 3*d* level of Pd. The Au and Pd binding energies were fitted by peak fitting techniques. The original curves were shown as dashed lines, and the fitting curves were shown as solid lines. Metallic Au, metallic Pd and Pd-O, which were the dominant contributors to each spectrum, were discussed. A distinct overlap could be observed between the Pd $3d_{5/2}$ and the Au $4d_{5/2}$ components for the bimetallic catalysts. The Au $4d_{5/2}$ intensity was calculated from the well-resolved Au $4d_{3/2}$ intensity, and this value was subtracted from the above overlapped peak to determine the Pd $3d_{5/2}$ intensity. The resulting value was used to calculate the Au:Pd ratio.

Reviewer #3

Comment. This manuscript describes an interesting, though sophisticated method, for the synthesis of 2-3 nm sized Pd-Au bimetallic nanoparticles with tunable compositions.

Response. Thank you very much for the positive comment.

Comment 1. It is surprising that Au-containing nanoparticles are so stable even survive after reduction at 600 °C. Maybe the confinement effect of silica-carbon hybrid structure plays some role in stabilizing these nanoparticles. I suggest the authors to discuss the origin of the high stability.

Response 1. Thank you very much for the nice suggestive comment. As kindly pointed out by you, the high stability of Au-containing nanoparticles is highly related to the confinement effect of silica-carbon hybrid structure.

The temperature-programmed desorption-mass spectrometry (TPD-MS) measurements (Supplementary Fig. S22) present that the decomposition of resins can occur at elevated temperatures, and the sulfur is released in the form of SO_2 which provides an opportunity for homogeneous mixing of Au and Pd species at atomic level to generate the AuPd nanoalloys. Simultaneously, CO gas is released to create the reducing atmosphere for the *in-situ* reduction of the metal precursors. The break-through of the Au-S and Pd-S bonds at a temperature above 220 °C are accompanied with the reduction of metals and the condensation of the silicate-carbonaceous framework. The neighboured metal species gather together and grow, but the crystal growth energy in the solidified pore walls is high. As a result, the particles are confined by the rigid silica-carbon hybrid framework, homogenously disperse in the matrix, and show high stability even under a reductive atmosphere at 600 °C.

Accordingly, we have revised our manuscript.

Revised manuscript, Page 15, several sentences have been added:

"The break-through of the Au-S and Pd-S bonds at a temperature above 220 °C is accompanied with the reduction of metals and the condensation of the silicate-carbonaceous framework. The neighboured metal species gather together and grow, but the crystal growth energy in the solidified pore walls is high. As a result, the particles are confined by the rigid silica-carbon hybrid framework, homogenously disperse in the matrix, and show high stability even under a reductive atmosphere at 600 °C."

<u>Comment 2</u>. TEM is not great in characterizing sub-nanometer metal clusters. Since they are capable of doing STEM, some efforts should be devoted on STEM-HAADF observation and the images should be provided.

<u>Response 2</u>. Thank you very much for your insightful comment. Your concerns on the contribution of the activity by the sub-nanometer metal clusters are important. Following your suggestion, spherical aberration corrected Transmission Electron Microscope (AC-TEM) images for $Au_{50}Pd_{50}$ were acquired by using a JEM-ARM200F microscope. Uniform nanoparticles with approximate size of 2.7 nm can be observed in large domains and negligible sub-nanometer metal clusters can be found. Thus, the possible activity contribution from the sub-nanometer clusters can be ruled out.

We have accordingly revised the manuscript.

<u>Revised manuscript, Page 5, several sentences have been added and Fig.2 has been changed:</u>

"In addition, the spherical aberration corrected STEM (AC-STEM) images for fresh $Au_{50}Pd_{50}$ show uniform nanoparticles with approximate size of 2.7 nm in large domains and negligible sub-nanometer metal clusters can be found (Fig. 2). Thus, the possibility for the activity contribution from the sub-nanometer clusters can be ruled out."

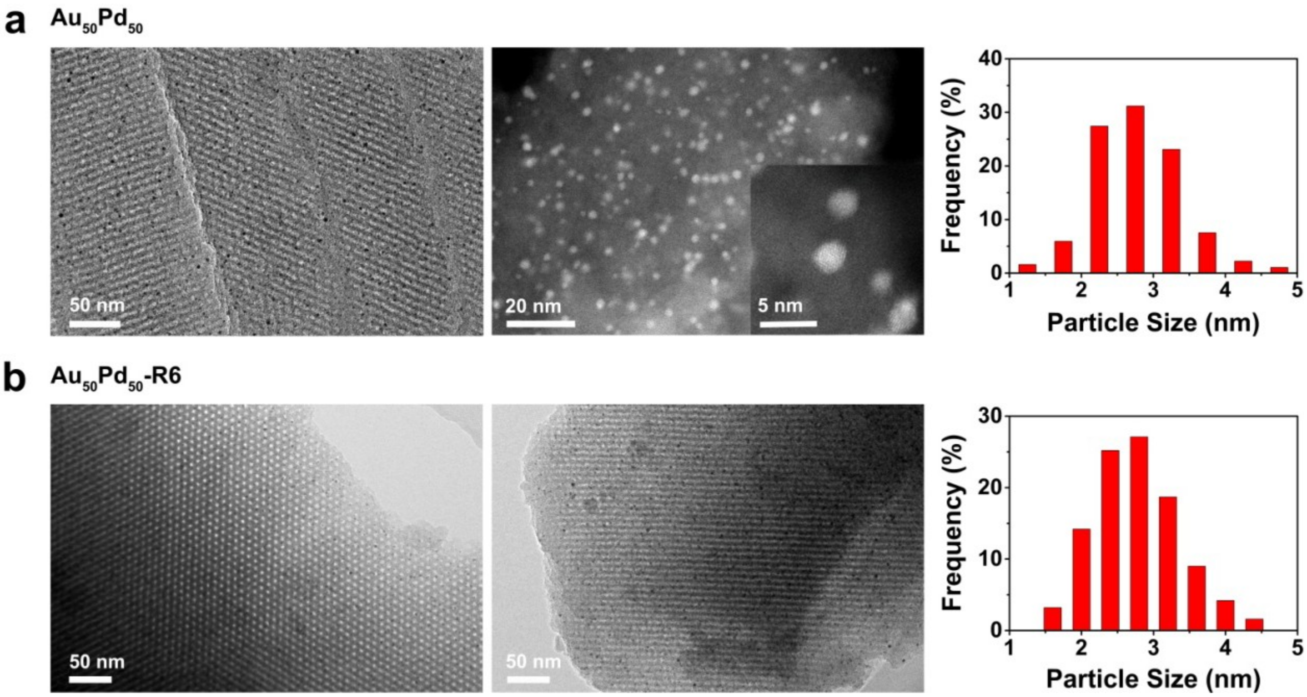

Fig. 2| TEM micrographs. a, Representative TEM image of the ultrathin sections of fresh $Au_{50}Pd_{50}$ and AC-STEM image of the fresh $Au_{50}Pd_{50}$. Inset middle figure is the AC-STEM image with a high magnification. **b**, TEM images of the recycled $Au_{50}Pd_{50}$-R6 after six catalytic runs viewed along the [110] and [001] directions. Particle size distributions were determined with at least 200 nanoparticles.

Revised manuscript, Page 19, a sentence has been added:

"The spherical aberration corrected-STEM (AC-STEM) images were performed using a JEM-ARM 200F microscope operated at 200 kV."

Comment 3. They mentioned that their EDX data were collected under STEM mode on at least 20 particles. I suggest them to list the individual composition of each particle so that the readers will have some idea about their composition distribution.

Response 3. Many thanks for the comment. We have supplied these data, including the STEM image, representative line profiles of the EDX patterns of four single particles, and statics of the individual composition of a single particle in different areas. The composition distributions are uniform for the studied particles and are similar to the bulk material.

Revised supplementary information, Page 4 - 7, four figures (Supplementary Figs. S1 - S4) have been added:

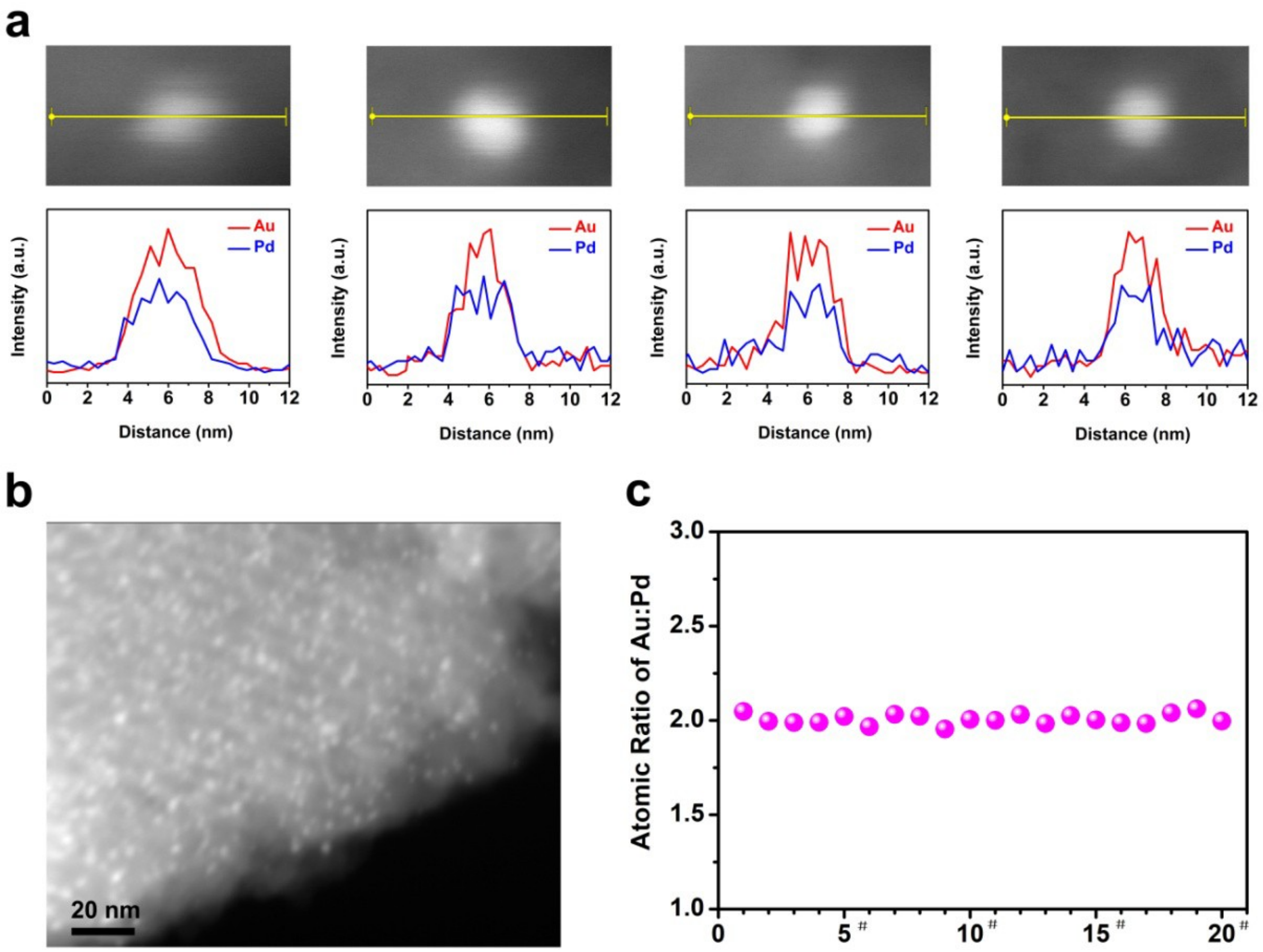

Supplementary Fig. S1. Composition distribution for $Au_{67}Pd_{33}$. **a**, Representative line profiles of the EDX patterns of four single particles collected using a focused electron beam in the sub-nanometre range in the STEM mode. **b**, STEM image. **c**, Statics of the individual composition of a single particle collected in different areas. Each dot represents a nanoparticle.

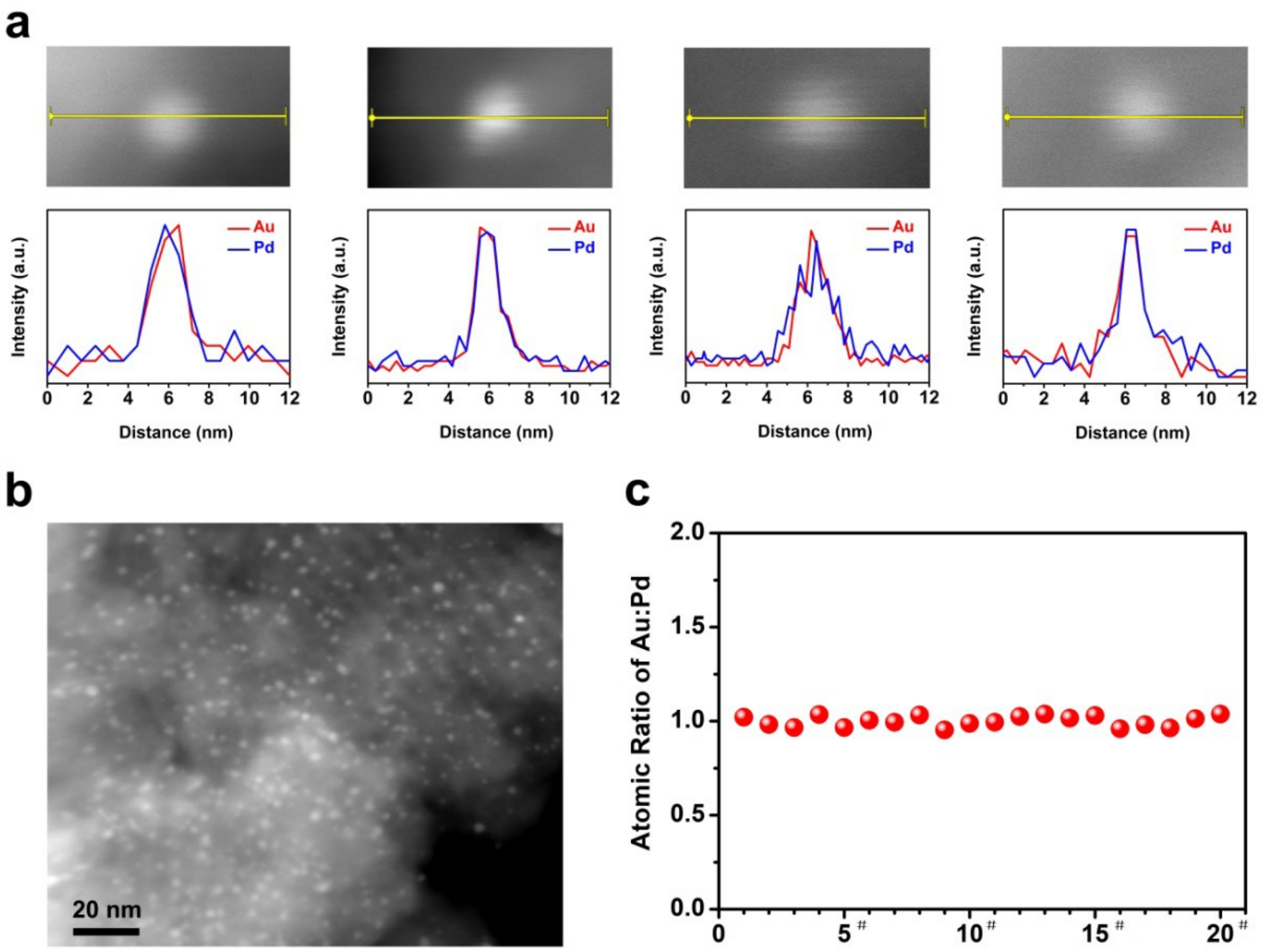

Supplementary Fig. S2. Composition distribution for $Au_{50}Pd_{50}$. **a**, Representative line profiles of the EDX patterns of four single particles collected using a focused electron beam in the sub-nanometre range in the STEM mode. **b**, STEM image. **c**, Statics of the individual composition of a single particle collected in different areas. Each dot represents a nanoparticle.

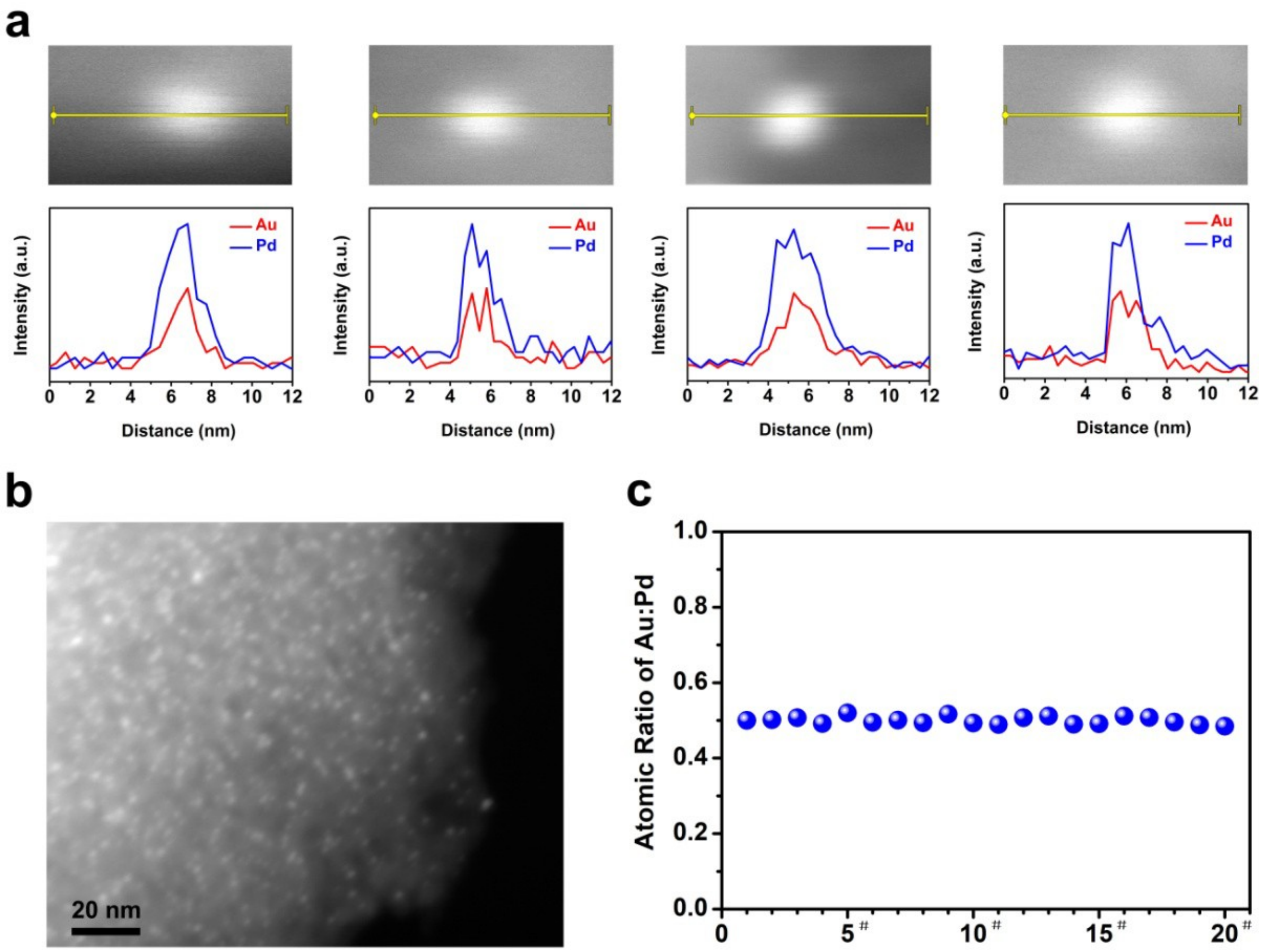

Supplementary Fig. S3. Composition distribution for $Au_{33}Pd_{67}$. **a**, Representative line profiles of the EDX patterns of four single particles collected using a focused electron beam in the sub-nanometre range in the STEM mode. **b**, STEM image. **c**, Statics of the individual composition of a single particle collected in different areas. Each dot represents a nanoparticle.

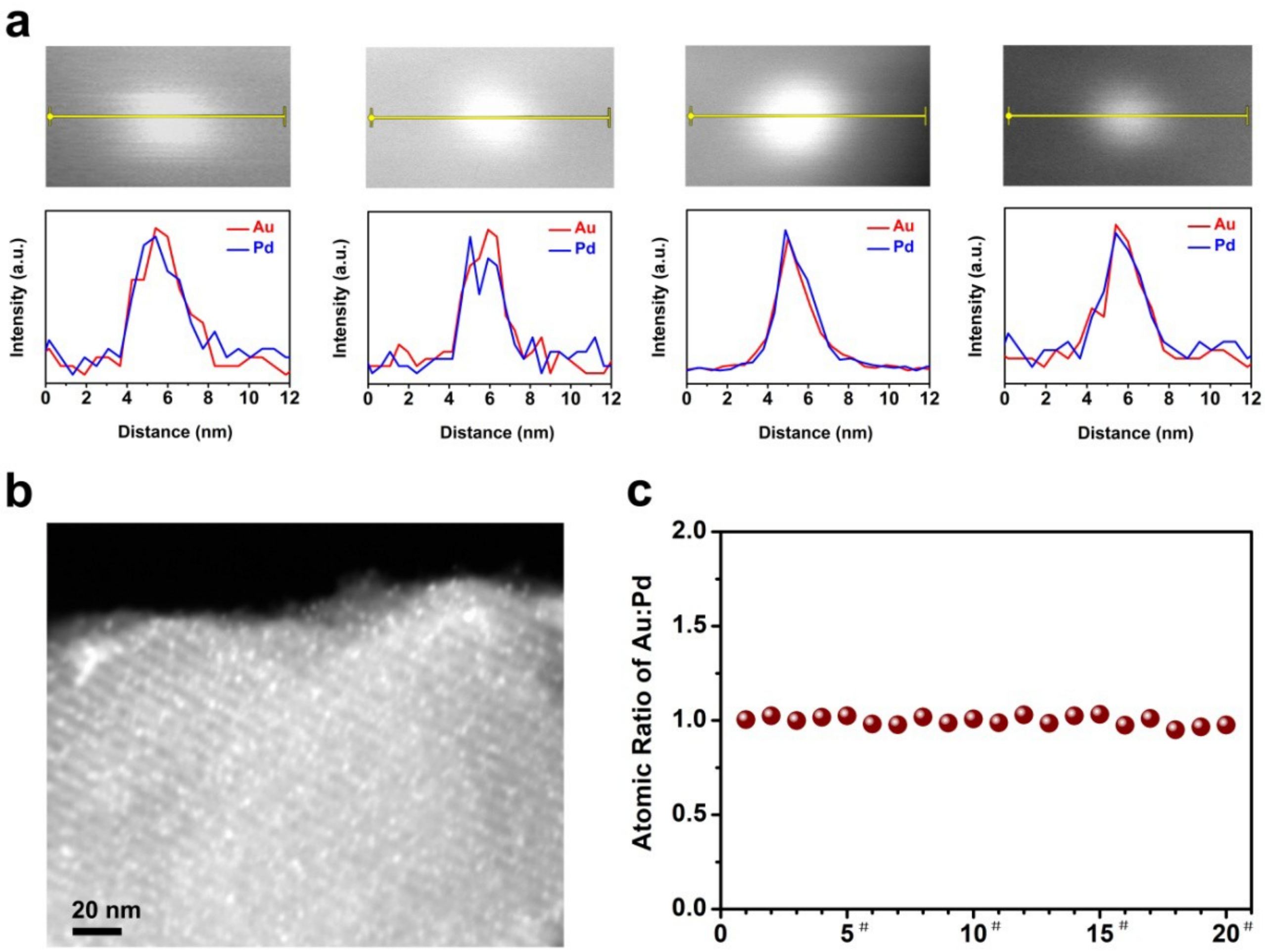

Supplementary Fig. S4. Composition distribution for the reused $Au_{50}Pd_{50}$-R6 catalyst. **a**, Representative line profiles of the EDX patterns of four single particles collected using a focused electron beam in the sub-nanometre range in the STEM mode. **b**, STEM image. **c**, Statics of the individual composition of a single particle collected in different areas. Each dot represents a nanoparticle.

<u>Comment 4</u>. It seems that both Pd and Au in the Pd-Au bimetallic catalysts shift to lower binding energies compared to pure Pd and Au. What causes such shift? If there is an electron flow from one metal to another, their shifts in binding energy should be in opposite directions.

<u>Response 4</u>. We appreciate this insightful comment very much. It is true that the shifts in binding energy for different metal in bimetallic particles should be in opposite directions, if there is an electron flow from one metal to another. However, in the studied AuPd nanoalloy structure, which is similar to the bulk alloy, the redistribution of valence occupations caused by hybridization changes is predominant for explaining the shifts of the binding energy.

For the present AuPd nanoalloys, both the Au and the Pd core levels feature by a shift toward low binding energy compared to the monometallic catalysts. The interesting negative shifts of both the Au 4*f* and Pd 3*d* core level bands in bimetallic species compared to the corresponding monometallic species have been also reported for bulk AuPd alloys[1] and nanoparticles[2], in contrast to Cu-Pd[3], where the Pd shifts are positive and the Cu negative. On consideration of the alloy structure in the present nanoparticles similar to the bulk alloy, we adopt the models as demonstrated in the bulk alloy, in which the redistribution of valence occupations is caused by hybridization changes[1, 4]. In this scheme, the *d* band is active in alloying; the non-*d*-charge (*sp* conduction band, primarily *s* character) flows onto an Au site is positive and is largely compensated by accompanying Au *d* charge depletion; the net charge flow is very small (0.1 electron)[5]. It should be mentioned that the filling of the Pd *d* bands is largely due to changes in the hybridization of Pd *d* bands with the partner element bands[6].

Combined with the XAFS results, hybridization change model can also be drawn in this work due to the strong Au-Pd *d*-*d* interaction. For the Pd-rich alloy, the amount of the charge transfer is in general very small upon alloying, almost less than 0.1 electron in alloys, and the total charge transfer at Pd site is smaller than the change of *d* electron number. The Au atoms appear to gain 0.1 - 0.2 *sp* electrons, compensated by a loss of about the same number of *d* electrons[1]. The intraatomic charge transfer (the *d* and *sp* occupation change) is more important than the interatomic charge transfer between Pd and Au atoms. The maximum *d*-charge gain at approximate 50 *at*% Pd alloy composition results in a pronounced change in the available electronic energy states. With a further reduction in the Pd concentration, the 5*sp* electrons of the surface Pd atoms may lose some *d*-character due to the isolated Pd atoms and the coordination with Au. The *d*-electron number reduces to some extent.

References

1. Nascente, P. A. P., de Castro, S. G. C., Landers, R. & Kleiman, G. G. *Phys. Rev. B* **43,** 4659-4666 (1991).
2. Petkov, V. et al. *J. Phys. Chem. C* **121,** 7854-7866 (2017).
3. Mårtensson, N., Nyholm, R., Calén, H., Hedman, J. & Johansson, B. *Phys. Rev. B* **24,** 1725-1738 (1981).
4. Griffin, M. B. et al. *J. Catal.* **307,** 111-120 (2013).

5. Watson, R. E., Hudis, J. & Perlman, M. L. *Phys. Rev. B* **4,** 4139-4144 (1971).
6. Fuggle, J. C. et al. *Phys. Rev. B* **27,** 2145-2178 (1983).

We have accordingly revised the manuscript.

Revised manuscript, Page 10:

"Models in which the redistribution of occupied valence shells is caused by a difference in the hybridization of Pd and Au appear quite plausible[6]" has been changed to "The negative shifts of both the Au 4*f* and Pd 3*d* core level bands in bimetallic species compared to the corresponding monometallic species have been also reported for bulk AuPd alloys[36], in contrast to Cu-Pd bulk alloys[37], where the Pd shifts are positive and the Cu negative. Models in which the redistribution of occupied valence shells is caused by a difference in the hybridization of Pd and Au appear quite reasonable[38]. In this scheme, the *d* band is active in alloying due to the strong *d*-*d* interaction; the non-*d*-charge (*sp* conduction band, primarily *s* character) flows onto an Au site is largely compensated by accompanying Au *d* charge depletion; the net charge flow is small (0.1 electron)[39]. The detailed discussion is shown in the following part on combination with the XAFS results."

Revised manuscript, Page 15, two sentences have been added:

"The Au atoms appear to gain 0.1 - 0.2 *sp* electrons, compensated by a loss of about the same number of *d* electrons[36]. The intraatomic charge transfer (the *d* and *sp* occupation change) is more important than the interatomic charge transfer between Pd and Au atoms."

Comment 5. It is not clear how they calculated TOF. How do they quantify surface Au and Pd? Counting surface sites is crucial for TOF calculation in order to draw any quantitative conclusions.

Response 5. Thank you very much for the important comments. It is true that counting surface sites is crucial for TOF calculation. The TOF calculation is shown in details as follows.

The studied AuPd solid-solution nanoalloys possess determined structural resolution at an atomic level. The nanoalloy catalysts have uniform compositions throughout the particles, showing negligible difference in compositions between the surface and bulk. As a result, this kind of catalyst can minimize particle size and compositional uniformity effects. To simplify, we use the total surface atoms to estimate the surface Au and Pd atoms on the basis of the atomic ratio.

The total surface sites for Au, Pd, and Au-Pd nanoparticles are estimated from the models, as suggested in

literatures[1-3], including spherical, regular cubeoctahedra or truncated octahedral models. One can assume in these models that both the total number of atoms and the related fraction of surface atoms, in a given particle, can be expressed as a continuous function of their diameter[1-4]. As evidenced in the AC-STEM image, the shape for the presented AuPd nanoparticles are best represented by the top slice of truncated octahedron. Then the dependence on the particle diameter of the number of the various types of atoms is given by the best fit of the calculated values, as shown by Eqs. (1)- (4),

$$d_{\text{particle}} = 1.105\times N_T^{1/3}\times d_{\text{AuPd}} \quad (1)$$

$$N_T = 16m^3\text{-}33m^2\text{+}24m\text{-}6 \quad (2)$$

$$N_S = 30m^2\text{-}60m\text{+}32 \quad (3)$$

$$\tau = N_S/N_T \quad (4)$$

where d_{particle} is the diameter of particle, N_T is the total atom number of each particle, N_S is the surface atom number, and m is the number of atoms lying on an equivalent edge (corner atoms included). Note that the diameter of Au (d_{Au}) and Pd (d_{Pd}) atom is 0.2884 and 0.2751 nm, respectively; and an average atom diameter of 0.2818 nm for d_{AuPd} is adopted.

References

1. Abad, A., Corma, A. & García, H. *Chem. Eur. J.* **14,** 212-222 (2008).
2. Fang, W., Chen, J., Zhang, Q., Deng, W. & Wang, Y. *Chem. Eur. J.* **17,** 1247-1256 (2011).
3. Chen, W. Y. et al. *J. Am. Chem. Soc.* **136,** 16736-16739 (2014).
4. Della Pina, C., Falletta, E., Rossi, M. & Sacco, A. *J. Catal.* **263,** 92-97 (2009).

We have accordingly revised the manuscript.

Revised manuscript, Page 14:

"The estimated turn-over frequency on the basis of exposed Pd-involving sites (TOF_{Pd}) was taken to eliminate the effect of Au dilution." has been changed to "The estimated turn-over frequency on the basis of exposed Pd-involving sites (TOF_{Pd}) was taken to eliminate the effect of Au dilution (Supplementary Fig. S16)."

Revised Supplementary Information, Page 20:

"The turn over frequency (TOF) for each catalyst was calculated on the basis of the estimated number of exposed palladium atoms. This value was calculated at less than 25% conversion,

$$TOF_{Pd} = \frac{n_{BA}X}{n_{Pd}t\tau} \quad (2)$$

where X is the conversion and t is the reaction time. Although microstructural deviations occurred in the AuPd nanoalloys, we assumed that the value for the exposed surface atom dispersion (τ) calculated from equation (3)-(6), which was based on the similarities of the truncated octahedron shape of the nanoparticles (as evidenced in the AC-STEM image) and the homogeneous distribution in the alloy, was accurate[4-7].

$$d_{\text{particle}} = 1.105 \times N_T^{1/3} \times d_{\text{AuPd}} \tag{3}$$

$$N_T = 16m^3 - 33m^2 + 24m - 6 \tag{4}$$

$$N_S = 30m^2 - 60m + 32 \tag{5}$$

$$\tau = N_S / N_T \tag{6}$$

Wherein, d_{particle} is the diameter of particle, N_T is the total atom number of each particle, N_S is the surface atom number, and m is the number of atoms lying on an equivalent edge (corner atoms included). Note that the diameter of Au (d_{Au}) and Pd (d_{Pd}) atom is 0.2884 and 0.2751 nm, respectively, an average atom diameter of 0.2818 nm for d_{AuPd} is adopted."

Comment 6. Gold has been shown as very active catalyst for alcohol oxidations. Maybe the presence of base is crucial. At least they should present their catalytic data on pure Au catalyst synthesized in their work.

Response 6. Thank you very much for the insightful comment. We agree with the reviewer that the presence of base is crucial for the alcohol oxidations on pure gold catalysts, in particular on gold catalysts supported on a less-active porous carbon support. For example, Prati and co-workers used activated carbon as the support to load gold nanoparticles. The catalyst behaves inert under the oxygen pressure at 1.5 atm due to the fact that it is not able to extract the hydride from the alcoholic function[1]. The presence of base can enhance the activity, indicating the H-abstraction step is significantly improved by OH^-[2,3]. Similar results have been reported by our group[4]. The presence of base significantly improves the oxidation of benzyl alcohol to acid. As a result, the oxidation of benzyl alcohol over carbon supported gold nanoparticles is generally carried out in the presence of base[2,5,6].

References

1. Dimitratos, N. et al. *J. Catal.* **244,** 113-121 (2006).
2. Villa, A. et al. *Appl. Catal. A* **364,** 221-228 (2009).
3. Zope, B. N., Hibbitts, D. D., Neurock, M. & Davis, R. J. *Science* **330,** 74-78 (2010).
4. Wang, S. et al. *ACS Catal.* **5,** 797-802 (2015).
5. Yu, X. et al. *Appl. Surf. Sci.* **280,** 450-455 (2013).
6. Yoskamtorn, T. et al. *ACS Catal.* **4,** 3696-3700 (2014).

We have accordingly revised the manuscript.

Revised manuscript, Page 10, several sentences have been added:

"Our results have also shown the content of products generated in the Au-catalysed oxidation of the selected primary alcohols is below the limit of detection, similar to the gold sols loaded on activated carbon catalyst[41,42]. It should be mentioned that the studied phenolic-resin-derived carbon support has a low graphitic degree[43], and serves as a less-active support, and the gold nanoparticles are not able to extract the hydride from the alcoholic function. Once the base is present, the alcoholate can be formed in solution, and the oxidation activity can be significantly improved[44,45]."

Reviewers' comments:

Reviewer #1 (Remarks to the Author):

I am happy that my comments have been addressed and the paper is now suitable for publication. I am surprised there is no CO2 detected but as this is in water maybe the CO2 is dissolved and so not is gas phase for detection. This has been observed in methane oxidation studies in water by Hammond and co-workers.

Reviewer #2 (Remarks to the Author):

The authors replied to my comment reasonably well. They provided new information demonstrating stability of the surface compositions in catalysis. Still they did not reply in a clear way exactly how they counted their surface active sites. Once this notion is added, I do not have further questions.

Reviewer #3 (Remarks to the Author):

It has been improved and should be acceptable.

Responses to Reviewers' comments

Reviewer #1

<u>Comment</u>. I am happy that my comments have been addressed and the paper is now suitable for publication. I am surprised there is no CO_2 detected but as this is in water maybe the CO_2 is dissolved and so not is gas phase for detection. This has been observed in methane oxidation studies in water by Hammond and co-workers.

<u>Response</u>. This comment is highly appreciated. With your kind suggestion, we have referred to the methane oxidation studies in water by Hammond and co-workers[1,2]. They carefully characterize the oxidation products of methane using H_2O_2 as the oxidant including the partially oxygenated products (MeOOH, MeOH, and HCOOH) and deeper oxygenated products (CO_2). They suppose that the hydroxyl radicals (HO ·) from the homolytic cleavage of the HO-OH bond in H_2O_2 can oxidize methanol to HCOOH and CO_2. In the present study, O_2 is used as the oxidant, and the surface adsorbed HO* or HOO* species are supposed to promote the elimination of C^{α}-H in benzyl alcohol. The relatively weak oxidizing ability of surface adsorbed species compared to hydroxyl radicals maybe the reason for the undetectable CO_2.

References

1. Hammond, C. et al. *Angew. Chem. Int. Ed.* **51,** 5129-5133 (2012).
2. Hammond, C. et al. *Chem. Eur. J.* **18,** 15735-15745 (2012).

<u>Accordingly, we have revised our manuscript.</u>

<u>Revised manuscript, Page 14, a sentence has been added:</u>

"In addition, this process is different with the methane oxidation using H_2O_2 as the oxidant, in which the hydroxyl radicals (HO ·) from the homolytic cleavage of the HO-OH bond can oxidize methanol to HCOOH and CO_2[53,54]."

Reviewer #2

<u>Comment</u>. The authors replied to my comment reasonably well. They provided new information demonstrating stability of the surface compositions in catalysis. Still they did not reply in a clear way exactly how they counted their surface active sites. Once this notion is added, I do not have further questions.

Response. We appreciate very much the positive comment, and are delighted that the reviewer kindly highlights the stability of the surface compositions in catalysis. But we are sorry for that we previously solely focused on the stability of the surface compositions in catalysis, and ignored the response for the information on the counting of surface active sites. It is true that counting surface sites is crucial for TOF calculation. The TOF calculation is shown in details as follows.

The studied AuPd solid-solution nanoalloys possess determined structural resolution at an atomic level, as the reviewer mentioned in the previous revision. The nanoalloy catalysts have uniform compositions throughout the particles, showing negligible difference in compositions between the surface and bulk. As a result, this kind of catalyst can minimize particle size and compositional uniformity effects. To simplify, we use the total surface atoms to estimate the surface Au and Pd atoms on the basis of the atomic ratio. The aerobic oxidation of primary alcohols in an aqueous solution in the absence of base is used as a model reaction here because Pd-involving sites are more active than the Au case on a carbon support (the latter is almost inert in this reaction)[1]. In addition, as suggested by the reviewer, the TOFs are regarded reasonable if Pd surface concentrations before and after catalysis are similar. We have confirmed that surface Pd is stable. Pd surface concentrations before and after catalysis are similar. The TOF values normalized to surface Pd concentrations are therefore convincible.

The total surface sites for Au, Pd, and AuPd nanoparticles are estimated from the models, as suggested in literatures[2-4], including spherical, regular cubeoctahedra or truncated octahedral models. One can assume in these models that both the total number of atoms and the related fraction of surface atoms, in a given particle, can be expressed as a continuous function of their diameter[2-5]. As evidenced in the AC-STEM image, the shape for the presented AuPd nanoparticles is best represented by the top slice of truncated octahedron. Then the dependence on the particle diameter of the number of the various types of atoms is given by the best fit of the calculated values, as shown by Eqs. (1)- (4),

$$d_{\text{particle}} = 1.105 \times N_T^{1/3} \times d_{\text{AuPd}} \quad (1)$$

$$N_T = 16m^3-33m^2+24m-6 \quad (2)$$

$$N_S = 30m^2-60m+32 \quad (3)$$

$$\tau = N_S/N_T \quad (4)$$

where d_{particle} is the diameter of particle, N_T is the total atom number of each particle, N_S is the surface atom number, and m is the number of atoms lying on an equivalent edge (corner atoms included). Note that the diameter of Au (d_{Au}) and Pd (d_{Pd}) atom is 0.2884 and 0.2751 nm, respectively; and an average atom diameter of 0.2818 nm for d_{AuPd} is adopted.

References

1. Dimitratos, N. et al. *J. Catal.* **244**, 113-121 (2006).
2. Abad, A., Corma, A. & García, H. *Chem. Eur. J.* **14,** 212-222 (2008).
3. Fang, W., Chen, J., Zhang, Q., Deng, W. & Wang, Y. *Chem. Eur. J.* **17,** 1247-1256 (2011).

4. Chen, W. Y. et al. *J. Am. Chem. Soc.* **136,** 16736-16739 (2014).
5. Della Pina, C., Falletta, E., Rossi, M. & Sacco, A. *J. Catal.* **263,** 92-97 (2009).

We have accordingly revised the manuscript.

Revised manuscript, Page 14:

"The estimated turn-over frequency on the basis of exposed Pd-involving sites (TOF_{Pd}) was taken to eliminate the effect of Au dilution." has been changed to "The estimated turn-over frequency on the basis of exposed Pd-involving sites (TOF_{Pd}) was taken to eliminate the effect of Au dilution (Supplementary Fig. S16)."

Revised manuscript, Page 21, a sentence has been added:

"The TOF value for each catalyst was calculated on the basis of the number of exposed palladium atoms, which was estimated on the similarities of the truncated octahedron shape of the nanoparticles (as evidenced in the AC-STEM image) and the homogeneous distribution in the alloy (Supplementary Fig. S16)."

The calculation of TOF values have been shown in revised Supplementary Information, Page 20:

"The turn over frequency (TOF) for each catalyst was calculated on the basis of the estimated number of exposed palladium atoms. This value was calculated at less than 25% conversion,

$$\mathrm{TOF_{Pd}} = \frac{n_{BA}X}{n_{Pd}t\tau} \quad (2)$$

where X is the conversion and t is the reaction time. Although microstructural deviations occurred in the AuPd nanoalloys, we assumed that the value for the exposed surface atom dispersion (τ) calculated from equation (3)-(6), which was based on the similarities of the truncated octahedron shape of the nanoparticles (as evidenced in the AC-STEM image) and the homogeneous distribution in the alloy, was accurate[4-7].

$$d_{\mathrm{particle}} = 1.105 \times N_T^{1/3} \times d_{\mathrm{AuPd}} \quad (3)$$

$$N_T = 16m^3\text{-}33m^2\text{+}24m\text{-}6 \quad (4)$$

$$N_S = 30m^2\text{-}60m\text{+}32 \quad (5)$$

$$\tau = N_S/N_T \quad (6)$$

Wherein, d_{particle} is the diameter of particle, N_T is the total atom number of each particle, N_S is the surface atom number, and m is the number of atoms lying on an equivalent edge (corner atoms included). Note that the diameter of Au (d_{Au}) and Pd (d_{Pd}) atom is 0.2884 and 0.2751 nm, respectively, an average atom diameter

of 0.2818 nm for d_{AuPd} is adopted."

Reviewer #3

Comment. It has been improved and should be acceptable.

Response. Thank you very much for the positive comment.

REVIEWERS' COMMENTS:

Reviewer #2 (Remarks to the Author):

I have no further comments. The manuscript should now be acceptable by NC.

Response to Reviewer's comment

Reviewer #2

I have no further comments. The manuscript should now be acceptable by NC.

Response. We appreciate very much the positive comment.
